# Interleukin 6 as a Treatment Target for Depression: A Proof-of-Concept Randomized Clinical Trial

**DOI:** 10.1001/jamapsychiatry.2026.1053

**Published:** 2026-05-20

**Authors:** Éimear M. Foley, Nicholas Turner, Ruta Margelyte, Hannah J. Jones, Muzaffer Kaser, Glyn Lewis, Peter B. Jones, Golam M. Khandaker

**Affiliations:** 1 MRC Integrative Epidemiology Unit, Population Health Sciences, Bristol Medical School, University of Bristol, Bristol, United Kingdom; 2 Centre for Academic Mental Health, Population Health Sciences, Bristol Medical School, University of Bristol, Bristol, United Kingdom; 3 Bristol Trials Centre, Bristol Medical School, University of Bristol, Bristol, United Kingdom; 4 NIHR Bristol Biomedical Research Centre, University Hospitals Bristol and Weston NHS Foundation Trust, Bristol, United Kingdom; 5 Department of Psychiatry, University of Cambridge, Cambridge, United Kingdom; 6 Cambridgeshire and Peterborough NHS Trust, Cambridge, United Kingdom; 7 Division of Psychiatry, University College London, London, United Kingdom; 8 Avon and Wiltshire Mental Health Partnership NHS Trust, Bristol, United Kingdom

## Abstract

**Importance:**

Interleukin 6 (IL-6), a keystone inflammatory cytokine, is a credible mechanistic candidate for causing depression. However, randomized clinical trials testing its treatment potential remain scarce.

**Objective:**

To identify likely treatment-sensitive outcomes and effect size for systemic IL-6 inhibition in patients with difficult-to-treat depression.

**Design, Setting, and Participants:**

This 4-week, proof-of-concept, double-blind, parallel-arm, placebo-controlled randomized clinical trial recruited adults with moderate-to-severe *International Statistical Classification of Diseases and Related Health Problems, Tenth Revision (ICD-10) *depression, poor antidepressant response, low-grade systemic inflammation (high-sensitivity C-reactive protein [hs-CRP] level ≥0.3 mg/dL on 2 tests), and depression somatic symptoms (Beck Depression Inventory II somatic symptoms score ≥7) from primary and secondary care and self-referral from 2018 to 2022. Participants were randomized into minimization balanced groups on depression severity and sex. Assessments were conducted at baseline and 7, 14, and 28 days after infusion. Data were analyzed from 2023 to 2025.

**Intervention:**

Single intravenous infusion of IL-6R antagonist tocilizumab (8 mg/kg, maximum 800 mg/patient) or normal saline.

**Main Outcomes and Measures:**

The primary outcome was depression somatic symptoms at 14 days after infusion. The secondary outcome was total depression severity. Exploratory outcomes included fatigue, anxiety, anhedonia, quality of life, and cognition. Outcomes were assessed using validated scales and interpreted against clinically meaningful thresholds.

**Results:**

A total of 30 participants (mean [SD] age, 41.1 [12.3] years; 24 [80.0%] female) were randomized, including 14 in the tocilizumab group and 16 in the placebo group. Of these, 29 participants received the assigned infusion and completed follow-up. As expected for a small proof-of-concept study, no results reached statistical significance, including little improvement in depression somatic symptoms at day 14 (adjusted mean difference: −0.12; 95% CI, −2.51 to 2.28). However, a pattern of greater stepwise improvement over time was observed with tocilizumab in somatic symptoms, depression severity, fatigue, psychological symptoms, state anxiety, and quality of life, with the largest effects observed at the final follow-up (day 28). Tocilizumab may also improve more individual depressive symptoms. Treatment effects were within ranges considered clinically meaningful for depression severity, fatigue, anxiety, and quality of life. At the final follow-up, remission (7 participants [53.9%] vs 5 participants [31.3%]; number needed to treat [NNT] = 5) and response (6 participants [46.2%] vs 3 participants [18.8%]; NNT = 4) rates favored tocilizumab compared with placebo. Baseline hs-CRP level, but not IL-6 level, tracked depression improvement, suggesting hs-CRP may better predict immunotherapy response in depression than drug-specific biomarkers. Tocilizumab was well tolerated, with no serious adverse events or withdrawals.

**Conclusions and Relevance:**

These findings highlight treatment-sensitive outcomes, effect sizes, and patient selection methods for testing systemic IL-6 inhibition in patients with difficult-to-treat depression, and call for a large-scale efficacy trial of anti–IL-6 treatment in depression.

**Trial Registration:**

isrctn.org Identifier: ISRCTN16942542

## Key Points

**Question:**

Can systemic interleukin 6 (IL-6) inhibition with tocilizumab improve depressive symptoms in patients with difficult-to-treat depression and low-grade inflammation?

**Findings:**

This randomized clinical trial of 30 participants, including 14 participants randomized to a tocilizumab infusion compared with 16 participants randomized to a saline placebo infusion, indicated a pattern of greater stepwise improvement in depression severity, somatic symptoms, fatigue, and anxiety, and better remission rate and symptom-level effects, although the study was not powered to detect statistical significance. Improvement corresponded to baseline high-sensitivity C-reactive protein (hs-CRP) but not IL-6, suggesting that hs-CRP may better predict immunotherapy response in depression than drug-specific biomarkers.

**Meaning:**

These findings suggest that the IL-6 or IL-6 receptor pathway could be a new treatment target for inflammation-linked depression, with repeat hs-CRP testing as a practical precision medicine strategy for identifying patients most likely to benefit from immunotherapy.

## Teaser

This proof-of-concept randomized clinical trial identifies likely treatment-sensitive outcomes and effect size for systemic interleukin 6 inhibition in patients with difficult-to-treat depression

## Introduction

Low-grade systemic inflammation is a putative causal factor in depression, present in approximately 30% of patients.^1^ Individuals with difficult-to-treat depression have higher cytokine (eg, interleukin 6 [IL-6]) and C-reactive protein (CRP) levels than treatment-responsive patients^2^ and controls.^3^

Longitudinal^4^ and genetic^5^ studies support a causal role for IL-6 in depression. Experimental IL-6 elevation alters mood and cognition.^6^ Anti-inflammatory drugs improve depressive symptoms,^7^ but their broad mechanisms limit specificity. Anticytokine therapies improve mood in immune-mediated disease independent of physical illness improvement.^8,9^ Somatic-like symptoms show the strongest association with inflammation.^10,11^ However, to our knowledge, no published randomized clinical trials (RCTs) have directly tested the therapeutic potential of IL-6 receptor (IL-6R) blockade in depression.

We conducted a proof-of-concept, double-blind RCT in patients with difficult-to-treat depression and low-grade inflammation to examine the IL-6 or IL-6R pathway as a potential treatment target for depression, identify treatment-sensitive outcomes, gauge effect size and timing of treatment effect, and evaluate feasibility, tolerability, and safety.^12^ We hypothesized that an IL-6R antagonist would attenuate depression somatic symptoms (primary outcome) and total depression severity (secondary outcome).

## Methods

### Trial Design

The Insight Study is a 4-week, proof-of-concept, parallel-group, double-blind, placebo-controlled RCT conducted at the University of Cambridge and University of Bristol, United Kingdom. The South Central Oxford B Research Ethics Committee approved the study. Participants provided written informed consent. People with lived experience contributed to protocol^12^ and material development. The trial protocol and statistical analysis plan are provided in Supplement 1. This study is reported following the Consolidated Standards of Reporting Trials (CONSORT) reporting guideline.

Participants were recruited through National Health Service primary and secondary care and self-referral, supported by the UK National Institute for Health and Care Research (NIHR) Clinical Research Network (eFigure 1 in Supplement 2). Eligibility included persistent low-grade inflammation (high-sensitivity CRP [hs-CRP] ≥0.3 mg/dL [to convert to milligrams per liter, multiply by 10] on 2 tests without infection or immune-related disease), inflammation-related symptoms (Beck Depression Inventory II [BDI-II] somatic symptoms score ≥7), and difficult-to-treat depression (met *International Statistical Classification of Diseases and Related Health Problems, Tenth Revision *[*ICD-10*] criteria for depression despite antidepressant treatment) (eAppendix and eTable 1 in Supplement 2).

### Intervention

Participants were randomized 1:1 to 1 intravenous infusion of tocilizumab (8 mg/kg body weight, maximum 800 mg/patient) or saline. Minimization balanced sex and depression severity. Follow-ups occurred at approximately 7, 14, and 28 days after infusion. Blinding, randomization, and infusion protocols are provided in the eAppendix in Supplement 2.

### Outcomes

The primary outcome was depression somatic symptoms (assessed using BDI-II; range 0-21, with higher score indicating greater presence of somatic symptoms of depression) at 14 days after infusion. The secondary outcome was depression severity (assessed using BDI-II). Exploratory outcomes included fatigue (assessed using the Multidimensional Fatigue Inventory), anhedonia (assessed using the Snaith-Hamilton Pleasure Scale), anxiety (assessed using the State-Trait Anxiety Inventory), quality of life (assessed using the EuroQol 5-dimension 3-level), and cognition (assessed using the Cambridge Neuropsychological Test Automated Battery) (eAppendix, eTable 2, and eTable 3 in Supplement 2). Assessments were conducted at baseline (preinfusion) and days 7, 14, and 28 postinfusion.

### Statistical Analysis

Full details of statistical analysis are provided in the eAppendix in Supplement 2. Multivariable regression examined effect of tocilizumab on outcomes at each follow-up adjusted for baseline score (model 1) and confounders (model 2). Repeated-measures regression assessed symptom trajectories across follow-ups. Risk differences (RDs) and numbers needed to treat (NNTs) were calculated for depression remission and response. Change scores were calculated from baseline to final follow-up per participant for all BDI-II items and group-level medians reported by trial group. This small proof-of-concept study was not powered for definitive efficacy conclusions. All participants who received an infusion were analyzed. The primary focus was on overall pattern of results, not statistical significance, with interpretation based on effect size magnitude, CIs, and pattern of change over time relative to minimal clinically important difference (MCID) thresholds, when available. Analyses were conducted using R software version 4.4.0 (R Project for Statistical Computing) and Stata version 18 (StataCorp) from 2023 to 2025.

## Results

Recruitment occurred from October 2018 to June 2022. A total of 30 participants (mean [SD] age, 41.1 [12.3] years; 24 [80.0%] female) were randomized, including 14 participants to the tocilizumab group and 16 participants to the placebo group; 29 participants received an infusion (eFigure 2 and eAppendix in Supplement 2). Participants had moderate to severe recurrent depression (25 participants [83.3%] with >2 past episodes; 16 participants [53.3%] with >4 past episodes) (eTable 4 in Supplement 2). All participants were using an antidepressant (17 participants [56.7%] using a selective serotonin reuptake inhibitor). Sex-stratified outcome means and SDs are provided in eTable 5 and eTable 6 in Supplement 2.

Baseline hs-CRP was comparable between groups (mean [SD]: tocilizumab 1.02 [0.54] mg/dL; placebo, 0.90 [0.47] mg/dL). Correlation between baseline IL-6 and hs-CRP was *r* = 0.42 (95% CI, 0.06-0.68). Tocilizumab caused rapid and sustained CRP reduction (eAppendix, eTable 7, and eFigure 3 in Supplement 2).

### Primary Outcome

**Figure 1.**
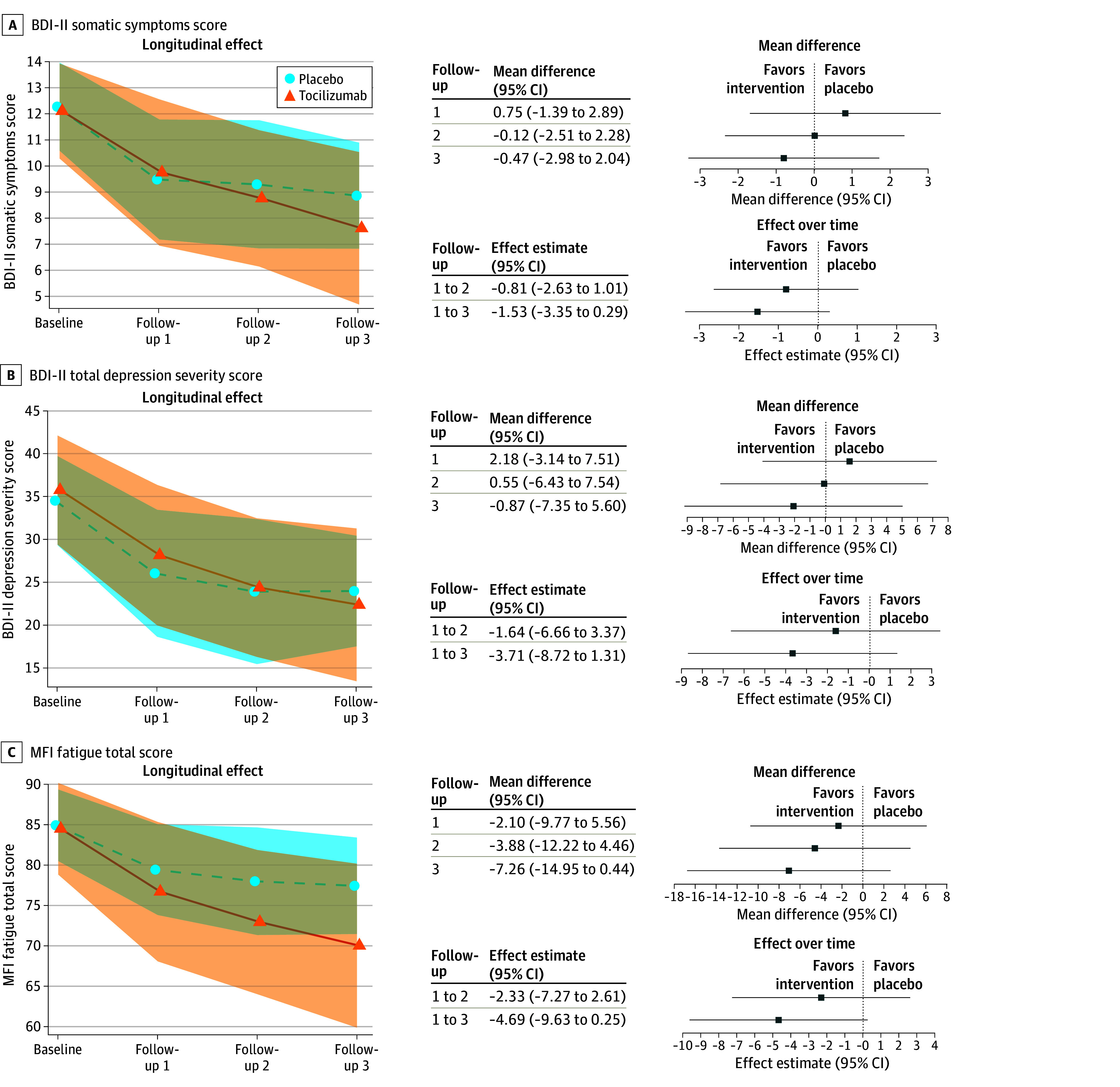
Line Graphs and Point Plots of the Effects of Tocilizumab Treatment on Clinical Outcomes in the Insight Study Longitudinal effect, expressed as means and SDs, are presented from baseline to final follow-up, showing the longitudinal effect of treatment in the drug arm vs the placebo arm. Lower scores indicate lower symptom severity. Mean difference: mean differences represent the difference in the mean outcome score between the trial arms at each follow-up, after adjusting for baseline score and covariates. Negative scores represent a greater reduction in symptom severity in the tocilizumab arm compared to the placebo arm. Effects over time represent the estimated mean change in an outcome per trial arm over time, after adjusting for baseline score and covariates. A negative regression coefficient indicates a more beneficial (greater reduction in symptom severity) treatment effect over time favoring tocilizumab. BDI-II indicates Beck Depression Inventory (range, 0-21; lower scores indicate lower symptom severity); MFI, Multidimensional Fatigue Inventory (range, 20-100; lower score indicates lower symptom severity); shading, SD.

At day 14 after infusion, the mean difference between trial arms in somatic symptoms score was −0.12 (95% CI, −2.51 to 2.28) after controlling for baseline score and covariates (Figure 1; eTable 8 in Supplement 2). The fully adjusted effect estimate comparing symptoms from first to second follow-up between trial arms was −0.81 (95% CI, −2.63 to 1.01), indicating little treatment effect in the first 14 days after infusion.

### Secondary Outcome

The baseline-adjusted mean difference between trial groups at final follow-up in total depression score was −2.10 (95% CI, −9.18 to 4.98), attenuating to −0.87 (95% CI, −7.35 to 5.60) after covariate adjustment (Figure 1; eTable 8 in Supplement 2). The 95% CIs include MCID for BDI-II total score,^13^ but their width indicates uncertainty. Fully adjusted effect estimate comparing symptom change from first to final follow-up was −3.71 (95% CI, −8.72 to 1.31), indicating greater stepwise improvement over time with tocilizumab.

### Effect of Baseline Inflammation

**Figure 2.**
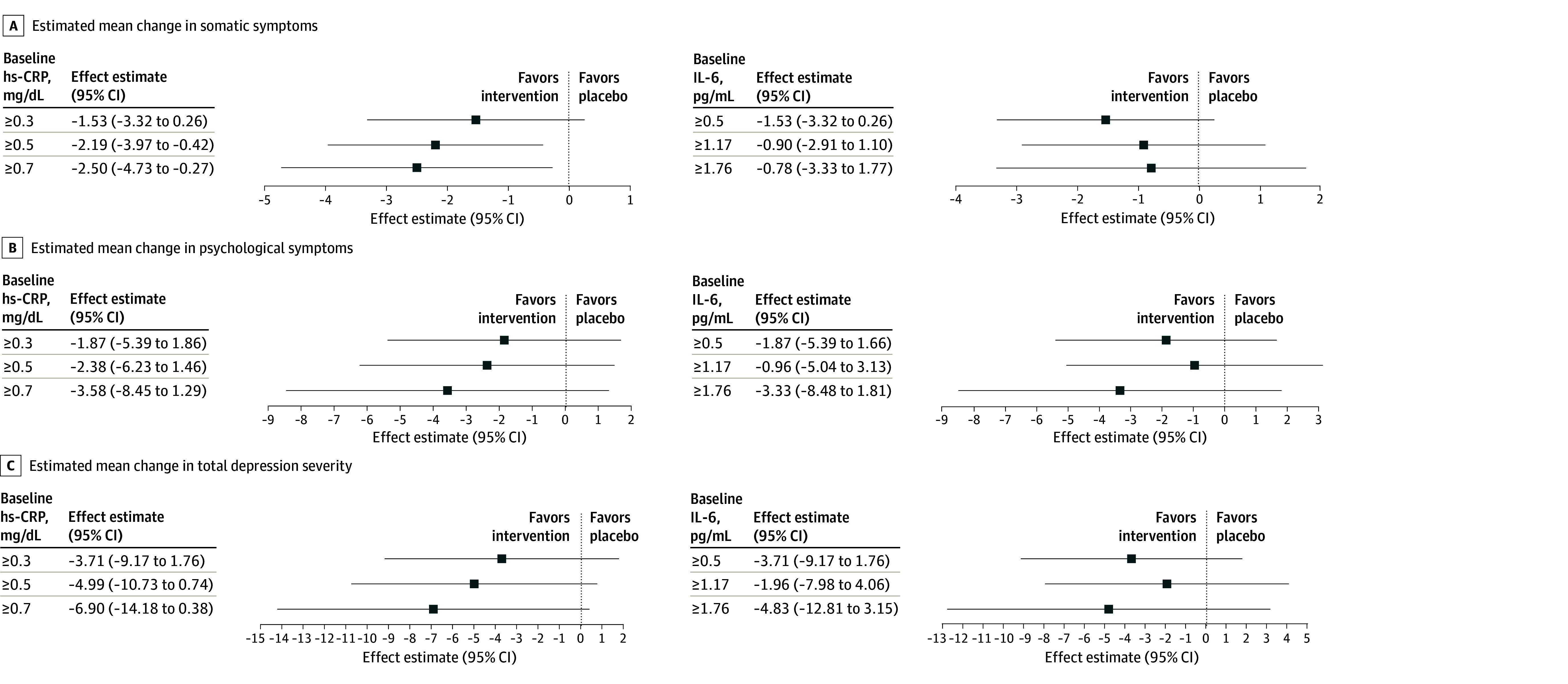
Point Plots of the Effects of Baseline Interleukin-6 (IL-6) and High-Sensitivity C-Reactive Protein (hs-CRP) Concentrations on Treatment Response in the Insight Study Analyses were fully adjusted for baseline score and covariates. Tertiles were chosen to represent low, medium, and high levels of hs-CRP and IL-6 based on the variation of protein levels in our sample. A negative regression coefficient indicates a more beneficial (greater reduction in symptom severity) treatment effect over time favoring tocilizumab. To convert hs-CRP to milligrams per liter, multiply by 10.

Higher baseline hs-CRP was associated with possible greater treatment effects in a stepwise fashion (Figure 2). For somatic symptoms, the fully adjusted effect estimates were −1.53 (95% CI, −3.32 to 0.26) for hs-CRP 0.30 mg/dL or greater, −2.19 (95% CI, −3.97 to −0.42) for hs-CRP 0.50 mg/dL or greater, and −2.50 (95% CI, −4.73 to −0.27) for hs-CRP 0.70 mg/dL or greater. For total depression severity, the estimates were −3.71 (95% CI, −9.17 to 1.76) for hs-CRP 0.30 mg/dL or greater, −4.99 (95% CI, −10.73, 0.74) for hs-CRP 0.50 mg/dL or greater, and −6.90 (95% CI, −14.18 to 0.38) for hs-CRP 0.70 mg/dL or greater. No such pattern was observed for IL-6.

### Depression Remission and Response

At final follow-up, 7 participants (53.9%) in the tocilizumab group met remission criteria vs 5 participants (31.3%) in the placebo group (RD, 0.23 [95% CI, −0.13 to 0.58]); 6 participants (46.2%) in the tocilizumab group and 3 participants (18.8%) in the placebo group met treatment response criteria (RD, 0.27 [95% CI, −0.06 to 0.61]). NNTs for remission and response were 5 and 4, respectively, but CIs included no effect (eAppendix, eTable 9, eTable 10, and eFigure 4 in Supplement 2).

### Individual Symptoms of Depression

**Figure 3.**
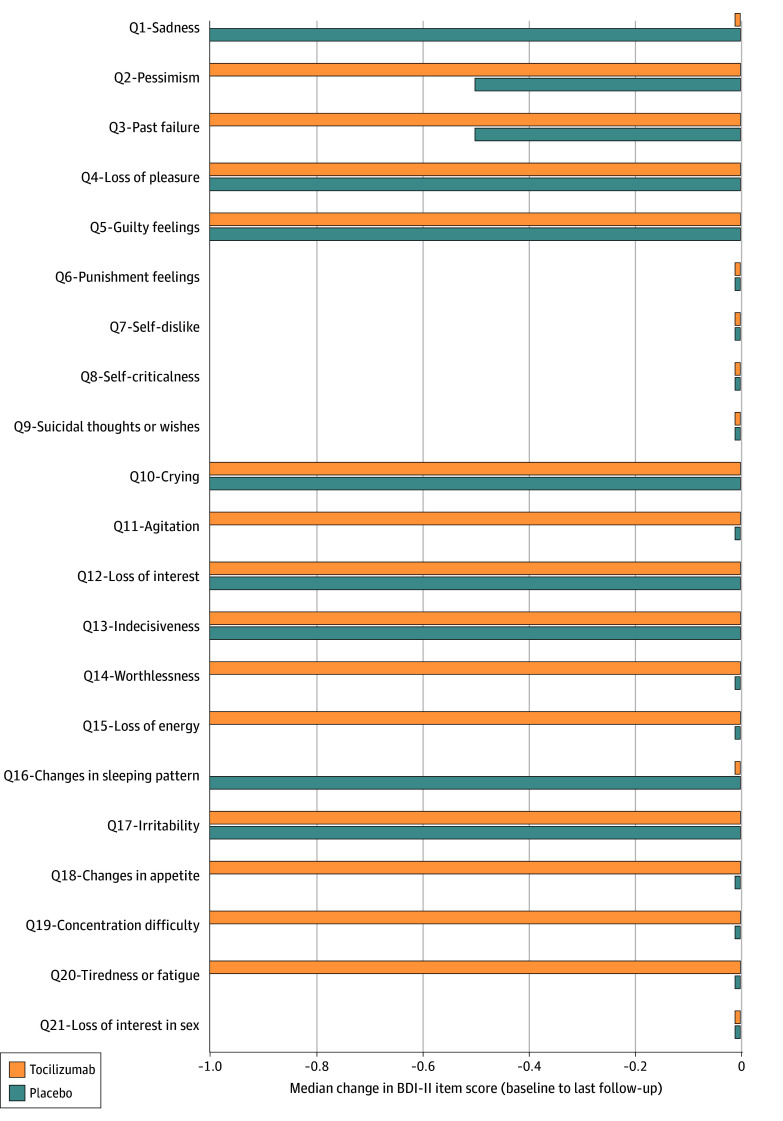
Bar Chart of the Effects of Tocilizumab on Median Change in Individual Symptoms of Depression in the Insight Study Median change scores from baseline to last follow-up (approximately 28 days after infusion) are presented for each individual item on the Beck Depression Inventory-II (BDI-II) scale per trial group. To aid visualization, scores of zero were recoded as −0.01. Negative values reflect symptom improvement.

Tocilizumab seemed to improve 8 BDI-II depressive symptoms (tiredness or fatigue, concentration, appetite, energy, worthlessness, agitation, pessimism, sense of past failure). Placebo improved sadness and sleep (Figure 3; eFigure 5 in Supplement 2).

### Exploratory Outcomes

The fully adjusted effect size comparing change from first to last follow-up for somatic symptoms was −1.53 (95% CI, −3.35 to 0.29), indicating greater stepwise improvement over time with tocilizumab (Figure 1; eTable 8 in Supplement 2). Fatigue showed the largest treatment effect, with a fully adjusted change from first to last follow-up of −4.69 (95% CI, −9.63 to 0.25). Somatic fatigue domains improved more. Improvement was unrelated to baseline hs-CRP and IL-6 (eAppendix and eFigure 6 in Supplement 2). Additionally, tocilizumab may improve psychological symptoms, anxiety, and quality of life, with CIs including recognized MCIDs but also the null. However, tocilizumab may not affect anhedonia or cognition (eAppendix, eTable 5, and eTable 11 in Supplement 2).

### Safety, Blinding, and Sample Size for Future RCTs

No serious adverse events occurred. Minor events were comparable between groups (eTable 12 in Supplement 2). Blinding was successful (eAppendix in Supplement 2). Information on sample size for future RCTs is provided in the eAppendix and eTable 10 in Supplement 2.

## Discussion

This proof-of-concept RCT provides insights into the therapeutic potential of IL-6 or IL-6R inhibition for depression and highlights suitable patient selection methods. Repeat CRP testing is a useful and feasible strategy to enrich samples for sustained low-grade inflammation.^14^ Mean baseline hs-CRP in our sample was much higher (>0.95 mg/dL) than the selection threshold (≥0.30 mg/dL), despite ruling out infection, suggesting repeat testing is more important than choice of cutoff score.

Baseline hs-CRP, not IL-6, tracked improvements in depression in a stepwise fashion, consistent with prior infliximab^14^ and minocycline^15^ trials. This suggests that hs-CRP may predict immunotherapy response in depression better than drug-specific biomarkers.

Tocilizumab may improve clinical depressive outcomes, but not cognition. The CIs for several clinical outcomes included MCIDs, suggesting clinically meaningful improvements cannot be ruled out. Response and remission rates favored tocilizumab.

Symptom-level results indicate broad improvements. Comparable treatment effects on somatic, psychological, and total depression indicate that anti–IL-6R immunotherapy has benefits beyond somatic symptoms. Stepwise improvement over time calls for a longer, larger trial with repeat treatment to confirm efficacy.

### Strengths and Limitations

Strengths of this study include the RCT design and targeted patient selection. Limitations of this study include small sample, short duration, and limited sample diversity. Our results are compatible with anything from no effect to a clinically important effect, and this study is not sufficiently powered to distinguish between these possibilities. Future RCTs should consider balanced recruitment by sex and ethnicity and longer treatment duration.

## Conclusions

This proof-of-concept RCT supports the IL-6 and IL-6R pathway as a treatment target in depression and highlights treatment-sensitive outcomes, likely effect sizes, and patient selection strategies. A large-scale definitive RCT testing IL-6 and IL-6R inhibition in difficult-to-treat depression is now needed. Recruitment and adherence suggest such a trial is feasible.

## Notes

Supplement 1. Trial Protocol and Statistical Analysis Plan

Supplement 2. eAppendix. eTable 1. Inclusion and exclusion criteria for the Insight Study eTable 2. The Insight Study outcome measures and time of assessment eTable 3. Lower and upper detection limits for inflammatory proteins measured in the Insight Study eTable 4. Sociodemographic, clinical, and cognitive characteristics of the Insight Study participants at baseline eTable 5. Clinical and cognitive outcome scores per timepoint split by sex eTable 6. Immune protein concentrations of Insight Study participants at baseline and post-infusion split by sex eTable 7. Immune protein concentrations of Insight Study participants at baseline and post-infusion eTable 8. Effect of tocilizumab treatment on clinical outcomes in the Insight Study eTable 9. Insight Study participants depression severity category at baseline according to BDI-II score eTable 10. Post-hoc analysis of required sample size estimates for depression outcomes for future RCTs eTable 11. Effect of tocilizumab treatment on cognitive outcomes in the Insight Study eTable 12. Adverse events eFigure 1. Overview of design and procedures for the Insight Study eFigure 2. The CONSORT 202537 flow diagram for the Insight trial eFigure 3. Serum hs-CRP, IL-6, sIL-6R, and sgp130 levels at baseline and post-infusion in tocilizumab and placebo arms in the Insight Study eFigure 4. Effect of tocilizumab treatment on depression remission and response eFigure 5. Effect of tocilizumab treatment on individual depressive symptoms in the Insight Study eFigure 6. Effect of baseline IL-6 and hs-CRP concentrations on treatment response in total fatigue score in the Insight Study eReferences

Supplement 3. Data Sharing Statement
