# Supplementary material for: Interleukin 6 as a Treatment Target for Depression: A Proof-of-Concept Randomized Clinical Trial
Source: JAMA Psychiatry. 2026 May 20;83(8):857–63. doi: 10.1001/jamapsychiatry.2026.1053 (PMC13191455; doi:10.1001/jamapsychiatry.2026.1053)
Supplement: Supplement 1. — Trial Protocol and Statistical Analysis Plan [file jamapsychiatry-e261053-s001.pdf]

IL-6 Inhibition in Patients with Depression and Low-grade Inflammation: The Insight Study

Chief Investigator:
Dr Golam Khandaker MBBS, MPhil, MRCPsych, PhD
Wellcome Trust Intermediate Clinical Fellow, Department of Psychiatry, University of Cambridge
Honorary Consultant Psychiatrist, Cambridgeshire and Peterborough NHS Foundation Trust

Study Sponsor:
University of Cambridge
Cambridgeshire and Peterborough NHS Foundation Trust

Funding Agency:
Wellcome Trust

Prepared by: Dr Golam Khandaker and Ms Bianca Oltean, University of Cambridge

Status: Approved by HRA and REC (IRAS ID: 238297; REC reference: 18/SC/0118)

Version Control:

**Version Number**	**Protocol Date**	**Approvals**
5.0	06.03.2019	REC Ref 18/SC/0118 (approved on 24.04.2018)<br>HRA Approved (date 02/05/2018)

Compliance with Good Clinical Practice:

The study will be conducted in compliance with Good Clinical Practice, and applicable regulatory requirements.

Confidentiality Statement:

This document contains scientific information that are privileged or confidential and may not be disclosed unless such disclosure is required by law or regulations. In any event, persons to whom the information is disclosed must be informed that the information is privileged or confidential and may not be further disclosed by them. These restrictions on disclosure will apply equally to all future information supplied to you that is indicated as privileged or confidential.

Table of Contents:

Executive Summary **5**

Overview of Study Design **6**

1.0 Introduction **7**

1.1 General Background and Rationale 7

2.0 Objectives and Hypothesis **11**

2.1 Primary Objective and Primary Hypothesis 11

2.2 Secondary Objective and Secondary Hypothesis 11

3.0 Methods **12**

3.1 Study Design and Rationale 12

3.2 Intervention 13

3.3 Eligibility 14

3.3.1 Inclusion Criteria for All Participants 14

3.3.2 Additional Inclusion Criteria for Intervention Cohort 14

3.3.3 Exclusion Criteria for All Participants 14

3.3.4 Additional Exclusion Criteria for Intervention Cohort 15

3.4 Antidepressant Drug Treatment and Concomitant Therapy 15

3.5 Sample size and Statistical Power 16

3.6 Outcome Measures for the Intervention Cohort 16

3.6.1 Primary Outcome 16

3.6.2 Secondary Outcome 16

3.6.3 Tertiary/exploratory Outcome Measures 16

4.0 Study Procedure **18**

4.1 Participant Identification and Initial Screening 18

4.2 Face-to-face Eligibility Assessment 21

4.3 Additional Safety Measures for the Inflamed Depression Group 21

4.4 Baseline Data Collection 22

4.5 Randomization and Blinding 23

4.6 Intervention Procedure (Day 0) 23

4.7 Follow-up Data Collection (Day 7, 14 and 28 Post-infusion) 24

4.8 Final Safety Check and Exit from Study (Day 42) 25

5.0 Safety Considerations and Monitoring of Adverse Reactions for Infusion **25**

6.0 Materials and Assessments **27**

6.1 Validated Clinical Scales & Questionnaires 27
6.2 Other Questionnaires 30
6.3 Validated Cognitive Assessments 32
6.4 Venous Blood Sampling for Peripheral Immuno-phenotypes 34
6.5 Genotyping 34
7.0 Participant Payment 35
8.0 Informed Consent and Ethical Approval 35
9.0 Data Management and Statistical Analysis 35
9.1 Data Management 35
9.2 Statistical Analysis 35
10.0 Risks 36
11.0 Regulatory Ethics Compliance 38
11.1 Investigator Responsibilities 38
11.2 Independent Ethics Committee or Institutional Review Board 38
11.3 Informed Consent 40
11.4 Privacy of Personal Data 40
11.5 Long-Term Retention of Samples for Additional Future Research 41
11.6 Country Selection 41
12.0 Reference 42
13.0 Amendments 45

Executive Summary:

IL-6 Inhibition in Patients with Depression and Low-grade Inflammation: The Insight Study

Objectives:

The main objectives of the study are to test whether interleukin 6 (IL-6), a pro-inflammatory cytokine, contributes to pathogenesis of depression, and to examine potential mechanisms by which IL-6 affects mood and cognition. A secondary objective is to compare depressed participants with and without evidence of low-grade systemic inflammation.

Study Design and Procedure:

We propose a proof-of-concept, randomized, double blind, placebo-controlled experiment based on approximately 50 depressed participants (intervention cohort) who have evidence of low-grade inflammation (i.e., serum/plasma high sensitivity C-reactive protein (hsCRP) level ≥3mg/L). Participants will be randomized into two groups to receive single intravenous infusion of normal saline (placebo) or tocilizumab (a humanised monoclonal antibody that inhibits IL-6 signalling and is licensed in the UK for treatment of rheumatoid arthritis). Behavioural and cognitive measures and blood samples will be collected at baseline and after infusion around day 7, 14 and 28. Approximately 50 depressed participants without low-grade inflammation (serum/plasma hsCRP level <3mg/L) will complete the same baseline assessments as the intervention cohort allowing us to fulfil the secondary objective.

Study Population:

With support from the Clinical Research Network, identification and recruitment of participants will be carried out from primary care and mental health services covering Cambridgeshire, Suffolk and Norfolk. Eligible participants will be aged 20-65 years, meet ICD-10 criteria for diagnosis of depression and other inclusion/exclusion criteria.

Statistical Methods:

The primary outcome will be change in somatic symptoms of depression in the tocilizumab group compared with placebo at follow-up. Secondary outcomes will include depression severity, cognitive function, and blood-based biomarkers. As an experimental study, we will focus on overall pattern of change after tocilizumab infusion rather than individual tests for statistical significance.

Figure 1: Overview of Study Design

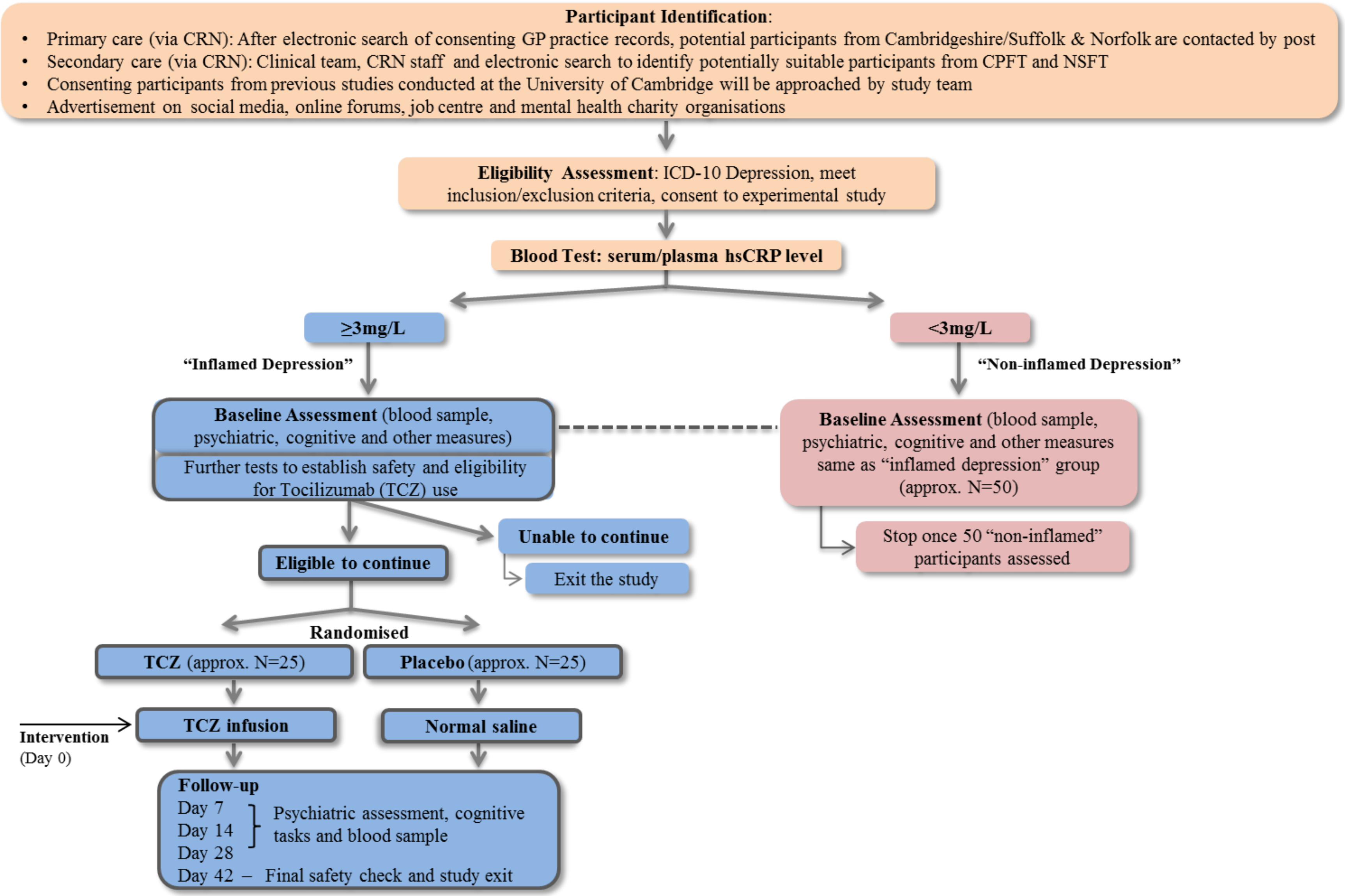

Il-6 Inhibition in Patients with Depression and Low-grade Inflammation: The Insight Study

1.0 Introduction

1.1 General Background and Rationale

Accumulating evidence suggest a role of low-grade systemic inflammation, reflected by elevated concentrations of inflammatory markers circulating in peripheral blood, in pathogenesis of depression. These inflammatory markers include proinflammatory cytokines such as interleukin 6 (IL-6), which are thought to be key mediators of the effect of systemic inflammation on the brain[1,2]. Meta-analysis of cross-sectional studies confirm that concentrations of circulating inflammatory cytokines and acute phase proteins, such as IL-6 and C-reactive protein (CRP), are elevated in depression[3-5], which largely normalise after recovery[5] but continue to be elevated in treatment resistant patients[6,7]. However, it is unclear whether inflammation plays a causal role in depression because cytokine elevation could be a consequence of depression (i.e. reverse causality) or due to confounding.

We have addressed the issue of reverse causality by carrying out a longitudinal study that shows that elevated concentrations of serum IL-6 in childhood are associated with increased risk of developing depression subsequently in early-adulthood in a linear, dose-response fashion[8] (Figure 2). This is one of the first evidence from humans that low-grade systemic inflammation precedes depression, so could potentially be a causal risk factor for the illness. Similarly, other studies have reported that elevated serum IL-6 and CRP levels are associated with subsequent development and persistence of depressive symptoms[9-11].

Figure 2: Prevalence of Depression at Age 18 Years in the ALSPAC Birth Cohort

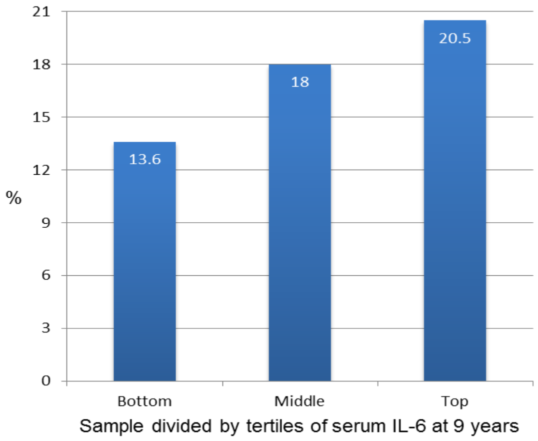

The risk set was divided by tertiles of serum IL-6 levels in all participants at age 9 years (bottom, middle, top). Risk of depression at 18 years was higher for those in the middle and top thirds of IL-6 distribution at age 9 years compared with the bottom third.

Khandaker *et al.*
JAMA Psychiatry 2014

Next, to address the issue of confounding we have carried out a genetic association analysis informed by Mendelian randomization (MR). MR is based on the idea that if a biomarker is causally related to an illness, genetic variant(s) regulating levels/activity of that biomarker should also be associated with the illness[12]. Genetic variants segregate at random during meiosis and are unrelated to sociodemographic and other confounders, so demonstrating an association between depression and genetic variant(s) that regulate IL-6 activity would strongly indicate that IL-6 plays a causal role in the illness. Using data from the ALSPAC birth cohort, we have shown that a genetic variant in the IL-6 receptor gene (*IL6R* Asp358Ala) that is known to dampen down inflammation by impairing the activity of IL-6 is protective for severe depression and/or psychosis[13]. The genetic variant is associated with serum IL-6 and CRP levels, but not with any common confounders of the inflammation-depression relationship such as sex, social class, ethnicity, body mass. While these findings strongly support that IL-6/IL-6R pathways are involved in pathogenesis of depression, observational studies cannot confirm causality. Experimental studies are needed to test directly the effect of manipulation of IL-6 signaling on depressive symptoms in individuals with depression to confirm whether IL-6 plays a causal role in pathogenesis of depression, but such studies are lacking. These studies are also needed to understand the mechanisms by which IL-6 modulation may improve depressive symptoms.

Experimental studies based on healthy volunteers and animal models support a role of inflammation in pathogenesis of depression. Inducing inflammation in healthy volunteers with a typhoid vaccine leads to depressive symptoms and reduced cognitive performance, which is mediated by increase in circulating IL-6 levels[1]. In mice, lipopolysaccharide-induced systemic inflammation leads to depression-like behaviour through activation of the tryptophan metabolizing enzyme indoleamine 2,3-dioxygenase (IDO)[14,15]. Blocking inflammation with minocycline, a broad-spectrum anti-inflammatory agent, attenuates lipopolysaccharide-induced expression of proinflammatory cytokines, normalizes plasma kynurenine/tryptophan ratio, and prevents development of depression-like behaviour[14]. Furthermore, blocking IL-6 directly by injecting a monoclonal antibody prevents stress-induced depression-like behaviour in mice[16]. These findings are consistent with the idea that inflammation in general and IL-6 in particular is involved in pathogenesis of depression. In patients with rheumatoid arthritis anti-inflammatory drugs reduce severity of depressive symptoms[17]. However, to our knowledge no study has examined whether IL-6 inhibition affect depressive symptoms, inflammatory marker concentrations, and IDO activation in

individuals with depression, which is necessary to gain a better understanding of the role of inflammation particularly IL-6 in pathogenesis of depression.

Depression consists of wide ranging symptoms yet most studies have considered it as a categorical outcome[3-5], so it is not fully clear to what extent inflammatory cytokines such as IL-6 contribute to different symptoms of depression. Cytokines are more likely to be relevant for somatic symptoms of depression (e.g. fatigue, appetite and sleep disturbance) rather than psychological symptoms (e.g. hopelessness). These somatic symptoms develop rapidly in majority of interferon-treated cancer patients who develop depression (an established human model for inflammation-induced depression), but cognitive and affective symptoms (e.g. impaired memory, low mood) develop slowly and relatively less frequently[18,19]. Population-based studies have shown that elevated serum IL-6 and CRP levels are associated with fatigue, impaired sleep, but not with hopelessness[20,21]. Furthermore, fatigue is associated with nearly three-fold risk of new-onset depression in future[22]. Cytokine-induced somatic symptoms may affect mood by reducing rewarding experiences[23], so could be an important mediator of the relationship between inflammation and depression. However, to our knowledge no experimental study has examined the effects of reducing IL-6 activity on somatic symptoms specifically in individuals with depression.

Experimental studies of IL-6 modulation in humans should focus on depressed participants who have evidence of low-grade systemic inflammation and a history of poor response to antidepressant. This is because individuals with depression who do not get better with antidepressants are more likely to show evidence of low-grade inflammation[24]. Low-grade inflammation may contribute to resistance to antidepressants[27,28]. A randomised controlled trial (RCT) of infliximab, an anti-TNF monoclonal antibody and anti-inflammatory drug, has reported that the drug is more likely to improve depressive symptoms in patients with treatment resistant depression who have elevated CRP levels (i.e., evidence of low-grade inflammation) at baseline[25].

We propose a proof-of-concept, randomised, double blind, placebo-controlled experimental medicine study to test whether IL-6 contributes to pathogenesis of depression, and to examine potential mechanisms by which IL-6 affects mood and cognition. We propose that inhibition of IL-6 signalling in individuals with depression who show evidence of low-grade inflammation and have history of poor response to antidepressants would attenuate their

depressive symptoms particularly somatic symptoms of depression, reduce concentrations of inflammatory markers, and reduce IDO activation.

2.0 Objectives and Hypothesis

2.1 Primary Objective and Primary Hypothesis

- To carry out a proof-of-concept, randomized, double blind, placebo-controlled experimental medicine study to test whether IL-6 contributes to pathogenesis of depression, and to examine potential mechanisms by which IL-6 affects mood and cognition.

- We hypothesise that inhibition of IL-6 signaling with single intravenous infusion of anti-IL6R monoclonal antibody tocilizumab would attenuate somatic symptoms of depression, reduce serum proinflammatory cytokine levels and IDO activation in individuals with depression who show evidence of low-grade inflammation, i.e., serum/plasma hsCRP level ≥3mg/L (hereafter referred to as “inflamed depression”) and have history of poor response to antidepressants.

2.2 Secondary Objective and Secondary Hypothesis

- To carry out a secondary, observational study to examine differences and similarities between inflamed and non-inflamed depression.

- We hypothesise that individuals with inflamed depression, compared with non-inflamed, will be more likely to have increased somatic symptoms of depression, higher levels of serum proinflammatory cytokines, and evidence of IDO activation.

3.0 Methods

3.1 Study Design and Rationale

The overview of the study design has been presented in Figure 1. We propose to test whether inhibiting IL-6 using a single dose of tocilizumab (a monoclonal antibody against IL-6 receptor) modulates depressive symptoms in adults with inflamed depression. The Health Research Authority (HRA) has confirmed that the proposed experimental medicine study using tocilizumab would be classed as a basic science study involving procedures with human participants, *not* a clinical trial of investigational medicinal product. The study does not aim to ascertain or verify/compare the safety of the medicine: safety of tocilizumab in humans is already established. Tocilizumab is licensed in the UK for treatment of rheumatoid arthritis and juvenile idiopathic arthritis. The study also does not aim to ascertain or verify/compare the efficacy of the medicine in the clinical treatment of depression: to assess efficacy in relation to depression would not be valid or possible with a single-dose study, but rather would require chronic dosing and inclusion of clinically-accepted efficacy outcome measures, neither of which is the case with this protocol (see below). The key outcome measures for this single dose experimental medicine study will be intermediate markers of response with regards to depression, such as somatic symptoms, various aspects of cognitive function, and blood-based biomarkers.

This randomized, double blind, placebo-controlled experiment will be based on approximately 50 participants with inflamed depression (intervention cohort) who will be randomized into two groups:

- Approximately 25 participants with *inflamed depression* will receive one intravenous infusion of tocilizumab (drug)
- Approximately 25 participants with *inflamed depression* will receive one intravenous infusion of normal saline (placebo)

For the secondary, observational study, we will compare baseline characteristics of the intervention cohort (inflamed depression) with approximately 50 participants with non-inflamed depression. Because about a third of individuals with depression show evidence of inflammation,[24] eligibility assessment for the experimental medicine study will identify many individuals with depression who are non-inflamed. The non-inflamed participants will attend

the same baseline assessments as the intervention cohort to fulfil the secondary objective of this study. They will not be randomised, as they will not receive any intervention.

3.2 Intervention

Single intravenous infusion of tocilizumab (8mgs/kg; max 800mgs in total) or normal saline. Tocilizumab is the first-in-class, anti-IL-6R humanized monoclonal antibody, commercially available and licensed in the UK for treatment of rheumatoid arthritis and juvenile idiopathic arthritis. The approved dosage of tocilizumab for treatment of rheumatoid arthritis is 2, 4 or 8mg/kg; max 800mgs in total. In arthritis patients, a single tocilizumab infusion has shown to improve clinical and laboratory measures including CRP within 48 hours, with most noticeable result in one-to-two weeks[26,27]. The follow-up schedule for our study (see below) is in keeping with this observation.

Tocilizumab blocks both IL-6 classic and trans-signaling (responsible for most of the inflammatory effects of IL-6) making it the agent of choice for complete IL-6 inhibition[28]. As justified by interferon[29] and mouse[14] studies, peripheral inflammation cause depression because IL-6 and other circulating cytokines can influence the brain using neural, humoral and cellular pathways[1,2,30,31]. Therefore, tocilizumab, which is mostly peripherally acting, is likely to have an impact on symptoms of depression. Infliximab, an anti-TNF-α monoclonal antibody, that has similar, limited blood-brain barrier penetration as tocilizumab has been reported to reduce symptoms of depression[25].

3.3 Eligibility

3.3.1 Inclusion Criteria for All Participants

- Able and willing to give informed consent, including consent to share information with the participant's General Practitioner (GP) and to access GP records.
- Able to understand written and spoken English
- Able to consent to blood sampling
- Willing to abstain from strenuous exercise for 72 hours before the assessment visits
- **Age:** 20-65 years (inclusive) at the time of eligibility assessment
- **Diagnosis of depression:** meet ICD-10 criteria for diagnosis of depression at the time of eligibility assessment
- **Somatic symptom score:** ≥7 at the time of eligibility assessment based on Beck depression inventory II (BDI-II) items 4=lack of pleasure, 15=loss of energy, 16=changes in sleeping pattern, 18=changes in appetite, 19=concentration difficulty, 20=tiredness or fatigue, and 21=loss of interest in sex.
- **History of non/slow response to antidepressant:** at the time of eligibility assessment receiving treatment with an antidepressant at adequate dose (according to BNF) for at least four weeks.

3.3.2 Additional Inclusion Criteria for Intervention Cohort

- **Inflamed:** Serum/plasma hsCRP level ≥3mg/L.

3.3.3 Exclusion Criteria for All Participants

- Current or lifetime diagnosis of bipolar disorder, psychotic disorder, personality disorder (depression with psychotic symptoms is not exclusionary)
- Currently active diagnosed eating disorder likely to compromise ability to take part (determined by Chief Investigator)
- Current suicidal thoughts (BDI-II item 9=suicidal thoughts or wishes score 3) *or* history of suicide attempt, deliberate self-harm, overdose within six months prior to eligibility assessment
- History of alcohol or substance use disorder (abuse/dependence) within six months prior to eligibility assessment (nicotine and caffeine dependence are not exclusionary)
- Pregnant or breast feeding
- History of serious allergic reaction after any infusion

- Current use of medication likely to compromise interpretation of immunological data (including, but not limited to, antibiotics, non-steroidal anti-inflammatory drugs, oral/injectable corticosteroids – or any other substances to be determined by the Chief Investigator).
- Any major episode of infection requiring hospitalization or treatment with IV antibiotics within 4 weeks of eligibility assessment.
- Known active current or history of recurrent bacterial, viral, fungal, mycobacterial or other opportunistic infections.
- Unstable cardiac, pulmonary, renal, hepatic, endocrine, hematologic, or active infectious disease, including current or prior malignancy.
- Rheumatic autoimmune disease, mixed connective tissue disease, scleroderma, polymyositis, or significant systemic involvement secondary to rheumatoid arthritis.
- Uncontrolled hypertension defined as systolic blood pressure > 170 or diastolic blood pressure > 110.
- No history of chicken pox infection or no history of varicella zoster vaccination

3.3.4 Additional Exclusion Criteria for Intervention Cohort

- Current or past infection with TB, Hepatitis B, Hepatitis C, VZV or HIV confirmed by blood/other test (see Table 1 for a complete list of blood tests). Chest X-ray will be also done to exclude TB.
- Pregnancy test (for female participants).
- History of severe allergic or anaphylactic reactions to human, humanized or murine monoclonal antibodies.

3.4 Antidepressant Drug Treatment and Concomitant Therapy

Participants will continue to receive their existing oral antidepressant and other psychiatric medications and treatments during the study. Adjustment to the antidepressant dosing regimen, if necessary, will be allowed as clinically indicated and will be documented. History of antidepressant treatment, including dose, duration, response and side effects, will be recorded at baseline data collection assessment. All concomitant therapies (e.g. prescription or over-the-counter medications, including vaccines, vitamins, herbal supplements, non-pharmacologic therapies such as electrical stimulation, acupuncture, special diets, exercise

regimens) will be recorded throughout the study. Modification of an effective pre-existing therapy should not be made for the explicit purpose of entering a participant into the study.

3.5 Sample size and Statistical Power

With 50 participants the experimental medicine study will have 80% statistical power (alpha=0.05) to detect a 2.5-point reduction in clinical interview schedule revised (CIS-R) depression severity score in tocilizumab group compared with placebo; mean (SD) for outcome=15(3) based on a previous RCT of depression[32]. We believe the actual sample size needed for primary outcome, i.e., to detect a change in BDI-II somatic symptom score, will be smaller because somatic symptoms are influenced by inflammation more than other depressive symptoms. However, no existing studies are available to inform a power calculation specifically for change in somatic symptoms.

3.6 Outcome Measures for the Intervention Cohort

3.6.1 **Primary Outcome:** Change in total somatic symptoms score from baseline assessment at around day 14 post-infusion. Somatic symptom score will be constructed by summing scores for seven relevant BDI-II items (4=lack of pleasure, 15=loss of energy, 16=changes in sleeping pattern, 18=changes in appetite, 19=concentration difficulty, 20=tiredness or fatigue, and 21=loss of interest in sex).

3.6.2 **Secondary Outcomes:** Change in total depression severity score from baseline assessment at around day 14 post-infusion assessed by BDI-II.

3.6.3 Tertiary/exploratory Outcome Measures:

Behavioural:

- Fatigue
- Anhedonia

Cognitive:

- Psychomotor speed
- Attention and Memory
- Emotional processing

Blood Biomarkers:

- Inflammatory markers, cortisol

- Markers of IDO activation
- Cortisol and glucocorticoid receptor sensitivity
- Cardio-metabolic markers
- White blood cell differential count
- Cell phenotyping: Fluorescence-activated cell sorting of peripheral blood mononuclear cell populations (PBMC)

Genetic:

- Gene expression analysis in whole blood and isolated PBMC

4.0 Study Procedure

A schematic diagram of study procedure has been presented in Figure 3. Participants will be asked to sign an Informed Consent Form after they have been fully informed about the study. No study-related procedures will be performed until consent has been obtained. Participants will be asked to consent to be re-contacted based on the blood test for CRP level for subsequent assessment.

4.1 Participant Identification and Initial Screening

- **Secondary Care:** Participants will be recruited mainly from this source. NHS mental health trusts covering Cambridgeshire and neighbouring areas, i.e., Cambridgeshire and Peterborough NHS Foundation Trust (CPFT), Norfolk and Suffolk NHS Foundation Trust (NSFT), will be included. Participants will be identified by their treating team/clinicians and via the Cambridgeshire & Peterborough NHS Foundation Trust (CPFT) Research Database (17/EE/0442), which contains linked anonymous information derived from CPFT clinical records. The database will be queried to find patients that are likely to meet the study's eligibility criteria, yielding a list of research identification numbers. This list will be submitted to the CPFT Research Database Manager, who will enter them into an automated system that can identify the patients. A template form requesting further information about the patient will be attached. Each request regarding study participation will be passed to the patient's clinical team. For patients who are approached by their clinicians and who give specific consent, and only for those patients, the Research Database Manager will provide the patient's details to the research team, and authorise them to view the patient's CPFT clinical records and to contact the patient to discuss participation in the study. Study participation itself, or provision of any additional information, would require the patient's further consent. In addition, Cambridge Clinical Research Centre for Affective Disorders (C2:AD, REC ID; 15/EE/0305) maintains a database of participants with affective disorders within CPFT. These participants have given consent for contact for future studies. This database will be screened by clinical staff involved in the database management to identify potentially eligible participants.

- **Primary Care**: Primary Care Clinical Research Network (CRN) will identify GP surgeries in Cambridgeshire, Suffolk and Norfolk that are willing to take part.

Electronic search of GP databases will be carried out by GP surgery staff to identify potentially eligible participants, who will be first approached by their clinicians. A follow-up letter will be sent approximately three-to-four weeks after the initial mail invitation to participants who have not responded.

- **Previous studies**: We will contact potentially eligible participants who took part in previous studies at the University of Cambridge, and gave informed consent to be contacted for subsequent studies.
- **Advertisement**: We will advertise on social media, online forums, job centre and mental health charity organizations for potential participants.

As part of initial screening, potentially eligible participants identified from a source other than NHS mental health trusts will be asked to complete a screening questionnaire for depression, e.g. patient health questionnaire 9 (PHQ-9), and a screening questionnaire containing key inclusion/exclusion criteria. Those scoring >7 on the PHQ-9 will be invited for a full, face-to-face eligibility assessment. Potentially eligible participants identified from Primary and Secondary Care will be sent a separate postal screening questionnaire.

Figure 3: Detailed Study Procedure

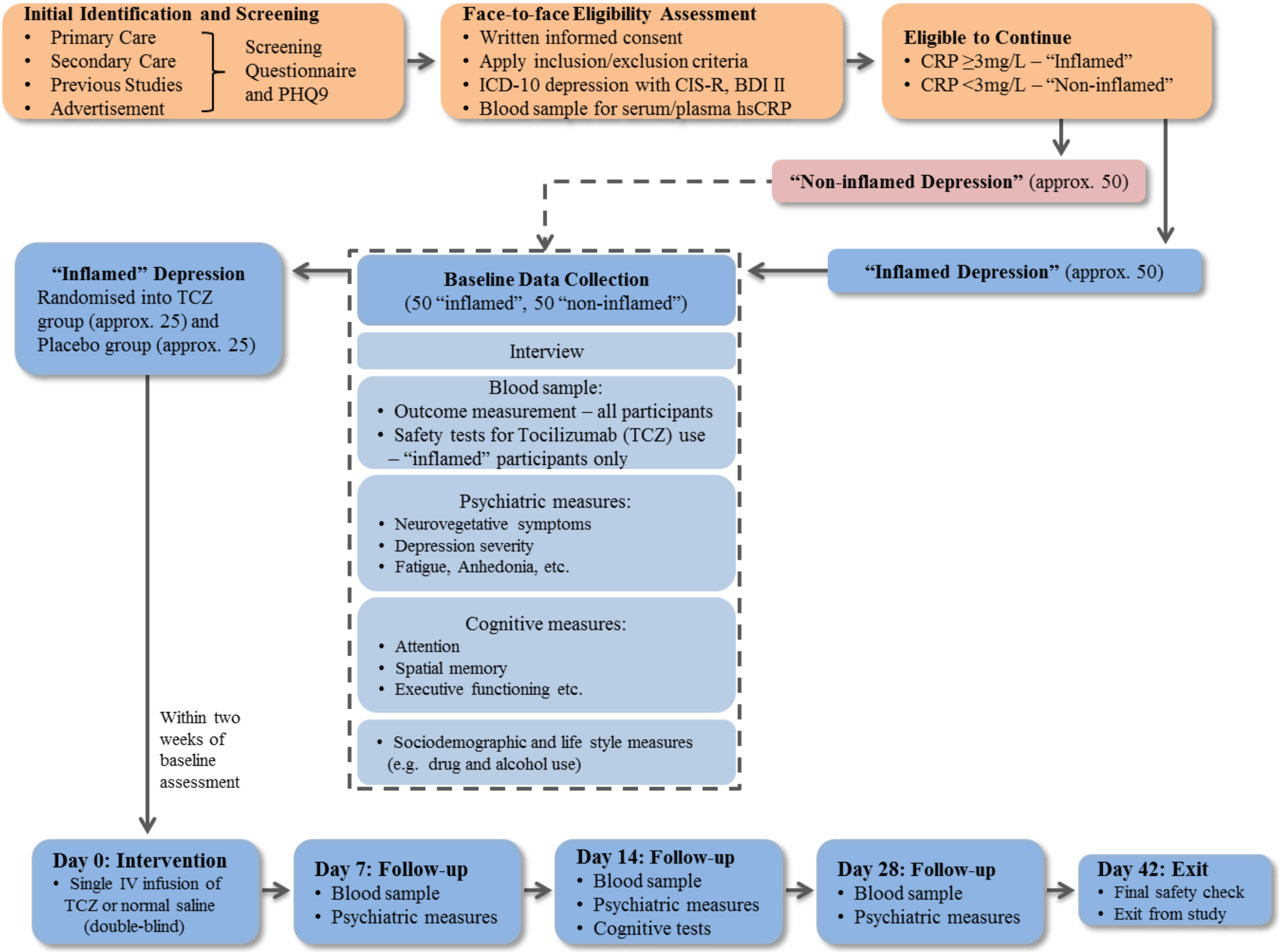

4.2 Face-to-face Eligibility Assessment

After initial screening, potentially suitable participants will be invited to a face-to-face assessment. Depending on participant's preference, this visit will take place at Herschel Smith Building Clinical Research Facility (CRF) at Cambridge biomedical campus *or* Windsor Research Unit at Fulbourn hospital *or* a suitable local facility such as GP surgery, mental health team base *or* at home. The assessment will be conducted by a member of the study team or CRN staff, and will last approximately one hour including blood sampling.

Procedure and Assessment:

1. Explain study further, give opportunity to ask questions, provide Information Sheet
2. Informed Consent Form including consent to contact GP to inform about participation in study, to verify medical history with GP to establish eligibility, and to inform GP any results/outcomes as necessary. Consent for additional tests to establish safety for tocilizumab infusion will be obtained.
3. CIS-R questionnaire to confirm ICD-10 diagnosis of depression, and BDI-II
4. Interview to assess eligibility as per inclusion/exclusion criteria. The study team will also liaise with GP and psychiatrists where applicable to confirm relevant medical history.
5. Blood sampling: After completion of questionnaires/interview, a trained staff will take a non-fasting venous blood sample (5ml approximately). With consent blood sampling can be done separately before or after the face-to-face eligibility assessment via GP, clinical team, CRN/study team if necessary. This can be done, for example, to allow a participant to recover from an infection, such as common cold, or to ease pressure on participant's time. The blood sample will be sent to a local laboratory for measurement of serum/plasma hsCRP level.

4.3 Additional Safety Measures for the Inflamed Depression Group

At the baseline data collection assessment, participants with *inflamed* depression meeting inclusion and exclusion criteria will be screened further to ensure safety for tocilizumab use. Such tests would include, but are not limited to, blood/other tests for Tuberculosis, HIV, VZV, Hepatitis B and C. A member of the study team will explain the purpose of these tests. We will inform all participants the results of their tests. In case a participant tests positive for any of these infections, we will inform their GP requesting a referral to appropriate infectious disease department.

4.4 Baseline Data Collection

Participants with depression, approximately 50 inflamed and 50 non-inflamed, will be assessed at Herschel Smith Building CRF at Cambridge biomedical campus *or* Windsor Research Unit at Fulbourn hospital *or* a suitable local facility such as GP surgery, mental health team base *or* at home according to participant's preference. The visit will be conducted by a member of the study team/CRN staff and will last approximately 2 hours. Participants will be allowed regular breaks; refreshments will be provided. Procedure of the baseline data collection visit including assessments has been listed below. The assessment scales and questionnaires will be computerised where possible.

Procedure:

1. Schedule for this assessment will be explained to participants.
2. Physical measurements including height, weight, waist circumference, systolic and diastolic blood pressure, pulse.
3. Blood samples: will be taken by a trained staff (approximately 50ml – see Table 1).
4. Questionnaire to record sociodemographic information, antidepressant history, concomitant treatment, and use of tobacco, alcohol and illicit substances
5. Psychiatric measures (questionnaires):
    a. Somatic symptoms and depression severity assessed by BDI-II
    b. Anhedonia assessed by Snaith-Hamilton Pleasure Scale Questionnaire
    c. State-Trait Anxiety Inventory (STAI)
    d. Fatigue assessed by Multi-dimensional Fatigue Inventory
    e. Visual Analogue Scales for Subjective Wellbeing (VAS-W)
    f. Self-Compassion Scale – Short Form (SCS-SF)
    g. Perceived Stress Scale (PSS)
    h. European Quality of Life-5 Dimensions (EQ-5D)
6. Cognitive tests:
    a. National Adult Reading Scale for estimated premorbid IQ
    b. Psychomotor speed:
        i. CANTAB Reaction Time (RTI) test or a similar test (computerised)
        ii. Digit Symbol Substitution Test (pen and paper) or a similar test
    c. Attention: CANTAB Rapid Visual Information Processing (RVP) test or a similar test (computerised)

    d. Memory: CANTAB Paired Associates Learning (PAL) test or a similar test (computerised)
    e. Executive Function: CANTAB One Touch Stockings of Cambridge (OTS) test or a similar test (computerised)
    f. Emotional Processing:
        i. CANTAB Emotional Bias Task (EBT) or a similar test (computerised)
        ii. Emotional categorization and recall task or a similar task (computerised)

At the end of the session, there will be time for Q&A and for filling in the expense form. Participants with inflamed depression will be scheduled for the intervention visit within next 14 days. Non-inflamed participants will be debriefed as this will be their final visit.

4.5 Randomization and Blinding

Randomization will be done by an external agency. Participants will be randomly assigned to tocilizumab or normal saline group ensuring two groups are comparable to each other on depression severity and sex. Pharmacy staff at the Central Pharmacy, Addenbrooke's hospital, Cambridge University Hospitals NHS Foundation Trust, dedicated to handle research studies including clinical trials will dispense tocilizumab and normal saline according to the randomization schedule. Infusions will be prepared at a CRF in Cambridge Biomedical Campus where infusion will take place by a member of staff external to the study team. Infusion packs containing drug and placebo will be visually indistinguishable from each other ensuring both participants and study team remain blind about allocation of intervention.

4.6 Intervention Procedure (Day 0)

The intervention visit will take place at a CRF in Cambridge Biomedical Campus and will last approximately two and half hours.

Procedure:

1. On arrival, a member of study team will explain the schedule for this visit and confirm participants are happy to proceed. Interview to check it is safe to proceed with infusion, e.g., participants have not developed an infection. Female Participants

of childbearing age will be given a urine pregnancy test. Participants who are sexually active will be asked to use contraception for six weeks after infusion.

2. Intravenous infusion: an unlabelled (i.e. double blind) infusion of tocilizumab *or* normal saline will be administered by a trained CRF staff under supervision of a designated study doctor. The infusion will be administered continuously over one hour and the participants will be supervised by a nurse or a member of the study team at all times. Participants will remain in CRF under observation for a further one-hour period after the end of infusion. Refreshments will be available during this time. See below for plans for monitoring and managing potential side effects.

4.7 Follow-up Data Collection (Day 7, 14 and 28 Post-infusion)

The follow-up assessments will take place approximately 7, 14 and 28 days after infusion at Herschel Smith Building CRF at Cambridge biomedical campus *or* Windsor Research Unit at Fulbourn hospital *or* a suitable local facility such as GP surgery, mental health team base *or* at their home according to participant's preference.

The visits will be conducted by a member of the study team and each will last approximately one hour, except the assessment around day 14 which will be more comprehensive and will last for approximately two hours. There be will be regular breaks in order to reduce burden on participants. Refreshments will be provided to participants during their visits.

Procedure:

1. Schedule for this assessment will be explained to participants.
2. Blood samples: will be taken by a trained staff (see Table 1).
3. Interview for recording side effects and other concomitant treatment
4. Psychiatric measures (questionnaires):
    a. Somatic symptoms and depression severity assessed by BDI-II
    b. Anhedonia assessed by Snaith-Hamilton Pleasure Scale Questionnaire
    c. State-Trait Anxiety Inventory (STAI)
    d. Fatigue assessed by Multi-dimensional Fatigue Inventory
    e. Visual Analogue Scales for Subjective Wellbeing (VAS-W)
    f. European Quality of Life-5 Dimensions (EQ-5D)

    Only on day 14 (follow-up 2):

Self-Compassion Scale – Short Form (SCS-SF)

5. Cognitive tests: *only on day 14 (follow-up 2)*
    a. Psychomotor speed:
        i. CANTAB Reaction Time (RTI) test or a similar test (computerised)
        ii. Digit Symbol Substitution Test (pen and paper) or a similar test
    b. Attention: CANTAB Rapid Visual Information Processing (RVP) test or a similar test (computerised)
    c. Memory: CANTAB Paired Associates Learning (PAL) test or a similar test (computerised)
    d. Executive Function: CANTAB One Touch Stockings of Cambridge (OTS) test or a similar test (computerised)
    e. Emotional Processing:
        i. CANTAB Emotional Bias Task (EBT) or a similar test (computerised)
        ii. Emotional categorization and recall task or a similar task (computerised)

4.8 Final Safety Check and Exit from Study (Day 42)

Approximately 42 days after infusion, a member of the study team will contact participants to provide final debrief and answer any questions they may have.

5.0 Safety Considerations and Monitoring of Adverse Reactions for Infusion

Before Infusion:

Participants will be selected for the intervention cohort based on strict inclusion and exclusion criteria (see above). An interview will be conducted as part of eligibility assessment and on the day of intervention prior to infusion to ensure that participants are safe to take part in the study. In addition, we will carry out blood/other tests to exclude TB, HIV, VZV, Hepatitis B and C because, though unlikely after a single dose, tocilizumab could make these infections worse. Prior to infusion, female participants of child bearing age will be given a urine pregnancy test; we will proceed with infusion only if the test is negative. Participants who are sexually active will be asked to use contraception for six weeks after infusion. Male participants will be also asked not to donate sperm samples for six weeks after infusion.

During Infusion:

Infusions will be given under supervision of a designated study doctor. Participants will be monitored by CRF staff and/or a member of study team throughout the duration of infusion for possible side effects, which will be managed in line with use of tocilizumab for treating patients with rheumatoid arthritis (see below).

After Infusion:

Participants will remain in CRF under observation for a one-hour period after infusion as it is standard for monoclonal antibody infusions. Participants will be advised to seek help if they feel unwell after leaving the hospital through GP or A&E depending on the nature of problem. Before leaving CRF, participants will be given an information sheet that will contain a telephone number their health professionals can call; if necessary we will un-blind the participant and inform their health professional whether the participant had received tocilizumab or normal saline. Adverse reactions will be recorded at follow-up visits. In addition to follow-ups around day 7, 14, and 28 after infusion, we will do a final contact with the patient over phone around day 42 after infusion to check they are well. The expected time for complete elimination of tocilizumab is approximately 42 days after a single infusion[27].

Possible Adverse Reactions of Tocilizumab and Management:

Most patients do not have side effects, but the potential side effects include:

1. **Infusion Reactions:** This is very rare but the patient will be monitored for this (pulse rate and blood pressure will be taken before infusion and at any sign of side effect). If there are any concerns a doctor will review the patient and the infusion will be slowed down or stopped. Participants will be encouraged to report adverse feelings.
2. **Skin Rash:** Itchy skin rashes have occurred in a few people usually around 24 - 48 hours after the second or third infusion of tocilizumab, which can last for several days. Though it is unlikely to occur after a single dose (as used in this study), anti-histamine can be used to reduce this.
3. **Increased Risk of Infection:** Regular treatment with tocilizumab can increase risk of infections, but it is less likely after a single infusion. Participants will be advised to seek help if they develop an infection using standard route, e.g. GP.
4. **Other Side Effects:** can include conjunctivitis, abdominal pain and more non-specific symptoms such as headaches. Temporary changes to blood results including white blood cell count (WCC) and liver enzymes can also occur though unlikely to be

clinically significant after a single infusion. These side effects are generally mild and reversible. We will assess WCC and liver function as part of safety monitoring during follow-up.

Allergic Reaction:
Serious allergic reactions (anaphylactic reactions) such as shortness of breath, swelling of lips can occur during or after infusion, but these are rare. Occurrence of such severe reactions leading to discontinuation of treatment has been reported in 0.1% (3/2644) in the 6-month, controlled trials and in 0.2% (9/4009) in the all-exposure population. These reactions were generally observed during the second to fourth infusion of tocilizumab, so are unlikely to occur after a single dose (as used in this study). We will exclude participants who are allergic to tocilizumab or have a history of severe allergic reaction after any infusion in past. If any of the following signs of allergic reaction occurs, the infusion will be stopped. A doctor will be informed who will examine the patient. Anaphylactic reactions will be managed according to standard clinical practice, including taking the patient to A&E if necessary.

- Swelling of the lips
- Hives (red, raised, itchy patches of skin)
- Difficulty breathing
- Chest pain
- The presence of high or low blood pressure (change by more than 20mmHg).
- Symptoms of light headedness, dizziness or headache may alert to the presence of a marked change in blood pressure

6.0 Materials and Assessments

6.1 Validated Clinical Scales & Questionnaires

The Patient Health Questionnaire (PHQ-9)
The Patient Health Questionnaire (PHQ-9, Appendix 1) is a brief and useful instrument used in clinical practice and research for screening, diagnosing, monitoring and measuring the severity of depression. PHQ-9 is a reliable and valid measure of depression severity, making possible a criteria-based diagnosis of depression[33]. The PHQ-9 comprises of 10 items asking participants to rate the frequency with which they experienced each of the items over the past two weeks: Not at all (=0), Several days (=1), More than half the days (=2) and Nearly every day (=3). The PHQ-9 total score ranges from 0 to 27, with total score ≥ 5 for mild depression;

≥ 10 for moderate depression; ≥ 15 for moderately severe depression and ≥ 20 for severe depression. Completing this self-report measure will take approximately 5 minutes.

Clinical Interview Schedule Revised (CIS-R):

The CIS-R is a widely used, standardized tool for measuring common mental health disorders in large community samples[36]. In the UK, CIS-R has been used in National Psychiatric Morbidity Survey, a household survey on 10,000 individuals representative of the UK population, in 1993 and 2007[34,35] . The CIS-R is a fully structured assessment, suitable for trained social survey interviewers and does not require any expert knowledge on the part of the interviewers. As such, it can also be administered using personal computers in which the subjects self-complete the questionnaire[36]. The CIS-R elicits responses to 14 areas of symptoms including fatigue, appetite, sleep problem, concentration, irritability, depression, depressive ideas, anxiety, worry, panic, phobia, compulsive behaviours, obsessive thoughts and somatic symptoms. It can be used to generate diagnostic categories according to the International Statistical Classification of Diseases and Related Health Problems (ICD-10), including diagnosis of depression. The computerised version of CIS-R will be used which provides ICD-10 diagnosis of depression automatically.

Beck Depression Inventory (BDI-II)

Beck Depression Inventory (BDI-II, Appendix 2) is a validated questionnaire used to measure symptoms of depression[37]. It consists of 21 items covering somatic and psychological symptoms of depression, and can take about 10 minutes to complete. The primary outcome for this study will be total somatic symptom score constructed by summing scores for seven relevant BDI-II items (4=lack of pleasure, 15=loss of energy, 16=changes in sleeping pattern, 18=changes in appetite, 19=concentration difficulty, 20=tiredness or fatigue, and 21=loss of interest in sex).

Snaith-Hamilton Pleasure Scale (SHAPS)

Anhedonia, the inability to experience pleasure, is a key feature of depression. The Snaith-Hamilton Pleasure Scale[38] (SHAPS, Appendix 3) is a short, 14-item questionnaire to measure anhedonia, which has been shown to be valid and reliable in healthy and clinical samples[39]. Each of the 14 items has a set of four response categories: Definitely Agree (=1), Agree (=2), Disagree (=3), and Definitely Disagree (=4). A higher total score indicates higher levels of state anhedonia. Completing the scale will take approximately 5 minutes.

State-Trait Anxiety Inventory (STAI)

The State-Trait Anxiety Inventory (STAI, Appendix 4) is a self-report measure of anxiety affect, including two dimensions: state anxiety and trait anxiety[40]. The state anxiety dimension measures participants' self-reported anxiety at the moment they take the questionnaire, whereas the trait anxiety dimension measures the relatively enduring anxiety personality trait. The STAI will be administered at baseline and follow-up visits. In order to avoid habituation, we will present the questionnaire items in a random order at each visit. Completing the questionnaire will take approximately 5 minutes.

Multi-dimensional Fatigue Inventory (MFI)

The Multi-dimensional Fatigue Inventory (MFI, Appendix 5) is a commonly used, self-report measure that assesses five principal manifestations of fatigue, including: general fatigue (GF), physical fatigue (PF), reduced activity (RA), mental fatigue (MF) and reduced motivation (RM)[41]. The questionnaire comprises of 20 items, with each of the five dimension including 4 items. Participants rate each statement on a 5-point scale according to how accurately it applies to them in the last 7 days. The rating for each statement ranges from 1 = "yes, that is true" to 5 = "no, that is not true". The score from each statement is summed to produces a total. A higher total score indicates a higher degree of fatigue. Completing the inventory will take approximately 10 minutes.

Visual Analogue Scale for Subjective Wellbeing (VAS-W) to Assess Side Effects

The Visual Analogue Scale for Subjective Wellbeing[42] (VAS-W, Appendix 6) comprises of 16 visual analogue scales measuring different aspects of participants' mood. Each visual analogue scale ranges between two opposite states (e.g. "alert" vs "drowsy"; "calm" vs "excited") and participants are asked to indicate how they currently feel regarding the states described on each scale. Overall, the VAS-W comprises of scales that can be categorised into four wellbeing dimensions: 1. "mental sedation or intellectual impairment" (e.g. "mentally slow" – "quick-witted"); 2. "physical sedation or bodily impairment" (e.g. "strong" – "clumsy"); 3. "tranquilization or calming effects" (e.g. "tense" – "relaxed") and 4. "other types of feelings" (e.g. "happy" – "sad")[42]. Completing the VAS-W will take no longer than 5 minutes.

Perceived Stress Scale (PSS)

The Perceived Stress Scale[43] (PSS, Appendix 7) is a widely used psychological measure for assessing the extent to which participants appraise situations in their life as stressful. The 10-item self-reporting instrument measures the perception of stress by asking participants to rate statements relating to their feelings and thoughts during the last month. Participants respond to each statement according to how often they felt in that particular way. The scale ranges from “never” = 0 to “very often” = 4 and the total score is obtained from reversing responses from the four positively stated items (i.e. 0 = 4, 1 = 3, 2 = 2, 3 = 1, 4 = 0) and summing across all scale items. Completing the scale will take 3-5 minutes.

Self-Compassion Scale – Short Form (SCS-SF)

The Self-Compassion Scale – Short Form (SCS-SF, Appendix 8) is a 12-item questionnaire used to measure self-compassion[44]. It includes three subdomains: self-judgement, isolation and over-identification. The scale is titled “How I typically act towards myself in difficult times”. Participants rate each statement on a scale of 1 to 5, where 1 = “almost never”, 5 = “always”. Completing the questionnaire will take 3-5 minutes.

European Quality of Life-5 Dimensions (EQ-5D)

European Quality of Life-5 Dimensions (EQ-5D, Appendix 9) is a standardised 5 item instrument used as a measure of health-related quality of life[45]. It is a self-report measure prompting participants to assess their mobility, self-care, usual activities, pain/discomfort and anxiety/depression. Completing the questionnaire will take 2 minutes.

6.2 Other Questionnaires

The following bespoke questionnaires will be used to record eligibility, sociodemographic, lifestyle from participants.

Postal Screening Questionnaire

The Postal Screening Questionnaire will be used to record the medical history of potentially suitable participants. This questionnaire will be complete by participants as one of the first screening methods for eligibility for the study. The Postal Screening Questionnaire (Appendix 10) will take approximately 5 minutes to complete.

Medical History Questionnaire

Participants' medical history will be recorded during the face-to-face eligibility assessment visit using the Medical History Questionnaire (Appendix 11). This questionnaire allows for an easy identification of any conditions past or current. The medical information self-reported by participants will be verified with their General Practitioner. Completing this questionnaire will last approximately 10 minutes.

Antidepressant History Questionnaire

The Antidepressant History Questionnaire (Appendix 12) will be used to record all anti-depressant treatment received by the participants, including antidepressant medication currently taken and the degree to which participants have experienced an improvement after each antidepressant treatment. Current anti-depressant treatment will be verified with GP. Completing this questionnaire will last approximately 10 minutes.

Physical Measurements Form

Physical measurements, including weight, height, systolic and diastolic blood pressure, and pulse will be recorded at the baseline data collection visit. Before each blood sampling during the study, systolic and diastolic blood pressure and pulse will be recorded using the Physical Measurements Form (Appendix 13). Recording this information will take approximately 10 minutes.

Sociodemographic Questionnaire

The Sociodemographic Questionnaire (Appendix 14) comprises of 5 questions relating to participants' sociodemographic information. Completing this questionnaire will take approximately 5 minutes.

Drug and Alcohol Questionnaire

The Drug and Alcohol Questionnaire (Appendix 15) will record data on participants' use of any drugs, including alcohol and tobacco. Participants will report the frequency of use for each drug, the age when they first tried it and whether they are past or current users. Completing this questionnaire will take approximately 5 minutes.

6.3 Validated Cognitive Assessments

National Adult Reading Test (NART)

The National Adult Reading Test[46] (NART, Appendix 16) is widely used in research as a measure of premorbid IQ[47]. The NART assesses participant's vocabulary by presenting them with a list of 50 words with irregular spellings in British English (e.g. "aisle") and asking them to provide the pronunciation of the words. NART scores are converted to predict IQ scores on the Wechsler Adult Intelligence Scale[48]. Approximate completion time 10 minutes.

Digit Symbol Substitution Test (DSST)

The Digit Symbol Substitution Test (DSST, Appendix 17) is a neuropsychological test sensitive to depression. The Digit Symbol Substitution Test is part of the Wechsler Adult Intelligence Scale and measures psychomotor processing speed. During this test, participants are presented with digit-symbol pairs followed by a list of digits and a corresponding blank box. For the duration of 120 seconds, participants are instructed to fill in as many boxes with the matching symbol for each number. The original or a similar test will be used.

In addition, we will use the following validated tasks from the Cambridge Neuropsychological Test Automated Battery (CANTAB) – *or* other tasks that are similar to these – to test cognitive function.

Reaction Time (RTI)

The Reaction Time (RTI) test assesses psychomotor ability, measuring simple and five-choice movement time, reaction time, response accuracy and impulsivity. During the test, participants are required to respond as soon as a yellow dot appears on the screen by using a press pad, by touching the screen or both. Reaction time is recorded as the speed with which participants release the press pad in response to the presentation of the stimulus, whereas motor time is recorded as the time taken to touch the stimulus on the screen after the press pad has been released. Test duration is 5 minutes.

Rapid Visual Information Processing (RVP)

The Rapid Visual Information Processing (RVP) test assesses sustained attention, measuring response accuracy, target sensitivity, and reaction time. Participants are presented with a white box in the middle of the computer screen, presenting in a pseudo-random order digit

from 2 to 9. Participants are required to detect and report by pressing a press pad target sequences of digits (e.g. 2-4-6, 3-5-7, 4-6-8). Completing the test takes 10 minutes.

Paired Associates Learning (PAL)

The Paired Associates Learning (PAL) test assesses episodic memory and new learning using pattern recognition. Participants are presented on the screen with a number of boxes of which some contain a pattern. The patterns are then displayed on the screen and the participants are required to locate the correct box where the pattern originated from. The difficulty level increases through the test and the duration of the task is 10 minutes. The test offers twenty-one outcome measures, covering the number of trials required to answer correctly, memory scores and stages completed in the allocated time.

One Touch Stockings of Cambridge (OTS)

One Touch Stockings of Cambridge (OTS) is a test of executive function, assessing spatial planning and working memory. Participants are shown two displays containing three coloured balls presented in a 3-D format. There is a row of numbered boxes along the bottom of the screen. Participants will be shown how to move the balls in order to recreate the pattern shown in the upper display. Participants need to work out in their head how many moves the solutions require and then select the appropriate box at the bottom of the screen to indicate their response. Completing the OTS will take 10 minutes.

Emotional Bias Task (EBT)

The Emotional Bias Task (EBT) assesses perceptual bias in facial emotion recognition. Participants view faces that are morphed between happy and sad emotions of varied intensities. Participants are instructed to indicate which emotion the face displayed from the two options. A ‘balance point’ indicates when a participant is equally likely to perceive happy or sad emotions. This measure indicates whether there is a bias in emotion perception, and the extent of the bias. Completing the EBT will take 4 minutes.

Emotional Categorization and Recall Task

We will use an emotional categorization task developed by Harmer and colleagues at Oxford based on Anderson's list of personality-trait words[49]. Sixty personality characteristics selected to be disagreeable (e.g., domineering, untidy, and hostile) or agreeable (e.g., cheerful, honest, and optimistic) will be presented on the computer screen for 500msec each. These words are

matched in terms of word length, ratings of usage frequency, and meaningfulness. Volunteers will be asked to categorize these personality traits as likable or dislikable as quickly and as accurately as possible. Fifteen minutes after completion of the emotional categorization task, participants will be asked to recall as many of the personality traits as possible.

6.4 Venous Blood Sampling for Peripheral Immuno-phenotypes

Venous blood sampling will be done on the following occasions to establish eligibility, assess and monitor safety, and to assay outcome measures (see Table 1). Repeat or unscheduled samples may be taken for safety reasons or technical issues. Blood samples would be transported directly to lab on the day of collection where they will be stored and analysed as per laboratory protocol.

Table 1: Schedule of Venous Blood Sampling

**Purpose**	**Test**	**Timing**				
		**Eligibility**	**Baseline**	**Day 7 f/u**	**Day 14 f/u**	**Day 28 f/u**
Screening	hsCRP	5ml				
Eligibility/ Safety	LFT, lipid profile, renal function, HCG, antinuclear antibody, HIV, Hep B, Hep C, VZV IgG, FBC, and Tuberculosis Quatiferon Gold (intervention cohort only)		15ml		7.5ml	
Outcomes	Inflammatory markers, cardio-metabolic markers, IDO activation, WCC, immune-phenotyping, and gene expression analysis		35ml	25ml	35ml	25ml
**Total***		**5ml**	**50ml**	**25ml**	**42.5ml**	**25ml**

* All amounts are approximate

6.5 Genotyping

A pharmacogenomic blood sample (5ml) will be collected from all subjects to allow for pharmacogenomic research at baseline data collection. Participation in the pharmacogenomic research is mandatory. It is recognized that genetic variation can be an important contributory factor to inter-individual differences in disease susceptibility and prognosis but also biomarker profiles.

7.0 Participant Payment

Participants will be reimbursed for their time and inconvenience as follows: £100 for the baseline data collection meeting, £50 for the infusion session, £75 for follow-up 1, £100 for follow-up 2, and £75 for follow-up 3. These sessions include blood sampling, questionnaires, physical assessments, psychiatric measures, and cognitive testing. Participants will be reimbursed for their travel expenses for each visit. We will arrange and pay for train tickets, if required.

8.0 Informed Consent and Ethical Approval:

Informed consent will be obtained which will include consent to randomise to tocilizumab or normal saline, to contact GP to inform about participation in study, to verify medical history with GP to establish eligibility, and to inform GP any results/outcomes if necessary. Consent for additional tests to establish safety for tocilizumab infusion will be also obtained. Ethical approval for the study will be obtained.

9.0 Data Management and Statistical Analysis

9.1 Data Management

All potential participants will be assigned a unique study specific participant ID number. All data including personal details identifying individuals and anonymised data will be subject to good practice as laid down in the Data Protection Act. Each stage of inviting, informing and assessing a particular participant is tracked so that their (anonymised) current status within the study is known and assessment and other appointment dates are forecasted. This information is held on the secure, password protected Tracking Database. Anonymised data from assessments will be uploaded to the secure, password protected Assessment Database using web-based data entry systems, transcribing from paper copy as necessary. Minimal personal data (age, sex) will be indexed by each participant's unique ID number.

9.2 Statistical Analysis

For randomized participants (intervention cohort), an intention-to-treat approach will be taken for data analysis. We will compare outcome measures between treatment and placebo groups controlling for baseline scores. We predict that IL-6 inhibition will attenuate depressive symptoms, proinflammatory cytokine concentration, IDO activation, and proinflammatory gene expression in PBMC. This mechanistic experiment will focus on overall pattern of results rather than *P*-values for individual tests of statistical significance.

The secondary, observational analysis will compare psychiatric, cognitive, blood and other biomarkers between inflamed and non-inflamed groups using appropriate statistical tests.

10.0 Risks

Depression-related Risks:

All of participants with depression will be under the care of their general practitioners or psychiatric teams and the procedures will not involve any treatment modifications or significant delays in receiving treatment.

Participants recruited from the community not currently in treatment for depression will be carefully assessed and, with their consent, this information will be shared with their general practitioner. Individuals who do not consent to share clinical information with their primary and secondary healthcare services will not be eligible for participation.

Psychiatric interviews can reveal information, which can be distressing for the participants. We will *not* explore history of childhood abuse or other traumatic life events. If a participant becomes distressed during an interview, or does not wish to continue for any reason, the researcher will immediately stop the interview. If there is any concern for the participant's safety, the researcher will discuss this with chief investigator who will liaise participants GP and/or psychiatrist appropriately.

Participants will be assessed for suicidality during eligibility assessment. Those with current suicidal thoughts (assessed by BDI-II item 9=suicidal thoughts or wishes score 3) or history of suicide attempt, deliberate self-harm, overdose within six months prior to eligibility assessment will be excluded.

Procedure-related Risks:

Venepuncture:

This study requires blood draw on different occasions during the study. Blood taking is associated with mild discomfort though other side effects are rare. Efforts will be made to minimise discomfort. Blood taking will be performed by a nurse, doctor or research team member trained in venepuncture.

Chest X-ray:

As part of the study, participants in the intervention cohort will receive a PA chest x-ray to screen for Tuberculosis. This x-ray is additional to any standard clinical care outside of the trial. The typical effective dose for a PA chest x-ray is estimated as 0.016 mSv, based on a National DRL of 0.1 Gycm2 and a conversion coefficient of 0.16 mSv/Gycm2 from HPA-CRCE-028. For adults in the general population, the dose of 0.016 mSv is estimated to correspond to a cancer risk of around 1 in 1.5 million. This dose is equivalent to that received, on average in the UK, from natural sources of radiation in the environment every three to ten days. All examinations will need to be completed in compliance with local IR(ME)R Employer's Procedures.

Inflammation Status:

Participants will be informed of their serum/plasma hsCRP level. The proposed threshold for defining participants as 'inflamed' used in this study is serum CRP level ≥3mg/L. Having serum/plasma hsCRP level above this threshold is not necessarily a cause for concern. In the general population, serum CRP levels are 3.0-9.9mg/L in about 30% people, and levels >10mg/L are seen in about 10-15% of people according to a large scale study from the United States[50]. Reasons for elevated CRP in the absence of an acute infection or chronic inflammatory illness could include obesity, smoking, alcohol use, lack of exercise, so knowledge of 'inflammation status' might prompt participants to adopt a healthier lifestyle. If serum CRP level is very high (>20mg/L) without any apparent explanation such as infection or chronic inflammatory illness, we will inform the GP and the participant will be excluded from the study.

Infusion of Tocilizumab or Normal Saline:

Delineated fully in section 5.0 Safety Considerations and Monitoring of Adverse Reactions for Infusion (see above).

Risks to research staff:

If home visits or lone working are required, staff will follow local safety procedures. Home visits will be conducted in pairs whenever possible for first visits.

11.0 Regulatory Ethics Compliance

11.1 Investigator Responsibilities

The investigator is responsible for ensuring that the study is performed in accordance with the protocol, current ICH guidelines on Good Clinical Practice (GCP), and applicable regulatory requirements.

Good Clinical Practice is an international ethical and scientific quality standard for designing, conducting, recording, and reporting studies that involve the participation of human subjects. Compliance with this standard provides public assurance that the rights, safety, and well-being of study subjects are protected, consistent with the principles that originated in the Declaration of Helsinki, and that the study data are credible.

11.2 Independent Ethics Committee or Institutional Review Board

Before the start of the study, the investigator (or sponsor where required) will provide the IEC/IRB with current and complete copies of the following documents (as required by local regulations):

- Final protocol and, if applicable, amendments
- Informed Consent Form or ICF (and any other written materials to be provided to the subjects)
- Subject recruiting materials
- Information on compensation for study-related injuries or payment to subjects for participation in the study, if applicable
- Investigator's curriculum vitae or equivalent information (unless not required, as documented by the IEC/IRB)
- Information regarding funding, name of the sponsor, institutional affiliations, other potential conflicts of interest, and incentives for subjects
- Any other documents that the IEC/IRB requests to fulfil its obligation

This study will be undertaken only after the IEC/IRB has given full approval of the final protocol, amendments (if any, excluding the ones that are purely administrative, with no consequences for subjects, data or study conduct), the ICF, applicable recruiting materials, and subject compensation programs, and the sponsor has received a copy of this approval.

This approval letter must be dated and must clearly identify the IEC/IRB and the documents being approved.

During the study the investigator will send the following documents and updates to the IEC/IRB for their review and approval, where appropriate:

- Protocol amendments (excluding the ones that are purely administrative, with no consequences for subjects, data or study conduct)
- Revision(s) to ICF and any other written materials to be provided to subjects
- If applicable, new or revised subject recruiting materials approved by the sponsor
- Revisions to compensation for study-related injuries or payment to subjects for participation in the study, if applicable
- Summaries of the status of the study at intervals stipulated in guidelines of the IEC/IRB (at least annually)
- New information that may adversely affect the safety of the subjects or the conduct of the study
- Deviations from or changes to the protocol to eliminate immediate hazards to the subjects
- Report of deaths of subjects under the investigator's care
- Notification if a new investigator is responsible for the study at the site
- Any other requirements of the IEC/IRB

For all protocol amendments (excluding the ones that are purely administrative, with no consequences for subjects, data or study conduct), the amendment and applicable ICF revisions must be submitted promptly to the IEC/IRB for review and approval before implementation of the change(s).

At least once a year, the IEC/IRB will be asked to review and reapprove this study. The re-approval should be documented in writing.

At the end of the study, the investigator (or sponsor where required) will notify the IEC/IRB about the study.

11.3 Informed Consent

The general strategy for consent is that all participants will be asked to consent to clinical assessment, venous blood sampling and administration of a single dose of tocilizumab or placebo. Each subject must give written consent according to local requirements after the nature of the study has been fully explained. The ICF(s) must be signed before performance of any study-related activity. The ICF(s) that is/are used must be approved by the reviewing IEC/IRB and be in a language that the subject can read and understand. The informed consent should be in accordance with principles that originated in the Declaration of Helsinki, current ICH and GCP guidelines, applicable regulatory requirements.

Before enrolment in the study, the investigator or an authorized member of the study-site personnel must explain to potential subjects the aims, methods, reasonably anticipated benefits, and potential hazards of the study, and any discomfort participation in the study may entail. Subjects will be informed that their participation is voluntary and that they may withdraw consent to participate at any time. They will be informed that choosing not to participate will not affect the care the subject will receive. Finally, they will be told that the investigator will maintain a subject identification register for the purposes of long-term follow-up if needed and that their records may be accessed by health authorities without violating the confidentiality of the subject, to the extent permitted by the applicable law(s) or regulations. By signing the ICF the subject is authorizing such access and agrees to allow his or her study physician to re-contact the subject for the purpose of obtaining consent for additional safety evaluations, if needed.

The subject will be given sufficient time to read the ICF and the opportunity to ask questions. After this explanation and before entry into the study, consent should be appropriately recorded by means of the subject's personally dated signature. After having obtained the consent, a copy of the ICF must be given to the subject.

11.4 Privacy of Personal Data

The collection and processing of personal data from subjects enrolled in this study will be limited to those data that are necessary to fulfil the objectives of the study. These data must be collected and processed with adequate precautions to ensure confidentiality and compliance with applicable data privacy protection laws and regulations. Appropriate technical and organizational measures to protect the personal data against unauthorized

disclosures or access, accidental or unlawful destruction, or accidental loss or alteration must be put in place.

The informed consent obtained from the subject includes explicit consent for the processing of personal data and for the investigator/institution to allow direct access to his or her original medical records (source data/documents) for study-related monitoring, audit, IEC/IRB review, and regulatory inspection.

The subject has the right to request through the investigator access to his or her personal data and the right to request rectification of any data that are not correct or complete. Reasonable steps will be taken to respond to such a request, taking into consideration the nature of the request, the conditions of the study, and the applicable laws and regulations.

11.5 Long-Term Retention of Samples for Additional Future Research

Samples collected in this study may be stored for up to 10 years after the completion of the study for additional research. Samples will only be used to understand the role of inflammation in depression, and to develop tests/assays/drugs related to inflammation and/or depression. The research may begin at any time during the study or the post-study storage period.

Stored samples will be coded throughout the sample storage and analysis process and will not be labelled with personal identifiers. Subjects may withdraw their consent for their samples to be stored for research.

11.6 Country Selection

This study will only be conducted in the United Kingdom.

12.0 References

1. Harrison NA, Brydon L, Walker C, Gray MA, Steptoe A, Critchley HD. Inflammation causes mood changes through alterations in subgenual cingulate activity and mesolimbic connectivity. Biological psychiatry 2009;66:407-14.
2. Dantzer R, O'Connor JC, Freund GG, Johnson RW, Kelley KW. From inflammation to sickness and depression: when the immune system subjugates the brain. Nature reviews Neuroscience 2008;9:46-56.
3. Howren MB, Lamkin DM, Suls J. Associations of depression with C-reactive protein, IL-1, and IL-6: a meta-analysis. Psychosomatic medicine 2009;71:171-86.
4. Haapakoski R, Mathieu J, Ebmeier KP, Alenius H, Kivimaki M. Cumulative meta-analysis of interleukins 6 and 1beta, tumour necrosis factor alpha and C-reactive protein in patients with major depressive disorder. Brain, behavior, and immunity 2015;49:206-15.
5. Goldsmith DR, Rapaport MH, Miller BJ. A meta-analysis of blood cytokine network alterations in psychiatric patients: comparisons between schizophrenia, bipolar disorder and depression. Molecular psychiatry 2016.
6. Maes M, Bosmans E, De Jongh R, Kenis G, Vandoolaeghe E, Neels H. Increased serum IL-6 and IL-1 receptor antagonist concentrations in major depression and treatment resistant depression. Cytokine 1997;9:853-8.
7. O'Brien SM, Scully P, Fitzgerald P, Scott LV, Dinan TG. Plasma cytokine profiles in depressed patients who fail to respond to selective serotonin reuptake inhibitor therapy. Journal of psychiatric research 2007;41:326-31.
8. Khandaker GM, Pearson RM, Zammit S, Lewis G, Jones PB. Association of serum interleukin 6 and C-reactive protein in childhood with depression and psychosis in young adult life: a population-based longitudinal study. JAMA psychiatry 2014;71:1121-8.
9. Khandaker GM, Stochl J, Zammit S, Goodyer I, Lewis G, Jones PB. Childhood inflammatory markers and intelligence as predictors of subsequent persistent depressive symptoms: a longitudinal cohort study. Psychological medicine 2017:1-12.
10. Zalli A, Jovanova O, Hoogendijk WJ, Tiemeier H, Carvalho LA. Low-grade inflammation predicts persistence of depressive symptoms. Psychopharmacology 2016;233:1669-78.
11. Gimeno D, Kivimaki M, Brunner EJ, et al. Associations of C-reactive protein and interleukin-6 with cognitive symptoms of depression: 12-year follow-up of the Whitehall II study. Psychological medicine 2009;39:413-23.
12. Davey Smith G, Ebrahim S. ‘Mendelian randomization’: can genetic epidemiology contribute to understanding environmental determinants of disease? International journal of epidemiology 2003;32:1-22.
13. Khandaker GM, Zammit S, Burgess S, Lewis G, Jones PB. Association between a functional interleukin 6 receptor genetic variant and risk of depression and psychosis in a population-based birth cohort. Brain, behavior, and immunity 2017.
14. O'Connor JC, Lawson MA, Andre C, et al. Lipopolysaccharide-induced depressive-like behavior is mediated by indoleamine 2,3-dioxygenase activation in mice. Molecular psychiatry 2009;14:511-22.
15. Salazar A, Gonzalez-Rivera BL, Redus L, Parrott JM, O'Connor JC. Indoleamine 2,3-dioxygenase mediates anhedonia and anxiety-like behaviors caused by peripheral lipopolysaccharide immune challenge. Hormones and behavior 2012;62:202-9.
16. Hodes GE, Pfau ML, Leboeuf M, et al. Individual differences in the peripheral immune system promote resilience versus susceptibility to social stress. Proceedings of the National Academy of Sciences of the United States of America 2014;111:16136-41.
17. Kappelmann N, Lewis G, Dantzer R, Jones PB, Khandaker GM. Antidepressant activity of anti-cytokine treatment: a systematic review and meta-analysis of clinical trials of chronic inflammatory conditions. Molecular psychiatry 2018;23:335-43.

18. Capuron L, Fornwalt FB, Knight BT, Harvey PD, Ninan PT, Miller AH. Does cytokine-induced depression differ from idiopathic major depression in medically healthy individuals? Journal of affective disorders 2009;119:181-5.
19. Capuron L, Gumnick JF, Musselman DL, et al. Neurobehavioral effects of interferon-alpha in cancer patients: phenomenology and paroxetine responsiveness of symptom dimensions. Neuropsychopharmacology : official publication of the American College of Neuropsychopharmacology 2002;26:643-52.
20. Jokela M, Virtanen M, Batty GD, Kivimaki M. Inflammation and Specific Symptoms of Depression. JAMA psychiatry 2016;73:87-8.
21. Ferrie JE, Kivimaki M, Akbaraly TN, et al. Associations between change in sleep duration and inflammation: findings on C-reactive protein and interleukin 6 in the Whitehall II Study. American journal of epidemiology 2013;178:956-61.
22. Skapinakis P, Lewis G, Mavreas V. Temporal relations between unexplained fatigue and depression: longitudinal data from an international study in primary care. Psychosomatic medicine 2004;66:330-5.
23. Harrison NA, Voon V, Cercignani M, Cooper EA, Pessiglione M, Critchley HD. A Neurocomputational Account of How Inflammation Enhances Sensitivity to Punishments Versus Rewards. Biological psychiatry 2016;80:73-81.
24. Wium-Andersen MK, Orsted DD, Nielsen SF, Nordestgaard BG. Elevated C-reactive protein levels, psychological distress, and depression in 73, 131 individuals. JAMA psychiatry 2013;70:176-84.
25. Raison CL, Rutherford RE, Woolwine BJ, et al. A randomized controlled trial of the tumor necrosis factor antagonist infliximab for treatment-resistant depression: the role of baseline inflammatory biomarkers. JAMA psychiatry 2013;70:31-41.
26. Choy EH, Isenberg DA, Garrood T, et al. Therapeutic benefit of blocking interleukin-6 activity with an anti-interleukin-6 receptor monoclonal antibody in rheumatoid arthritis: a randomized, double-blind, placebo-controlled, dose-escalation trial. Arthritis and rheumatism 2002;46:3143-50.
27. Woo P, Wilkinson N, Prieur AM, et al. Open label phase II trial of single, ascending doses of MRA in Caucasian children with severe systemic juvenile idiopathic arthritis: proof of principle of the efficacy of IL-6 receptor blockade in this type of arthritis and demonstration of prolonged clinical improvement. Arthritis research & therapy 2005;7:R1281-8.
28. Calabrese LH, Rose-John S. IL-6 biology: implications for clinical targeting in rheumatic disease. Nature reviews Rheumatology 2014;10:720-7.
29. Bonaccorso S, Marino V, Biondi M, Grimaldi F, Ippoliti F, Maes M. Depression induced by treatment with interferon-alpha in patients affected by hepatitis C virus. Journal of affective disorders 2002;72:237-41.
30. Khandaker GM, Dantzer R. Is there a role for immune-to-brain communication in schizophrenia? Psychopharmacology 2016;233:1559-73.
31. Raison CL, Capuron L, Miller AH. Cytokines sing the blues: inflammation and the pathogenesis of depression. Trends Immunol 2006;27:24-31.
32. Wiles N, Thomas L, Abel A, et al. Clinical effectiveness and cost-effectiveness of cognitive behavioural therapy as an adjunct to pharmacotherapy for treatment-resistant depression in primary care: the CoBalT randomised controlled trial. Health technology assessment 2014;18:1-167, vii-viii.
33. Kroencke K, Spitzer R, Williams J. The PHQ-9: validity of a brief depression severity measure [Electronic version]. Journal of General Internal Medicine 2001;16:606-13.
34. Jenkins R, Lewis G, Bebbington P, et al. The National Psychiatric Morbidity surveys of Great Britain–initial findings from the household survey. Psychological medicine 1997;27:775-89.
35. Spiers N, Bebbington P, McManus S, Brugha TS, Jenkins R, Meltzer H. Age and birth cohort differences in the prevalence of common mental disorder in England: National Psychiatric Morbidity Surveys 1993–2007. The British Journal of Psychiatry 2011;198:479-84.

36. Lewis G. Assessing psychiatric disorder with a human interviewer or a computer. Journal of epidemiology and community health 1994;48:207-10.
37. Beck AT, Steer RA, Brown GK. Manual for the Beck Depression Inventory-II. . San Antonio, TX: Psychological Corporation; 1996.
38. Snaith R, Hamilton M, Morley S, Humayan A, Hargreaves D, Trigwell P. A scale for the assessment of hedonic tone the Snaith-Hamilton Pleasure Scale. The British Journal of Psychiatry 1995;167:99-103.
39. Franken IH, Rassin E, Muris P. The assessment of anhedonia in clinical and non-clinical populations: further validation of the Snaith–Hamilton Pleasure Scale (SHAPS). Journal of affective disorders 2007;99:83-9.
40. Spielberger CD. State-trait Anxiety Inventory: A Comprehensive Bibliography: Consulting Psychologists Press; 1984.
41. Smets E, Garssen B, Bonke Bd, De Haes J. The Multidimensional Fatigue Inventory (MFI) psychometric qualities of an instrument to assess fatigue. Journal of psychosomatic research 1995;39:315-25.
42. Bond A, Lader M. The use of analogue scales in rating subjective feelings. British Journal of Medical Psychology 1974;47:211-8.
43. Cohen S, Kamarck T, Mermelstein R. A global measure of perceived stress. Journal of health and social behavior 1983:385-96.
44. Raes F, Pommier E, Neff KD, Van Gucht D. Construction and factorial validation of a short form of the Self-Compassion Scale. Clinical psychology & psychotherapy 2011;18:250-5.
45. EuroQol - a new facility for the measurement of health-related quality of life. Health Policy 1990;16:199-208.
46. Nelson HE, Willison J. National Adult Reading Test (NART): Nfer-Nelson Windsor; 1991.
47. McGurn B, Starr J, Topfer J, et al. Pronunciation of irregular words is preserved in dementia, validating premorbid IQ estimation. Neurology 2004;62:1184-6.
48. Bright P, Hale E, Gooch VJ, Myhill T, Van Der Linde I. The National Adult Reading Test: restandardisation against the Wechsler Adult Intelligence Scale—Fourth edition. Neuropsychological Rehabilitation 2016:1-9.
49. Anderson NH. Likableness ratings of 555 personality-trait words. Journal of personality and social psychology 1968;9:272-9.
50. Woloshin S, Schwartz LM. Distribution of C-reactive protein values in the United States. The New England journal of medicine 2005;352:1611-3.

13.0 Amendments

No.	Type	Date	Amendment Details
1	Substantial	02/08/18	Addition of new outcome measures (State Trait Anxiety Inventory, Self-Compassion Scale – Short Form, European Quality of Life – 5 Dimension, CANTAB One Touch Stockings of Cambridge, CANTAB Emotional Bias Task), minor revisions to current patient facing documents, and associated protocol updates listing new documents and changes.
2	Substantial	13/03/19	Change to the lifetime diagnosis of eating disorder as an exclusionary criterion for the study. Now defined as "Currently active diagnosed eating disorder likely to compromise ability to take part (determined by Chief Investigator)". A follow-up letter from the GP practice will also now be sent to participants identified via electronic search who have not responded to an initial letter invitation. The protocol and PIS were updated accordingly
3	Non-substantial	02/04/19	Minor updates to the infusion information form and intervention visit form. Added a line "Note: Please use contraception for six weeks since infusion." to remind participants of this. Recording participant opinion regarding what infusion they have received. This remains in final follow-up assessment form.
4	Non-substantial	23/08/19	Non-substantial amendment notification.
5	Substantial	24/09/20	The following changes have been made solely to minimize the risk of COVID-19 infection to study participants and research staff. A) Change in inclusion/exclusion criteria: We will exclude groups deemed at higher risk of COVID-19 complications, e.g., age >55y, BMI >35, BAME, asthma/other lung disease, and type-two diabetes. B) Change in study procedure:1. Data collection for all sessions will be carried out remotely, e.g., telephone, video conference, as much as possible. 2. Face-to-face visits will be limited to only blood collection (eligibility, baseline and follow-up two), chest X-ray at baseline assessment, and infusion visit. No bloods at follow-up one/ three. 3. We have removed cognitive assessments entirely to reduce face-to-face contact.
6	Substantial	21/12/21	Change of sponsor of the study from University of Cambridge / Cambridgeshire and Peterborough NHS Foundation Trust to University of Bristol; 2) Add University of Bristol as a new study site and move data and blood samples collected to Bristol to complete data analysis and publication of findings; 3) Change of indemnity insurance provider from University of Cambridge to University of Bristol in line with change in sponsor; 4)

			Change study protocol and PIS to reflect change of sponsor; 5) Inform participants of change of sponsor and transfer of data and blood samples to Bristol; 6) Change end date to 30.6.2023 to complete data analysis and publication.
7	Non-substantial	23/08/23	Change end date to 30.6.2024 to complete data analysis and publication.
8	Non-substantial	21/02/24	Change end date to 31.12.2025 to complete sample analysis, data analysis and publication.
9	Non-substantial	18/11/25	This amendment extends the study end date from 31/12/2025 to 31/12/2030 to allow completion of planned analyses and future work within the existing protocol, with no changes to methodology or participant involvement. The extension reflects the time needed for a doctoral or post-doctoral fellowship to complete all analyses within the approved scope.
