# Supplementary material for: Interleukin 6 as a Treatment Target for Depression: A Proof-of-Concept Randomized Clinical Trial
Source: JAMA Psychiatry. 2026 May 20;83(8):857–63. doi: 10.1001/jamapsychiatry.2026.1053 (PMC13191455; doi:10.1001/jamapsychiatry.2026.1053)
Supplement: Supplement 2. — eAppendix. eTable 1. Inclusion and exclusion criteria for the Insight Study eTable 2. The Insight Study outcome measures and time of assessment eTable 3. Lower and upper detection limits for inflammatory proteins measured in the Insight Study eTable 4. Sociodemographic, clinical, and cognitive characteristics of the Insight Study participants at baseline eTable 5. Clinical and cognitive outcome scores per timepoint split by sex eTable 6. Immune protein concentrations of Insight Study participants at baseline and post-infusion split by sex eTable 7. Immune protein concentrations of Insight Study participants at baseline and post-infusion eTable 8. Effect of tocilizumab treatment on clinical outcomes in the Insight Study eTable 9. Insight Study participants depression severity category at baseline according to BDI-II score eTable 10. Post-hoc analysis of required sample size estimates for depression outcomes for future RCTs eTable 11. Effect of tocilizumab treatment on cognitive outcomes in the Insight Study eTable 12. Adverse events eFigure 1. Overview of design and procedures for the Insight Study eFigure 2. The CONSORT 202537 flow diagram for the Insight trial eFigure 3. Serum hs-CRP, IL-6, sIL-6R, and sgp130 levels at baseline and post-infusion in tocilizumab and placebo arms in the Insight Study eFigure 4. Effect of tocilizumab treatment on depression remission and response eFigure 5. Effect of tocilizumab treatment on individual depressive symptoms in the Insight Study eFigure 6. Effect of baseline IL-6 and hs-CRP concentrations on treatment response in total fatigue score in the Insight Study eReferences [file jamapsychiatry-e261053-s002.pdf]

Supplemental Online Content

Foley ÉM, Turner N, Margelyte R, et al. Interleukin 6 as a treatment target for depression: a proof-of-concept randomized clinical trial. *JAMA Psychiatry*. Published online May 20, 2026. doi:10.1001/jamapsychiatry.2026.1053

eAppendix.
eTable 1. Inclusion and exclusion criteria for the Insight Study
eTable 2. The Insight Study outcome measures and time of assessment
eTable 3. Lower and upper detection limits for inflammatory proteins measured in the Insight Study
eTable 4. Sociodemographic, clinical, and cognitive characteristics of the Insight Study participants at baseline
eTable 5. Clinical and cognitive outcome scores per timepoint split by sex
eTable 6. Immune protein concentrations of Insight Study participants at baseline and post-infusion split by sex
eTable 7. Immune protein concentrations of Insight Study participants at baseline and post-infusion
eTable 8. Effect of tocilizumab treatment on clinical outcomes in the Insight Study
eTable 9. Insight Study participants depression severity category at baseline according to BDI-II score
eTable 10. Post-hoc analysis of required sample size estimates for depression outcomes for future RCTs
eTable 11. Effect of tocilizumab treatment on cognitive outcomes in the Insight Study
eTable 12. Adverse events
eFigure 1. Overview of design and procedures for the Insight Study
eFigure 2. The CONSORT 2025 flow diagram for the Insight trial
eFigure 3. Serum hs-CRP, IL-6, sIL-6R, and sgp130 levels at baseline and post-infusion in tocilizumab and placebo arms in the Insight Study
eFigure 4. Effect of tocilizumab treatment on depression remission and response
eFigure 5. Effect of tocilizumab treatment on individual depressive symptoms in the Insight Study
eFigure 6. Effect of baseline IL-6 and hs-CRP concentrations on treatment response in total fatigue score in the Insight Study
eReferences

This supplemental material has been provided by the authors to give readers additional information about their work.

© 2026 Foley ÉM et al. *JAMA Psychiatry*.

eAppendix

1. Trial design

At baseline, participants completed questionnaires, cognitive tests, and gave blood samples (see below). Safety checks covered pregnancy, lipids, liver and kidney function, immune-mediated disease, and infections (VZV, TB, HIV, hepatitis B and C).

Sealed Envelope randomly assigned participants to tocilizumab or placebo arms using minimisation to ensure the two groups were comparable on depression severity (Beck's Depression Inventory/BDI-II total score bands 7-11, 12-16, 17-21) and sex (female/male). Randomisation codes were sent to a designated pharmacy for blinded dispensing. Infusions were prepared and administered by personnel at a clinical research facility designated for early-phase trials. Study staff were not involved in dispensing or infusion preparation, and infusion packs were visually indistinguishable, ensuring double-blind (participants and study team) approach.

Participants received one intravenous infusion of either tocilizumab (8mg/kg body weight, maximum 800mg/patient) – equivalent to one-month's treatment – or normal saline administered as slow drip over one hour followed by one hour of observation with vital sign monitoring.

The post-infusion assessment timeline was chosen based on known pharmacokinetics of tocilizumab and how it is used in clinical practice currently for treatment of inflammatory conditions such as rheumatoid arthritis (RA). The half-life of tocilizumab is approximately 11-13 days[1]. Early studies in arthritis patients show that a single tocilizumab infusion improves CRP and other laboratory measures within 48 hours, with most noticeable result in one-to-two weeks[2,3]. For treatment of RA in clinical practice, tocilizumab is given as a single monthly infusion (a loading dose at the start is not required). As a clinical trial, we were required to prespecify a single primary endpoint. Day 14 was chosen as a pragmatic midpoint where drug levels would still be sufficient to ensure anti-inflammatory effect. However, a key objective of this proof-of-concept study was to identify timing of effect, in addition to treatment-sensitive outcomes and effect sizes. Therefore, we chose assessments at 7, 14 and 28 days post-infusion to capture early, peak and late effects of CRP suppression.

© 2026 Foley ÉM et al. *JAMA Psychiatry*.

At follow-ups, participants completed questionnaires, cognitive tests (day 14 only), and gave blood samples. Adverse events and side effects were recorded. Blinding success was assessed by asking participants and staff to guess allocation at exit.

2. Clinical scales

2.1. Clinical Interview Schedule – Revised (CIS-R)

Depression diagnosis was confirmed using the CIS-R, a fully structured, standardised assessment tool widely used to administer self-completed questionnaires in research settings to measure common mental health disorders[4]. The CIS-R assesses 14 symptom areas, namely fatigue, appetite, sleep problems, concentration difficulties, irritability, depression, depressive ideas, anxiety, worry, panic, phobia, compulsive behaviours, obsessive thoughts, and somatic symptoms. Scores range from 0-4 per section, excluding depressive ideas which has a maximum possible score of five. Using this information, diagnostic categories were then generated in line with ICD-10 criteria, including diagnosis of depression using the recognised cut-off score of ≥12[4].

2.2. Patient Health Questionnaire (PHQ)-9

The PHQ-9 is a self-administered questionnaire based on the nine Diagnostic and Statistical Manual of Mental Disorders – IV criteria for depressive disorders[5]. This scale was used as an indicator of the presence of depressive symptoms.

2.3. Primary outcome – Depression somatic symptoms

Total depression somatic symptoms score was the primary outcome for this trial. Existing studies, including our own work strongly, suggest that out of all symptoms of depression, the so-called somatic (or neurovegetative) symptoms such as fatigue are more strongly associated with inflammation than psychological symptoms such as hopelessness or excessive/inappropriate guilt[6–10]. Our choice of primary outcome was informed by this body of evidence as this symptom dimension is more mechanistically relevant for immunotherapy. As there are no validated tools for assessing somatic symptoms of depression alone, we took a pragmatic approach by using somatic symptom related items from the well-established BDI-II scale. Depression somatic symptoms score was calculated by summing: items 4 (lack

© 2026 Foley ÉM et al. *JAMA Psychiatry*.

of pleasure), 15 (loss of energy), 16 (changes in sleeping pattern), 18 (changes in appetite), 19 (concentration difficulties), 20 (tiredness or fatigue), and 21 (loss of interest in sex). Scores could range from 0-21.

2.4. Secondary outcomes – Depression severity and domain-specific scores

The BDI-II was used to assess severity of depression. The 21 items in the questionnaire were coded on a 4-point scale ranging from 0 to 3 and a total score was calculated for each participant ranging between 0-63.

A novel depression psychological symptoms score was calculated by summing 11 relevant BDI-II items: items 1 (sadness), 2 (pessimism), 3 (past failure), 5 (guilty feelings), 6 (punishment feelings), 7 (self-dislike), 8 (self-criticalness), 9 (suicidal thoughts or wishes), 10 (crying), 14 (worthlessness), and 17 (irritability). Scores could range from 0-33.

2.5. Fatigue

The Multidimensional Fatigue Inventory (MFI) is a reliable 20-item tool widely used for assessing fatigue[11,12]. Scores could range from 20-100. Total scores for five dimensions of fatigue were also calculated, namely general fatigue, physical fatigue, mental fatigue, reduced activity, and reduced motivation. Scores ranged from 5-20 per dimension.

2.6. Anhedonia

The Snaith-Hamilton Pleasure Scale (SHAPS)[13] is a 14-item, user friendly, reliable and valid tool used to measure anhedonia in psychiatric conditions, including depression[14,15]. Items on this 14-item scale were coded as 0 = agree and 1 = disagree with a total score of 0-14.

2.7. Anxiety

The State-Trait Anxiety Inventory (STAI), a valid and sensitive measure[16], was used to measure state anxiety (STAI-S)[17]. The scale has 20 items coded on a 4-point scale and scores could range from 20-80.

© 2026 Foley ÉM et al. *JAMA Psychiatry*.

2.8. *Quality of life*

The EuroQol five-dimension three-level (EQ-5D-3L) test was used to assess quality of life[18]. This scale assesses five dimensions of quality of life, namely, mobility, self-care, usual activities, pain/discomfort, and anxiety/depression. Index score ranging from approximately 0 to a maximum of 1 were calculated (according to user manuals), with 1 representing perfect health.

3. Cognitive scales

3.1. *National Adult Reading Test (NART)*

The NART was used to assess estimated premorbid intelligence[19]. Participants were asked to pronounce 50 words with irregular spellings in British English (e.g., aisle). Scores were then converted, according to user manuals, to predict intelligence quotient (IQ) scores on the Weschler Adult Intelligence Scale[20].

3.2. *Cold cognition*

3.2.1 Psychomotor speed

Psychomotor speed was assessed using a symbol digit coding test based on the THINC-integrated tool[21]. In this task, participants were asked to copy symbols corresponding to numbers as quickly and as accurately as possible within 90 seconds. The outcome measure was number of correctly coded symbols.

3.2.2 Reaction time

Reaction time was assessed using the Cambridge Neuropsychological Test Automated Battery (CANTAB)[22] Reaction Time test, five-choice mode. Upon the appearance of a target, participants were asked to react as quickly as possible by releasing a button at the bottom of the screen and selecting the circle in which a dot had appeared. The outcome measure was median reaction time.

3.2.3 Visual associative learning and memory

Visual associative learning and memory was assessed using the CANTAB Paired Associates Learning test. Objects were momentarily displayed in boxes shown on screen in a random order. Participants were then shown each object individually and asked to identify which box

© 2026 Foley ÉM et al. *JAMA Psychiatry*.

they were originally located in. The outcome measure was the total number of errors adjusted (i.e., total number of errors plus an adjustment for number of stages not reached).

3.2.4 Executive function

Executive function (i.e., spatial planning and working memory subdomains) was assessed using the CANTAB One Touch Stockings of Cambridge test. Participants were presented with three coloured balls stacked in two different patterns. They were asked to decipher the minimum number of moves required to match one pattern with another. The outcome measure was number of problems solved in first choice.

3.2.5 Sustained attention

The CANTAB Rapid Visual Information Processing test was used to assess sustained attention. Participants are asked to detect target sequences of digits (e.g., 2–4–6, 4–7–9) that appeared among other rapidly presented (100 digits/minute) pseudo-random digits (ranging from 2 to 9). The outcome measure for this task was median response latency.

3.3. Hot cognition

3.3.1 Affective bias

The Emotional Categorisation and Recall Task (ECAT)[23] was used to assess affective bias and comprised of two stages: categorisation and recall. In the categorisation stage, participants are presented with 60 words of changing valence, 30 positive (e.g., cheerful) and 30 negative (e.g., hostile), and asked to categorise these words as likeable or dislikeable personality traits. The outcome measures for this stage of the task were reaction time for positive and negative words. After a delay of 15 minutes, participants were then asked to recall as many of the 60 words as possible. The outcome measures for this recall stage were total positive and total negative words recalled. Higher scores reflect a positive bias.

3.3.2 Perceptual bias

The CANTAB Emotion Bias Task (EBT) happy-to-sad variant was used to assess perceptual bias in facial emotion perception. Participants are briefly shown faces (150 ms) morphed between two emotions (happy and sad) of varied intensities and are asked to identify the emotion displayed using a two-alternative forced choice. The outcome measure was bias

point (i.e., the proportion of trials selected as happy compared to sad) adjusted to a scale of 0–15. Higher scores reflect a positive bias.

4. Collection of biological samples and immune protein assay protocols

Blood samples were collected from non-fasting participants at eligibility, baseline, and 7-, 14-, and 28-days post-infusion by trained research staff. Samples were transported to the Core Biochemical Assay Laboratory at Addenbrooke's Hospital, Cambridge on the day of collection and analysed by laboratory staff according to pre-specified manufacturer's protocols. Laboratory staff were blind to study arm allocation and performance in clinical and cognitive outcome assessments.

Serum concentrations of hs-CRP, interferon (IFN)-γ, IL-4, IL-6, IL-8, IL-10, IL-12p70, IL-13, TNF-α, sIL-6R, and soluble glycoprotein 130 (sgp130) were obtained. Immunoassay panels were designed prior to participant recruitment and were run by laboratory staff as previously described[7]. Specifically, upon collection blood samples were promptly centrifuged at 1600 g for 15 minutes at room temperature. Serum was transferred into prelabelled 500 µl aliquots and frozen at -70°C. Serum hs-CRP levels were obtained using an automated colorimetric immunoassay on the Siemens Dimension EXL analyser. Serum IFN-γ, IL-4, IL-6, IL-8, IL-10, IL-12p70, IL-13, and TNF-α were measured using the MesoScale Discovery (MSD) 10-plex Human Proinflammatory Panel. SIL-6R was measured using a single MSD R-Plex assay and a Quantikine enzyme-linked immunosorbent assay (ELISA; R&D Systems) was performed to measure sgp130. Upper and lower limits of detection for each inflammatory marker are presented (**eTable 3**). Immune proteins with values below the detection limit were assigned the correspondent lower limit value (e.g., IL-6 <0.5pg/mL = 0.5pg/mL)[24]. No inflammatory marker had values above their limit of detection.

5. Statistical analysis

All statistical analyses were performed using R version 4.4.0[25] and Stata release 18[26]. Study outcomes were analysed based on all participants who were randomised and received infusion. Analyses were conducted in line with the statistical analysis plan proposed in the Insight Study protocol paper[27].

When the study was designed, no RCTs of anti-inflammatory treatment in depression were available to inform target treatment effect size for our primary outcome (somatic

© 2026 Foley ÉM et al. *JAMA Psychiatry*.

symptom score). We estimated that N=50 sample size would provide ~80% power (alpha=0.05) to detect a 2.5-point reduction in CIS-R depression score, using assumptions from a previous RCT[28]. Recruitment was affected by the COVID-19 pandemic, and the study was closed with a final N=30 sample. As a small proof-of-concept trial, the study was not powered for definitive efficacy conclusions. The primary focus was on the overall pattern of results, rather than individual statistical significance tests, with interpretation based on effect size magnitude, confidence intervals, and pattern of change over time relative to clinically meaningful thresholds, when available.

Prespecified analyses included the multivariable regression analyses on primary, secondary, exploratory, and cognitive outcomes, assessment of drug safety and adherence, and blinding success. Post-hoc analyses included repeated measures regression, immune protein tertile analyses, depression remission and response, BDI-II item-level analyses, and sample size calculations for future trials.

Treatment arms (tocilizumab vs placebo) were compared at baseline using descriptive statistics.

5.1. Immune protein concentrations and evidence of target engagement

To assess the success of target engagement and examine possible off-target effects, the mean (SD) of immune protein markers were calculated at baseline and 7-, 14-, and 28-days post-infusion, for each trial arm (i.e., tocilizumab vs placebo). Analyses for immune markers are based on 27 participants. Of the 30 trial participants, three were removed from immune protein concentration analyses: one participant exited the study after baseline assessment due to COVID-19 study pause and thus, did not receive an infusion or attend follow-up assessments; two participants showed evidence of infection at follow-up as determined by elevated levels of inflammatory markers at a single timepoint post-infusion. Their immune markers were outliers compared to the rest of the participants. To assess the relationship between IL-6 and hs-CRP at baseline, we conducted Pearson's correlation test, using log-transformed values to account for positive skew.

© 2026 Foley ÉM et al. *JAMA Psychiatry*.

5.2. Regression analyses

Multivariable regression was performed to test the effect of tocilizumab on primary, secondary, and tertiary clinical and cognitive outcomes at each of the three follow-up timepoints (7-, 14-, and 28-days post-infusion). Two regression models were run in each instance: Model 1 = adjusted for baseline score for the respective outcome to account for initial severity; Model 2 = further adjustments for additional covariates (i.e., for clinical outcomes = BMI, due to its established association with inflammation and depression; for cognitive analyses = NART score, as an estimate of premorbid IQ influencing cognitive performance), minimisation variables (i.e., sex and BDI-II baseline depression severity total score; to reflect the randomisation process), imbalances between groups at baseline identified by qualitative comparisons of descriptive statistics (i.e., duration of current antidepressant treatment in weeks).

Model 1 (R code example):

```
lm (outcome_follow_up ~ trial_arm + outcome_baseline, data = dat)
```

Model 2 (R code example):

```
lm (outcome_follow_up ~ trial_arm + outcome_baseline + BMI + sex +
    depression_severity_baseline + current_antidep_weeks, data = dat)
```

Assumption checks were conducted to test the validity of the regression models. Multicollinearity was assessed using Variance Inflation Factors (VIF), where values >5 indicated significant multicollinearity and potential distortion of the results due to highly correlated predictors. The normality of residuals was checked both visually through histograms and Q-Q plots, and statistically using the Shapiro-Wilk test, with $p>0.05$ suggesting that the residuals followed a normal distribution. Homoscedasticity was tested using the Breusch-Pagan test, where $p<0.05$ indicated the presence of heteroscedasticity. Outliers were identified using Cook's Distance, with values exceeding 4/n indicating influential data points, and manual exploration of flagged data points. For all regression models, effect estimates were robust to influential points and so no outliers were ultimately excluded. Finally, autocorrelation of residuals was tested using the Durbin-Watson test, with $p<0.05$ suggesting that the residuals were not independent. Autocorrelation of residuals was tested using the Durbin-Watson test, with $p<0.05$ suggesting that the residuals were not independent.

© 2026 Foley ÉM et al. *JAMA Psychiatry*.

R Code example:

```
vif(lm_model_outcome) #multicollinearity
hist(resid(lm_model_outcome) #normality of residuals
qqnorm(resid(lm_model_outcome))
qqline(resid(md_adj_somat.f1))
shapiro.test(resid(lm_model_outcome))
plot(fitted(lm_model_outcome), resid(lm_model_outcome) #homoscedasticity
bptest(lm_model_outcome)
plot(cooks.distance(lm_model_outcome), type="h") #outliers
which((cooks.distance(lm_model_outcome)) > 4 /
    length(cooks.distance(lm_model_outcome)))
dwtest(lm_model_outcome) #autocorrelation
```

Regression models of depression psychological symptoms were not additionally adjusted for BDI-II baseline depression severity score (a minimisation variable) due to identified risk of multicollinearity. The adjusted mean difference and 95% confidence intervals was then calculated between trial arms per outcome for each model. Robust standard errors (SEs) were calculated for regression models that violated the assumptions of normality and/or heteroscedasticity.

Standard approach (R code example):

```
emmeans.1 <- emmeans (reg_model.1, ~ trial_arm)
summ.1 <- summary (contrast (emmeans.1, "revpairwise"))
diff.1 <- summ.1$estimate
se.1 <- summ.1$SE
lci.1 <- diff.1 - 1.96 * se.1
uci.1 <- diff.1 + 1.96 * se.1
```

Robust SEs (R code example):

```
rses_reg_model.1 <-  vcovHC (reg_model.1, type = "HC3")
emmeans.1 <- emmeans (regression_outcome.1, ~ trial_arm, , vcov.=
        rses_reg_model.1)
summ.1 <- summary (contrast (emmeans.1, "revpairwise"))
```

© 2026 Foley ÉM et al. *JAMA Psychiatry*.

```
diff.1 <- summ.1$estimate
se.1 <- summ.1$SE
lci.1 <- diff.1 - 1.96 * se.1
uci.1 <- diff.1 + 1.96 * se.1
```

6. Results

6.1. Outlier exclusion

Data on immunological protein concentrations are presented for 28 participants (tocilizumab n=13, placebo n=15), after excluding two participants (one from each trial arm) who had extreme outlier values for several immune proteins, possibly due to infection during follow-up (**eTable8**). For example, one participant in the placebo group had a serum hs-CRP concentration of 7.64mg/L at baseline and 9.45mg/L at follow-up one. However, at follow-up two their hs-CRP rose to 25.11 mg/L, alongside a marked increase in IFN-γ to 353.72 pg/mL, consistent with possible infection. As these extreme values substantially skewed mean (SD) estimates, they were excluded from these analyses.

6.2. Pharmacodynamics of IL-6R inhibition with tocilizumab

After tocilizumab treatment, despite hs-CRP reduction and inhibition of inflammation, IL-6 and sIL-6R levels increase in plasma[29]. This is because IL-6R-mediated consumption of IL-6 is inhibited by the unavailability of tocilizumab-free IL-6R, and sIL-6R elimination half-life is prolonged by the formation of tocilizumab/sIL-6R immune complex. These increases do not reflect increase in IL-6 activity, rather decreased clearance. As long as free tocilizumab is detectable, sIL-6R remains saturated with tocilizumab and IL-6 signalling is completely inhibited.

6.3. Repeated measures regression

Repeated measures regression analyses were conducted post hoc to identify trajectories of outcome scores across the three follow-ups and included an interaction between treatment arm and follow-up timepoint. The model consisted of fixed effects for trial arm (tocilizumab

© 2026 Foley ÉM et al. *JAMA Psychiatry*.

or placebo), timepoint (1, 2, or 3), and their interaction (trial arm × timepoint), with a random intercept for each participant to account for repeated measurements. From these fully adjusted models, we obtained estimated effects (regression coefficients and pairwise contrasts) per timepoint per trial arm. A negative regression coefficient indicates a more beneficial (greater reduction in symptom severity) treatment effect over time favouring tocilizumab. Pairwise comparisons of these estimated effects were performed to assess whether trajectories differed between trial arms.

Stata code example:

```
mixed score i.trial_arm ## i.timepoint outcome_baseline bmi sex
        depression_severity_baseline med_weeks || id:
testparm timepoint # trial_arm
margins timepoint # trial_arm, pwcompare (cimargins pveffects)
margins trial_arm, dydx (timepoint) pwcompare(cimargins pveffects)
```

6.4. *Minimal clinically important differences (MCIDs)*

6.4.1 Fatigue

The estimated MCID for the MFI total score is 16.60 points[30]. The mean difference (95% CI) in the Insight Study was -7.04 (-16.72, 2.64), indicating that the treatment effect is within the range of clinically meaningful improvement.

For the five MFI subdomains, mean differences (95% CIs; MCIDs) at final follow-up were -2.58 (-4.87, -0.29; MCID = 2.39 [50]) for reduced activity, -1.84 (-4.03, 0.35; MCID = 2.04 [50]) for physical fatigue, -1.57 (-3.87, 0.73; MCID = 2.06 [50]) for general fatigue, -0.64 (-3.32, 2.05; MCID = 1.60 [50]) for reduced motivation, and -0.33 (-2.83, 2.18; MCID = 1.36 [50]) for mental fatigue. This suggests clinically meaningful improvements in activity, physical, and general fatigue cannot be ruled out.

6.4.2 State anxiety

Estimated MCID for the STAIS scale is 8 points [31]. The mean difference (95% CI) in the Insight Study was -2.01 (-10.16, 6.13), suggesting that the treatment effect is within the range of clinically meaningful improvement.

© 2026 Foley ÉM et al. *JAMA Psychiatry*.

6.4.3 Quality of life

The estimated MCID for the EQ-5D-3L index is 0.03-0.05 points [32]. The mean difference (95% CI) in the Insight Study was 0.14 (-0.08, 0.35), indicating that the treatment effect is within the range of clinically meaningful improvement.

6.5. Effect of baseline inflammation level

To assess the influence of baseline inflammatory markers, we additionally calculated estimated effects post hoc for four key outcomes (primary outcome: depression somatic symptoms; secondary outcome: total depression severity; tertiary/exploratory outcomes: depression psychological symptoms; and total fatigue) stratified by baseline hs-CRP and IL-6 tertiles. Tertiles were chosen to represent low, medium, and high hs-CRP and IL-6 levels in the sample. Hs-CRP thresholds: ≥3, ≥5, ≥7 mg/L; IL-6 thresholds: ≥0.5, ≥1.17, ≥1.76 pg/mL.

R Code example:

```
crp_ter_fct <- function(threshold) {
        filtered_data <- dat_long %>% filter(hsCRP_baseline >= threshold)
        model <- lme(outcome_follow_up ~ timepoint * trial_arm + BMI + sex +
                depression_severity_baseline + current_antidep_weeks,
                random = ~timepoint | id,
                data = filtered_data)
        return(summary(model))}
summary_crp3_bdi <- crp_ter_fct(3)
summary_crp5_bdi <- crp_ter_fct(5)
summary_crp7_bdi <- crp_ter_fct(7)
```

Results for depression somatic symptoms, depression psychological symptoms, and total depression severity, are provided and discussed in the main manuscript (see Results and **Figure 2**). Total fatigue score did not show any evidence of an altered magnitude of improvement in symptoms based on baseline hs-CRP or IL-6 levels (**eFigure 6**).

© 2026 Foley ÉM et al. *JAMA Psychiatry*.

6.6. *Effect of treatment on depression remission and response*

Using the BDI-II classification system[33], participants were categorised by depression levels (based on BDI-II total score) at baseline and last follow-up, split by trial arm: 1-10 (Normal), 11-16 (Mild), 17-20 (Borderline), 21-30 (Moderate), 31-40 (Severe), and over 40 (Extreme). For the binary outcome of depression remission (i.e. BDI-II total score $\leq$20) and response (within-person 50% reduction in BDI-II total score from baseline to last follow-up), we calculated risk difference (RD) and the number needed to treat (NNT; the inverse of RD) at last follow-up between the treatment and placebo groups. NNT values were rounded up to the next whole number to provide a conservative estimate. When 95% CIs for the RD included zero, NNT values were expressed using the NNT for harm (NNTH) to ∞ to NNT for benefit (NNTB) notation to indicate that the interval included no effect.

6.7. *Effect of treatment on specific depressive symptoms*

To assess treatment effect on specific symptoms, we calculated median scores for each of the 21 items on the BDI-II scale per timepoint split by trial arm. Results were plotted using *ggplot* to allow for qualitative comparison of symptom trajectories over time. We next calculated change scores from baseline to last follow-up (approximately 28-days post-infusion) for each participant per individual item on the BDI-II scale. Group-level medians were then calculated per item per trial arm and plotted using grouped bar plots. To aid visualisation, scores of zero were recoded as -0.01. Negative values reflect symptom improvement.

6.8. *Effect of treatment on anhedonia*

Tocilizumab seemed to have little impact on anhedonia (**eInformation**; **eTables 6**). Several factors may explain this, including measurement, timing of effect, power, and the possibility of no effect. First, the measure used (SHAPS) primarily captures consummatory pleasure (i.e., enjoyment of activities) rather than anticipatory/motivational aspects of reward which may be more sensitive to inflammatory mechanisms[34]. It is also known that patients often experience difficulty in interpreting some items on anhedonia scales like the SHAPS. Second, the short follow-up and single-dose design may not be sufficient to influence motivational systems, which could require sustained treatment. Third, the small sample size reduces precision and while the data are compatible with anything from no effect to a notable effect,

© 2026 Foley ÉM et al. *JAMA Psychiatry*.

this study is not sufficiently powered to distinguish between these possibilities. Forth, while there is much interest in the effect of inflammation on anhedonia, it is possible that anhedonia is not sensitive to IL-6/IL-6R antagonism.

In future, we recommend larger trials with longer treatment duration that incorporate more sensitive and granular measures of reward processing, such as tasks assessing anticipatory motivation and effort-based decision-making, alongside self-report scales.

6.9. Required sample size for future RCTs

Post-hoc, we estimated the minimum sample size per trial arm (assuming equal allocation) required to detect the observed standardised effect sizes with 80% or 90% power (1 minus Type II error probability), using two-sided tests at a 5% significance level (Type I error probability). For the binary outcomes of depression remission and response, we used a two-sample test for independent proportions based on the standardised difference in proportions (Cohen's h). For continuous outcomes of BDI-II total depression severity score and depression somatic symptoms score, we used two-sample t-test with the standardised mean difference (SMD), calculated by dividing the baseline-adjusted mean difference by the pooled residual standard deviation (SD) from linear regression models.

We also estimated the minimum sample size required to detect a minimum clinically important difference (MCID) of 20% in mean BDI-II total depression score. The MCID was defined as a 20% reduction in the mean score in the treatment arm compared with the observed mean score in the placebo arm at the last follow-up. This calculation assumed equal variances between trial arms and was based on the observed SDs of scores at the last follow-up.

For the primary outcome, the BDI-II depression somatic symptoms score, the baseline-adjusted mean difference (MD) between treatment and placebo groups at last follow-up was -0.81 (95% CI: -3.32, 1.70), corresponding to an SMD of -0.25 (95% CI: -1.01, 0.52). The minimum required sample size to detect this effect with 90% power was 338 participants per arm. For the BDI-II total depression score, the baseline-adjusted MD was -2.10 (95% CI: -9.18, 4.98), with an SMD of -0.26 (95% CI: -1.11, 0.60), and required sample size of 312 per arm (**eTable11**).

To assess the ability to detect an MCID for the BDI-II total depression score, we calculated a 20% reduction from the observed mean score (24.06) in the placebo arm at the last follow up. This corresponds to an expected mean score of 19.25 (SD=14.10) in the

© 2026 Foley ÉM et al. *JAMA Psychiatry*.

treatment arm at the last follow up. The resulting MD between groups, defined as the difference between the treatment mean (19.25) and the placebo mean (24.06) at last follow-up, was estimated at -4.81 (95% CI: -14.18, 4.56), corresponding to an SMD of -0.38 (95% CI: -1.11, 0.36). A total of 147 participants per arm would be required to achieve 90% power to detect this difference.

For the binary outcome of depression remission and response, the standardised effect size (Cohen's h) was 0.46 (95% CI: -0.23, 1.15) and 0.60 (95% CI: -0.13, 1.33), respectively, with a minimum required sample size of 99 per arm (remission) and 60 per arm (response) for 90% power (**eTable11**).

Post-hoc power and sample size calculations have limitations because they rely on observed effect sizes that may be imprecise in small or early-phase studies such as proof-of-concept trials. Consequently, these calculations should be interpreted with caution and used only as preliminary guidance, alongside confidence intervals for observed effect sizes and clinical judgment, when planning larger trials[35,36].

© 2026 Foley ÉM et al. *JAMA Psychiatry*.

eTables

eTable 1. Inclusion and exclusion criteria for the Insight Study

Group	Inclusion Criteria	Exclusion Criteria
All patients	Provide informed consent.	Current or lifetime diagnosis of bipolar disorder, psychotic disorder, personality disorder or eating disorder.
	Aged 20-65 years (inclusive).	Current suicidal thoughts or history of suicide attempt, deliberate self-harm, overdose within 6 months prior to eligibility assessment.
	Meet ICD-10 criteria for diagnosis of depression at eligibility assessment.	History of alcohol or substance use disorder (abuse/ dependence) within 6 months prior to eligibility assessment.
	BDI-II depression somatic symptom score ≥7 at eligibility assessment[a].	History of serious allergic reaction after any infusion.
	Taking an antidepressant at an adequate dose (according to the BNF) for ≥4 weeks at eligibility assessment.	Pregnant or breast feeding.
	Understand written and spoken English.	Current use of medication likely to compromise interpretation of immunological data (i.e., antibiotics, non-steroidal anti-inflammatories, oral or injectable corticosteroids).
	Able to consent to blood sampling.	Any major episode of infection requiring hospitalisation or treatment with intravenous antibiotics within 4 weeks of eligibility assessment.

© 2026 Foley ÉM et al. *JAMA Psychiatry*.

	Willing to abstain from strenuous exercise for 72 hours prior to assessment.	Presence or history of recurrent bacterial, viral, fungal, mycobacterial or other opportunistic infections; unstable cardiac, pulmonary, renal, hepatic, endocrine, haematological or active infectious disease; rheumatic autoimmune disease, mixed connective tissue disease, scleroderma, polymyositis; uncontrolled hypertension.
		No history of chickenpox infection or no history of varicella zoster vaccination.
Intervention cohort	Serum hs-CRP ≥ 3mg/L.	Current or past infection with TB, hepatitis B, hepatitis C, HIV, or VZV.
		History of severe allergic or anaphylactic reactions to human, humanised or murine monoclonal antibodies.

[a] Depression somatic symptoms score was calculated by summing seven relevant items on the BDI-II[33], as previously[7,8]: items 4 (lack of pleasure), 15 (loss of energy), 16 (changes in sleeping pattern), 18 (changes in appetite), 19 (concentration difficulties), 20 (tiredness or fatigue), and 21 (loss of interest in sex). Scores could range from 0-21. *Note:* Table reproduced from Khandaker et al. (2018) with permission from the first author. BDI-II, Beck's Depression Inventory-II; BNF, British National Formulary; hs-CRP, high-sensitivity C-reactive protein; HIV, human immunodeficiency virus; ICD-10, International Classification of Diseases 10th Revision; TB, tuberculosis; VZV, Varicella Zoster Virus.

© 2026 Foley ÉM et al. *JAMA Psychiatry*.

eTable 2. The Insight Study outcome measures and time of assessment

Domain	Tool	Time of assessment
Sample characteristics	Screening questionnaire	Screening
	Sociodemographic questionnaire	Baseline
	Antidepressant history and concomitant treatment questionnaire	Baseline
	Drug and alcohol questionnaire	Baseline
Psychiatric	Patient Health Questionnaire 9	Screening
	Clinical Interview Scheduled – Revised	Eligibility
	Beck's Depression Inventory – II	Eligibility, baseline, follow-ups
	Snaith-Hamilton Pleasure Scale Questionnaire	Baseline, follow-ups
	Multidimensional Fatigue Inventory	Baseline, follow-ups
	Visual analogue Scales for Subjective Feelings	Baseline, follow-ups
	Perceived Stress Scale	Baseline, follow-ups
Cognitive	National Adult Reading Scale for Estimated Premorbid IQ	Baseline
	Emotional Categorisation and Recall Task	Baseline, follow-up 2
	Symbol digit coding test	Baseline, follow-up 2
	Cambridge Neuropsychological Test Automated Battery (CANTAB) Reaction Time	Baseline, follow-up 2
	CANTAB Rapid Visual Information Processing	Baseline, follow-up 2
	CANTAB Paired Associates Learning	Baseline, follow-up 2
Biological	Inflammatory markers, cardiometabolic markers, indoleamine 2,3-dioxygenase (IDO) activation, immune cell phenotyping	Baseline, follow-ups

© 2026 Foley ÉM et al. *JAMA Psychiatry*.

eTable 3. Lower and upper detection limits for inflammatory proteins measured in the Insight Study

Immune Protein	Lower Limit of Detection	Upper Limit of Detection	Total N Below Lower Limit of Detection
Hs-CRP (mg/L)	0.2	~18[a]	4
IFN-γ (pg/mL)	1.9	3100	0
IL-2 (pg/mL)	0.5	2840	77
IL-4 (pg/mL)	0.1	512	73
IL-6 (pg/mL)	0.5	1456	23
SIL-6R (ng/mL)	0.03	100	0
IL-8 (pg/mL)	0.3	1142	0
IL-10 (pg/mL)	0.2	748	28
IL-12p70 (pg/mL)	0.3	986	52
IL-13 (pg/mL)	1.3	992	53
Sgp130 (ng/mL)	8	1600	0
TNF-α (pg/mL)	0.3	784	0

[a] Samples repeated on dilution; hs-CRP, high-sensitivity C-reactive protein; IFN, interferon; IL, interleukin; sgp130, soluble glycoprotein 130; sIL-6R, soluble IL-6 receptor; TNF, tumour necrosis factor.

© 2026 Foley ÉM et al. *JAMA Psychiatry*.

eTable 4. Sociodemographic, clinical, and cognitive characteristics of the Insight Study participants at baseline

Characteristic	Total (N=30)	Tocilizumab Arm (n=14)	Placebo Arm (n=16)
Age			
Mean (SD)	41.10 (12.32)	40.71 (11.89)	41.43 (13.07)
Median [IQR]	44.38 [30.76, 47.50]	44.38 [31.68, 46.81]	44.43 [29.71, 48.28]
Sex, N (%)			
Female	24 (80.0%)	12 (85.7%)	12 (75.0%)
Male	6 (20.0%)	2 (14.3%)	4 (25.0%)
BMI			
Mean (SD)	37.17 (8.51)	39.60 (9.50)	35.04 (7.18)
Median [IQR]	36.13 [31.26, 41.09]	39.03 [33.94, 48.01]	34.79 [30.12, 40.89]
Hs-CRP (mg/L)			
Mean (SD)	9.54 (5.00)	10.15 (5.43)	9.01 (4.70)
Median [IQR]	8.55 [6.15, 11.29]	9.38 [6.58, 11.50]	7.56 [6.30, 10.88]
IL-6 (pg/L) [a]			
Mean (SD)	1.57 (0.80)	1.88 (0.86)	1.31 (0.68)
Median [IQR]	1.20 [0.92, 2.15]	1.96 [1.11, 2.21]	1.16 [0.89, 1.48]

© 2026 Foley ÉM et al. *JAMA Psychiatry*.

Characteristic	Total (N=30)	Tocilizumab Arm (n=14)	Placebo Arm (n=16)
Ethnicity, N (%)			
White	27 (90.0%)	13 (92.9%)	14 (87.5%)
Other	3 (10.0%)	1 (7.1%)	2 (12.5%)
Educational Attainment, N (%)			
GCSE/O/A levels	8 (26.7%)	3 (21.4%)	5 (31.3%)
Vocational/College	8 (26.7%)	3 (21.4%)	5 (31.3%)
University/Professional	14 (46.7%)	8 (57.1%)	6 (37.5%)
Employment Status, N (%)			
Unemployed	7 (23.3%)	3 (21.4%)	4 (25.0%)
Employed	23 (76.7%)	11 (78.6%)	12 (75.0%)
Current Medication, N (%)			
SSRI	17 (56.7%)	9 (64.3%)	8 (50.0%)
Other	13 (43.3%)	5 (35.7%)	8 (50.0%)
Current Medication Duration (yrs)			
Mean (SD)	2.05 (3.48)	2.67 (4.87)	1.50 (1.47)
Median [IQR]	1.00 [0.59, 1.99]	1.05 [0.59, 2.91]	1.00 [0.45, 1.99]

© 2026 Foley ÉM et al. *JAMA Psychiatry*.

Characteristic	Total (N=30)	Tocilizumab Arm (n=14)	Placebo Arm (n=16)
Past Depressive Episodes, N (%)			
First episode	5 (16.7%)	4 (28.6%)	1 (6.3%)
2-3	9 (30.0%)	3 (21.4%)	6 (37.5%)
4-5	4 (13.3%)	2 (14.3%)	2 (12.5%)
≥6	12 (40.0%)	5 (35.7%)	7 (43.8%)
National Adult Reading Test [b]			
Mean (SD)	117.40 (6.15)	119.30 (5.95)	115.60 (5.98)
Median [IQR]	118.60 [113.70, 121.90]	120.70 [117.20, 123.20]	117.00 [111.60, 119.00]
BDI-II depression somatic symptoms score			
Mean (SD)	12.23 (3.03)	12.14 (3.06)	12.31 (3.09)
Median [IQR]	12.00 [10.00, 15.00]	11 [9.25, 15.00]	13.00 [10.00, 14.25]
BDI-II depression total score			
Mean (SD)	35.13 (9.80)	35.79 (10.56)	34.56 (9.39)
Median [IQR]	33.50 [26.50, 42.50]	35.50 [27.00, 44.50]	33.00 [27.50, 40.25]
BDI-II depression psychological symptoms score			
Mean (SD)	17.87 (6.16)	18.36 (6.06)	17.44 (6.42)
Median [IQR]	17.50 [12.25, 23.00]	19.00 [13.25, 22.75]	16.00 [12.75, 23.25]

© 2026 Foley ÉM et al. *JAMA Psychiatry*.

Characteristic	Total (N=30)	Tocilizumab Arm (n=14)	Placebo Arm (n=16)
MFI total fatigue score			
Mean (SD)	84.80 (8.60)	84.57 (9.48)	85.00 (8.06)
Median [IQR]	86.00 [81.50, 90.75]	87.00 [84.25, 90.00]	85.00 [80.50, 91.50]
STAI state anxiety score			
Mean (SD)	59.50 (11.14)	59.93 (10.45)	59.12 (12.05)
Median [IQR]	60.00 [51.50, 67.00]	60.00 [51.50, 68.00]	61.50 [52.00, 67.00]
SHAPS anhedonia score			
Mean (SD)	5.63 (3.51)	5.14 (3.82)	6.06 (3.28)
Median [IQR]	6.00 [3.00, 7.75]	5.50 [2.25, 7.00]	6.50 [4.50, 8.00]
EQ-5D-3L quality of life score			
Mean (SD)	0.45 (0.29)	0.52 (0.23)	0.39 (0.34)
Median [IQR]	0.52 [0.18, 0.69]	0.53 [0.34, 0.70]	0.45 [0.06, 0.69]
Psychomotor speed: Digit symbol coding score [c]			
Mean (SD)	62.48 (17.07)	59.36 (18.37)	65.40 (15.84)
Median [IQR]	63.00 [57.00, 74.00]	63.50 [44.25, 72.00]	63.00 [59.00, 76.50]
Executive function: CANTAB One Touch Stockings of Cambridge score [d]			
Mean (SD)	10.63 (2.844)	11.15 (2.70)	10.14 (2.98)
Median [IQR]	11.00 [9.00, 12.00]	11.00 [9.00, 14.00]	11.00 [9.50, 11.00]

© 2026 Foley ÉM et al. *JAMA Psychiatry*.

Characteristic	Total (N=30)	Tocilizumab Arm (n=14)	Placebo Arm (n=16)
Sustained attention: CANTAB Rapid Visual Information Processing score [c]			
Mean (SD)	453.00 (88.51)	465.00 (76.91)	441.77 (99.48)
Median [IQR]	431.00 [392.50, 500.50]	463.00 [404.50, 516.10]	428.00 [387.80, 460.00]
Learning and memory: CANTAB Paired Associates Learning score [c]			
Mean (SD)	-13.62 (14.02)	-15.36 (16.37)	-12.00 (11.77)
Median [IQR]	-8.00 [-15.00, -4.00]	-9.00 [-15.00, -5.50]	-6.00 [-17.00, -4.00]
Reaction time: CANTAB Reaction Time (five-choice) score [c]			
Mean (SD)	370.80 (48.42)	384.43 (54.56)	358.17 (39.61)
Median [IQR]	362.00 [341.00, 388.00]	380.80 [353.00, 394.40]	345.00 [328.00, 381.00]
Positive affective bias: ECAT positive word recall total score [c]			
Mean (SD)	4.10 (2.09)	4.57 (2.41)	3.67 (1.72)
Median [IQR]	4.00 [3.00, 5.00]	4.50 [3.00, 7.00]	3.00 [2.50, 4.50]
Negative affective bias: ECAT negative word recall total score [c]			
Mean (SD)	3.03 (1.92)	3.07 (2.27)	3.00 (1.60)
Median [IQR]	2.00 [2.00, 4.00]	2.50 [2.00, 4.00]	2.00 [2.00, 4.50]
Perceptual bias: CANTAB Emotion Bias Test score [d]			
Mean (SD)	7.16 (1.34)	7.10 (1.63)	7.21 (1.06)

© 2026 Foley ÉM et al. *JAMA Psychiatry*.

Characteristic	Total (N=30)	Tocilizumab Arm (n=14)	Placebo Arm (n=16)
Median [IQR]	7.00 [6.50, 8.00]	7.00 [6.33, 8.00]	7.00 [6.67, 7.92]

[a] Total N=29, tocilizumab N=13, placebo N=16. [b] Total N=29, tocilizumab N=14, placebo N=15. [c] Total N=29, tocilizumab N=14, placebo N=15. [d] Total N=27, tocilizumab N=13, placebo N=14; BMI, body mass index; IQR, interquartile range; SD, standard deviation; SSRI, selective serotonin reuptake inhibitor.

© 2026 Foley ÉM et al. *JAMA Psychiatry*.

eTable 5. Clinical and cognitive outcome scores per timepoint split by sex

Outcome and Measure	Time	Outcome score, Mean (SD)[a]			
		Female		Male	
		Tocilizumab (n=12)	Placebo (n=12)	Tocilizumab (n=2)	Placebo (n=4)
BDI-II depression somatic symptoms score (primary outcome)	Baseline	12.00 (3.19)	12.25 (3.39)	13.00 (2.83)	12.50 (2.38)
	Follow-up 1	9.36 (4.72)	10.08 (3.53)	12.00 (2.83)	7.75 (6.13)
	Follow-up 2	8.45 (4.50)	9.58 (4.25)	10.50 (0.71)	8.50 (5.80)
	Follow-up 3	7.00 (4.52)	9.00 (3.05)	11.00 (5.66)	8.50 (5.92)
MFI total fatigue score	Baseline	83.67 (9.85)	83.58 (7.80)	90.00 (5.66)	89.25 (8.34)
	Follow-up 1	74.09 (13.19)	80.00 (10.21)	91.50 (3.54)	78.00 (11.69)
	Follow-up 2	70.73 (14.31)	78.00 (11.36)	85.50 (2.12)	78.25 (16.15)
	Follow-up 3	66.55 (14.68)	77.92 (9.13)	89.50 (9.19)	76.25 (16.78)
MFI subdomain: Reduced activity score	Baseline	15.83 (3.74)	14.67 (3.55)	18.00 (1.41)	18.00 (3.37)
	Follow-up 1	13.18 (3.43)	14.42 (3.73)	20.00 (0.00)	16.75 (3.77)
	Follow-up 2	12.91 (3.83)	13.25 (3.82)	16.50 (3.54)	15.75 (4.35)
	Follow-up 3	11.00 (3.00)	14.08 (2.47)	16.50 (0.71)	14.75 (5.50)
	Baseline	16.42 (3.29)	17.33 (2.31)	18.00 (0.00)	18.00 (1.41)

© 2026 Foley ÉM et al. *JAMA Psychiatry*.

Outcome and Measure	Time	Outcome score, Mean (SD)[a]			
		Female		Male	
		Tocilizumab (n=12)	Placebo (n=12)	Tocilizumab (n=2)	Placebo (n=4)
MFI subdomain: Physical fatigue score	Follow-up 1	14.91 (2.30)	16.67 (2.67)	18.00 (1.41)	18.00 (1.63)
	Follow-up 2	15.27 (2.90)	17.08 (2.47)	17.50 (2.12)	18.25 (1.26)
	Follow-up 3	13.64 (2.94)	16.50 (2.71)	20.00 (0.00)	18.25 (1.71)
MFI subdomain: General fatigue score	Baseline	17.92 (1.78)	18.75 (1.14)	18.50 (2.12)	18.75 (1.89)
	Follow-up 1	15.82 (3.25)	17.75 (2.05)	19.00 (0.00)	16.75 (2.99)
	Follow-up 2	15.36 (3.44)	17.42 (1.88)	18.50 (0.71)	17.00 (2.94)
	Follow-up 3	14.09 (3.65)	17.50 (1.73)	18.50 (2.12)	15.25 (3.77)
MFI subdomain: Reduced motivation score	Baseline	16.50 (1.73)	16.08 (2.50)	16.50 (0.71)	15.25 (2.06)
	Follow-up 1	15.09 (3.51)	15.42 (3.00)	17.50 (2.12)	12.25 (2.99)
	Follow-up 2	13.82 (3.68)	14.50 (3.90)	15.50 (2.12)	12.50 (4.36)
	Follow-up 3	13.73 (4.47)	14.67 (3.39)	17.50 (3.54)	13.75 (2.99)
MFI subdomain: Mental fatigue score	Baseline	17.00 (2.17)	16.75 (3.14)	19.00 (1.41)	19.25 (0.96)
	Follow-up 1	15.09 (3.70)	15.75 (1.76)	17.00 (0.00)	14.25 (2.75)
	Follow-up 2	13.36 (3.96)	15.75 (2.67)	17.50 (3.54)	14.75 (4.43)

© 2026 Foley ÉM et al. *JAMA Psychiatry*.

**Outcome and Measure**	**Time**	**Outcome score, Mean (SD)[a]**			
		**Female**		**Male**	
		**Tocilizumab (n=12)**	**Placebo (n=12)**	**Tocilizumab (n=2)**	**Placebo (n=4)**
	Follow-up 3	14.09 (4.28)	15.17 (2.89)	17.00 (4.24)	14.25 (3.77)
BDI-II depression total score (secondary outcome)	Baseline	34.75 (10.99)	35.50 (10.21)	42.00 (5.66)	31.75 (6.65)
	Follow-up 1	26.18 (12.86)	28.08 (12.87)	39.50 (7.78)	20.25 (15.09)
	Follow-up 2	22.00 (12.37)	25.58 (15.36)	38.00 (0.00)	19.25 (16.21)
	Follow-up 3	19.27 (11.90)	25.08 (11.20)	40.00 (15.56)	21.00 (14.31)
BDI-II depression psychological symptoms score	Baseline	17.75 (6.36)	18.33 (6.79)	22.00 (1.41)	14.75 (4.92)
	Follow-up 1	13.09 (6.71)	14.08 (7.90)	21.50 (2.12)	9.50 (7.85)
	Follow-up 2	10.27 (6.34)	12.42 (9.36)	23.00 (0.00)	8.50 (8.96)
	Follow-up 3	9.64 (6.19)	12.83 (7.40)	23.50 (6.36)	10.25 (8.42)
STAI state anxiety score	Baseline	58.00 (9.97)	59.33 (12.47)	71.50 (3.54)	58.50 (12.45)
	Follow-up 1	54.27 (14.60)	55.08 (12.24)	70.00 (7.07)	46.75 (15.33)
	Follow-up 2	48.82 (14.13)	52.58 (14.14)	66.50 (4.95)	45.50 (12.71)
	Follow-up 3	46.82 (14.70)	51.33 (12.94)	70.00 (5.66)	51.25 (14.03)
SHAPS anhedonia score	Baseline	4.67 (3.92)	6.33 (3.52)	8.00 (1.41)	5.25 (2.63)

© 2026 Foley ÉM et al. *JAMA Psychiatry*.

Outcome and Measure	Time	Outcome score, Mean (SD)[a]			
		Female		Male	
		Tocilizumab (n=12)	Placebo (n=12)	Tocilizumab (n=2)	Placebo (n=4)
	Follow-up 1	3.82 (3.06)	4.00 (3.25)	4.00 (4.24)	3.50 (4.04)
	Follow-up 2	2.73 (3.13)	4.00 (3.69)	9.00 (7.07)	3.00 (2.16)
	Follow-up 3	2.82 (4.05)	3.42 (2.39)	4.50 (6.36)	4.25 (4.19)
EQ-5D-3L quality of life score	Baseline	0.53 (0.23)	0.44 (0.32)	0.44 (0.26)	0.23 (0.39)
	Follow-up 1	0.65 (0.16)	0.43 (0.32)	0.27 (0.03)	0.43 (0.46)
	Follow-up 2	0.63 (0.27)	0.54 (0.29)	0.55 (0.42)	0.48 (0.44)
	Follow-up 3	0.73 (0.12)	0.49 (0.37)	0.27 (0.03)	0.39 (0.35)
Psychomotor speed: Symbol digit coding test score	Baseline	60.08 (18.32)	68.91 (15.27)	55.00 (25.46)	55.75 (14.97)
	Follow-up 2	69.36 (16.32)	70.73 (3.04)	64.50 (20.51)	61.50 (19.50)
Executive function: CANTAB One Touch Stockings of Cambridge score	Baseline	11.27 (2.94)	10.64 (1.43)	10.50 (0.71)	8.33 (6.43)
	Follow-up 2	10.00 (2.90)	11.18 (1.99)	13.00 (1.41)	11.00 (3.56)
Sustained attention: CANTAB Rapid Visual Information Processing score	Baseline	476.33 (77.50)	443.82 (107.44)	397.00 (6.36)	436.12 (87.41)
	Follow-up 2	464.23 (93.26)	453.77 (114.67)	382.75 (29.34)	452.38 (115.81)

© 2026 Foley ÉM et al. *JAMA Psychiatry*.

Outcome and Measure	Time	Outcome score, Mean (SD)[a]			
		Female		Male	
		Tocilizumab (n=12)	Placebo (n=12)	Tocilizumab (n=2)	Placebo (n=4)
Learning and memory: CANTAB Paired Associates Learning score	Baseline	-14.17 (15.82)	-8.64 (7.57)	-22.50 (24.75)	-21.25 (17.31)
	Follow-up 2	-13.36 (16.62)	-9.64 (8.46)	-4.50 (0.71)	-11.50 (10.47)
Reaction time: CANTAB Reaction Time (five-choice) score	Baseline	395.00 (50.46)	362.68 (42.68)	321.00 (36.06)	345.75 (31.12)
	Follow-up 2	375.73 (39.24)	353.45 (32.64)	302.50 (20.51)	333.12 (53.75)
Positive affective bias: ECAT positive word recall total score	Baseline	4.75 (2.09)	4.00 (1.84)	3.50 (4.95)	2.75 (0.96)
	Follow-up 2	6.36 (3.50)	4.73 (3.32)	4.00 (0.00)	5.25 (4.35)
Negative affective bias: ECAT negative word recall total score	Baseline	2.83 (1.40)	3.27 (1.68)	4.50 (6.36)	2.25 (1.26)
	Follow-up 2	2.73 (0.79)	4.00 (1.95)	6.50 (4.95)	2.25 (1.50)
Perceptual bias: CANTAB Emotion Bias (happy-to-sad variant) *score*	Baseline	6.91 (1.63)	7.00 (1.10)	8.16 (1.65)	8.00 (0.33)
	Follow-up 2	7.18 (1.87)	7.09 (1.40)	8.17 (2.12)	7.33 (1.36)

Note: For all clinical outcomes, females: baseline N = 24 (tocilizumab = 12, placebo = 12), follow-ups N = 23 (tocilizumab =11, placebo = 12). For cognitive outcomes, females: baseline N = 23 (tocilizumab = 12, placebo = 11), follow-up N = 22 (tocilizumab =11, placebo = 11). For executive function and perceptual bias, males: tocilizumab arm N = 2, placebo arm N = 3. BDI-II, Beck Depression Inventory-II; CANTAB, Cambridge Neuropsychological Test Automated Battery; CI, confidence intervals; ECAT, The Emotional Categorisation and Recall Task; EQ-5D-3L, EuroQol five-dimension three-level; MFI, Multidimensional Fatigue Inventory; SD, standard deviation; SHAPS, Snaith-Hamilton Pleasure Scale; STAI, State-Trait Anxiety Inventory.

© 2026 Foley ÉM et al. *JAMA Psychiatry*.

eTable 6. Immune protein concentrations of Insight Study participants at baseline and post-infusion split by sex

Immune Protein	Trial Arm[a]	Female, mean (SD) (n=22)				Male, mean (SD) (n=6)			
		Baseline	Follow-up 1	Follow-up 2	Follow-up 3	Baseline	Follow-up 1	Follow-up 2	Follow-up 3
Hs-CRP (mg/L)	TCZ	11.20 (5.64)	0.65 (0.42)	0.45 (0.47)	0.67 (0.91)	5.19 (1.09)	0.28 (0.25)	0.20 (0.14)	0.15 (0.07)
	PBO	9.08 (5.53)	11.96 (9.55)	8.42 (5.38)	8.70 (8.52)	9.13 (2.83)	6.16 (1.71)	9.46 (4.06)	8.92 (3.04)
IL-6 (pg/mL)	TCZ	2.10 (0.83)	19.23 (8.36)	19.60 (7.85)	18.20 (8.66)	1.27 (0.70)	13.23 (3.23)	12.86 (3.07)	15.40 (9.95)
	PBO	1.39 (0.81)	1.44 (0.75)	1.32 (0.61)	1.57 (1.07)	1.11 (0.14)	1.18 (0.18)	1.50 (0.58)	1.12 (0.23)
SIL-6R (ng/mL)	TCZ	47.87 (12.94)	64.37 (14.84)	81.41 (9.68)	90.47 (15.01)	48.64 (15.81)	70.11 (18.10)	74.41 (27.76)	100.00 (0.00)
	PBO	46.56 (18.46)	45.84 (17.84)	45.88 (18.31)	45.87 (17.08)	57.92 (10.36)	56.14 (11.43)	52.75 (8.98)	55.59 (13.80)
Sgp130 (ng/mL)	TCZ	225.62 (30.08)	241.60 (40.53)	247.12 (34.83)	239.00 (45.30)	237.10 (66.89)	274.95 (41.65)	258.55 (23.69)	273.65 (15.77)
	PBO	234.12 (46.96)	225.20 (45.19)	222.23 (58.28)	222.15 (45.06)	236.55 (39.19)	240.65 (48.35)	249.68 (63.80)	211.23 (61.36)
TNF-α (pg/mL)	TCZ	2.18 (1.10)	1.90 (0.80)	1.98 (1.00)	1.76 (0.74)	1.85 (0.08)	1.84 (0.28)	1.72 (0.42)	2.06 (0.17)
	PBO	1.73 (0.33)	1.61 (0.38)	1.63 (0.38)	1.66 (0.37)	2.07 (0.32)	1.96 (0.40)	1.84 (0.39)	2.30 (0.66)
IFN-γ (pg/mL)	TCZ	11.94 (8.08)	10.73 (4.05)	12.77 (7.71)	8.94 (3.38)	4.53 (0.35)	6.99 (1.59)	6.66 (0.54)	7.10 (0.44)
	PBO	5.94 (2.08)	8.01 (6.00)	6.78 (3.99)	12.50 (20.09)	6.46 (1.11)	5.71 (0.63)	5.82 (1.46)	7.63 (2.84)

© 2026 Foley ÉM et al. *JAMA Psychiatry*.

Immune Protein	Trial Arm[a]	Female, mean (SD) (n=22)				Male, mean (SD) (n=6)			
		Baseline	Follow-up 1	Follow-up 2	Follow-up 3	Baseline	Follow-up 1	Follow-up 2	Follow-up 3
IL-4 (pg/mL)	TCZ	0.11 (0.01)	0.11 (0.02)	0.10 (0.00)	0.11 (0.02)	0.10 (0.00)	0.10 (0.00)	0.10 (0.00)	0.11 (0.02)
	PBO	0.10 (0.00)	0.10 (0.00)	0.10 (0.00)	0.10 (0.00)	0.10 (0.01)	0.11 (0.01)	0.11 (0.02)	0.11 (0.01)
IL-8 (pg/mL)	TCZ	12.32 (5.32)	14.18 (5.07)	12.21 (3.97)	10.89 (3.35)	10.95 (6.29)	13.10 (1.05)	11.81 (1.85)	12.56 (3.42)
	PBO	15.68 (7.47)	12.41 (7.05)	13.77 (7.71)	12.60 (4.65)	13.98 (3.67)	12.28 (5.74)	10.62 (1.98)	16.40 (10.54)
IL-10 (pg/mL)	TCZ	0.39 (0.20)	0.33 (0.22)	0.31 (0.17)	0.34 (0.20)	0.20 (0.00)	0.21 (0.01)	0.25 (0.07)	0.20 (0.00)
	PBO	0.26 (0.16)	0.24 (0.04)	0.22 (0.04)	0.25 (0.07)	0.37 (0.11)	0.29 (0.11)	0.33 (0.15)	0.24 (0.02)
IL-12p70 (pg/mL)	TCZ	0.58 (0.78)	0.59 (0.80)	0.53 (0.56)	0.49 (0.51)	0.60 (0.42)	0.65 (0.47)	0.54 (0.34)	0.71 (0.59)
	PBO	0.36 (0.12)	0.32 (0.03)	0.34 (0.08)	0.32 (0.05)	0.49 (0.29)	0.46 (0.26)	0.53 (0.34)	0.47 (0.30)
IL-13 (pg/mL)	TCZ	1.85 (1.20)	1.58 (0.79)	1.62 (0.86)	1.59 (0.92)	1.40 (0.14)	1.30 (0.00)	1.30 (0.00)	1.43 (0.19)
	PBO	1.62 (0.54)	1.43 (0.15)	1.52 (0.32)	1.68 (0.51)	1.31 (0.03)	1.30 (0.01)	1.34 (0.06)	1.54 (0.41)

[a] N for females = 11 in TCZ arm and 11 in PBO arm; N for males = 2 in TCZ arm and 4 in PBO arm. PBO, placebo. Two female participants (one from each trial arm) who had extreme outlier values for several proteins, possibly because of an infection acquired during the follow-up, were excluded from immunological analyses. Hs-CRP, high-sensitivity C-reactive protein; IFN, interferon; IL, interleukin; SD, standard deviation; sgp130, soluble glycoprotein 130; sIL-6R, soluble IL-6 receptor; TCZ, tocilizumab; TNF, tumour necrosis factor.

© 2026 Foley ÉM et al. *JAMA Psychiatry*.

eTable 7. Immune protein concentrations of Insight Study participants at baseline and post-infusion

Immune Protein	Trial Arm	N	Mean (SD)			
			Baseline	Follow-Up 1	Follow-Up 2	Follow-Up 3
Hs-CRP (mg/L)	Tocilizumab	13	10.28 (5.63)	0.59 (0.41)	0.40 (0.43)	0.58 (0.84)
	Placebo	15	9.10 (4.85)	10.30 (8.44)	8.72 (4.91)	8.75 (7.49)
IL-6 (pg/mL)	Tocilizumab	13	1.96 (0.84)	18.23 (7.97)	18.38 (7.60)	17.73 (8.46)
	Placebo	15	1.32 (0.70)	1.37 (0.64)	1.37 (0.59)	1.47 (0.95)
sIL-6R (ng/mL)	Tocilizumab	13	48.00 (12.64)	65.33 (14.67)	80.13 (12.65)	92.06 (14.07)
	Placebo	15	49.59 (17.13)	48.78 (16.55)	47.84 (16.16)	48.11 (16.39)
sgp130 (ng/mL)	Tocilizumab	13	227.53 (34.16)	247.16 (40.87)	249.20 (32.37)	244.78 (43.40)
	Placebo	15	234.81 (43.38)	229.61 (44.78)	230.07 (58.79)	219.63 (46.62)
TNF-a (pg/mL)	Tocilizumab	13	2.12 (1.01)	1.89 (0.73)	1.94 (0.91)	1.81 (0.68)
	Placebo	15	1.82 (0.35)	1.71 (0.40)	1.69 (0.38)	1.81 (0.50)
IFN-y (pg/mL)	Tocilizumab	13	10.70 (7.85)	10.11 (3.97)	11.66 (7.33)	8.64 (3.15)
	Placebo	15	6.08 (1.84)	7.35 (5.12)	6.51 (3.42)	11.38 (17.56)
IL-4 (pg/mL)	Tocilizumab	13	0.10 (0.01)	0.11 (0.02)	0.10 (0.00)	0.11 (0.02)
	Placebo	15	0.10 (0.01)	0.10 (0.01)	0.10 (0.01)	0.10 (0.01)
IL-8 (pg/mL)	Tocilizumab	13	12.09 (5.20)	14.00 (4.62)	12.14 (3.61)	11.17 (3.27)
	Placebo	15	15.22 (6.59)	12.37 (6.48)	12.87 (6.65)	13.48 (6.12)
IL-10 (pg/mL)	Tocilizumab	13	0.36 (0.19)	0.31 (0.20)	0.30 (0.16)	0.32 (0.19)
	Placebo	15	0.29 (0.15)	0.26 (0.07)	0.25(0.10)	0.25 (0.06)
IL-12p70	Tocilizumab	13	0.58 (0.71)	0.60 (0.74)	0.53 (0.52)	0.53 (0.50)

© 2026 Foley ÉM et al. *JAMA Psychiatry*.

(pg/mL)	Placebo	15	0.40 (0.18)	0.36 (0.14)	0.39 (0.20)	0.36 (0.14)
IL-13	Tocilizumab	13	1.78 (1.10)	1.53 (0.72)	1.56 (0.78)	1.56 (0.84)
(pg/mL)	Placebo	15	1.54 (0.48)	1.40 (0.14)	1.47 (0.28)	1.65 (0.47)

Note: Data on immunological protein concentrations are presented for 28 participants (tocilizumab n=13, placebo n=15), after excluding two participants (one from each group) who had extreme outlier values for several proteins, possibly due to infection during follow-up. Hs-CRP, high-sensitivity C-reactive protein; IFN, interferon; IL, interleukin; sgp, soluble glycoprotein; sIL-6R, soluble IL-6 receptor; SD standard deviation; TNF, tumour necrosis factor.

© 2026 Foley ÉM et al. *JAMA Psychiatry*.

eTable 8. Effect of tocilizumab treatment on clinical outcomes in the Insight Study

Outcome and Measure	Time	Outcome score, Mean (SD)[a]		Mean Difference (95% CI) between tocilizumab and placebo arms		Longitudinal repeated measures effect size
		Tocilizumab (N=14)[b]	Placebo (N=16)[b]	Model 1 (Adjusted for baseline score)[c]	Model 2 (Adjusted for additional covariates)[c]	Estimated Effect, Change Over Time[d] (95% CI)
BDI-II depression somatic symptoms score (primary outcome)	Baseline	12.14 (3.06)	12.31 (3.09)	-	-	-
	FU1	9.77 (4.49)	9.50 (4.21)	0.81 (-1.70, 3.32) [e]	0.75 (-1.39, 2.89)	-
	FU2	8.77 (4.19)	9.31 (4.50)	0.01 (-2.35, 2.37)	-0.12 (-2.51, 2.28)	-0.81 (-2.63, 1.01)
	FU3	7.62 (4.68)	8.88 (3.72)	-0.81 (-3.32, 1.70)	-0.47 (-2.98, 2.04)	-1.53 (-3.35, 0.29)
BDI-II depression total score (secondary outcome)	Baseline	35.79 (10.56)	34.56 (9.39)	-	-	-
	FU1	28.23 (12.96)	26.12 (13.39)	1.54 (-4.11, 7.19) [e]	2.18 (-3.14, 7.51)	-
	FU2	24.46 (12.79)	24.00 (15.28)	-0.11 (-6.84, 6.63)	0.55 (-6.43, 7.54)	-1.64 (-6.66, 3.37)
	FU3	22.46 (14.10)	24.06 (11.67)	-2.10 (-9.18, 4.98) [e]	-0.87 (-7.35, 5.60)	-3.71 (-8.72, 1.31)

© 2026 Foley ÉM et al. *JAMA Psychiatry*.

Outcome and Measure	Time	Outcome score, Mean (SD)[a]		Mean Difference (95% CI) between tocilizumab and placebo arms		Longitudinal repeated measures effect size
		Tocilizumab (N=14)[b]	Placebo (N=16)[b]	Model 1 (Adjusted for baseline score)[c]	Model 2 (Adjusted for additional covariates)[c]	Estimated Effect, Change Over Time[d] (95% CI)
MFI total fatigue score	Baseline	84.57 (9.48)	85.00 (8.06)	-	-	-
	FU1	76.77 (13.74)	79.5 (10.22)	-2.31 (-10.70, 6.07)	-2.10 (-9.77, 5.56)	-
	FU2	73.00 (14.21)	78.06 (12.12)	-4.56 (-13.66, 4.54)	-3.88 (-12.22, 4.46)	-2.33 (-7.27, 2.61)
	FU3	70.08 (16.15)	77.50 (10.86)	-7.04 (-16.72, 2.64)	-7.26 (-14.95, 0.44)	-4.69 (-9.63, 0.25)
MFI subdomain: Reduced activity score	Baseline	16.14 (3.55)	15.50 (3.71)	-	-	-
	FU1	14.23 (4.04)	15.00 (3.76)	-1.02 (-3.59, 1.55)	-0.90 (-3.22, 1.42)	-
	FU2	13.46 (3.89)	13.88 (3.96)	-0.54 (-3.39, 2.31)	-0.11 (-2.95, 2.72)	0.36 (-1.66, 2.37)
	FU3	11.85 (3.44)	14.25 (3.26)	-2.58 (-4.87, -0.29)	-2.69 (-4.67, -0.72)	-1.63 (-3.65, 0.38)
	Baseline	16.64	17.50	-	-	-

© 2026 Foley ÉM et al. *JAMA Psychiatry*.

Outcome and Measure	Time	Outcome score, Mean (SD)[a]		Mean Difference (95% CI) between tocilizumab and placebo arms		Longitudinal repeated measures effect size
		Tocilizumab (N=14)[b]	Placebo (N=16)[b]	Model 1 (Adjusted for baseline score)[c]	Model 2 (Adjusted for additional covariates)[c]	Estimated Effect, Change Over Time[d] (95% CI)
MFI subdomain: Physical fatigue score		(3.08)	(2.10)			
	FU1	15.38 (2.43)	17.00 (2.48)	-1.03 (-2.63, 0.57) [e]	-0.98 (-2.99, 1.03) [e]	-
	FU2	15.62 (2.84)	17.38 (2.25)	-1.12 (-2.63, 0.39) [e]	-1.07 (-2.79, 0.64) [e]	-0.14 (-1.48, 1.19)
	FU3	14.62 (3.59)	16.94 (2.57)	-1.84 (-4.03, 0.35)	-1.70 (-3.57, 0.17)	-0.71 (-2.04, 0.63)
MFI subdomain: General fatigue score	Baseline	18.00 (1.75)	18.75 (1.29)	-	-	-
	FU1	16.31 (3.20)	17.50 (2.25)	-0.78 (-3.22, 1.66) [e]	-0.72 (-2.78, 1.34)	-
	FU2	15.85 (3.36)	17.31 (2.09)	-0.81 (-2.99, 1.37) [e]	-0.88 (-2.73, 0.97)	-0.27 (-1.42, 0.87)
	FU3	14.77 (3.77)	16.94 (2.46)	-1.57 (-3.87, 0.73)	-1.69 (-3.75, 0.38)	-0.98 (-2.12, 0.17)
	Baseline	16.50 (1.61)	15.88 (2.36)	-	-	-

© 2026 Foley ÉM et al. *JAMA Psychiatry*.

Outcome and Measure	Time	Outcome score, Mean (SD)[a]		Mean Difference (95% CI) between tocilizumab and placebo arms		Longitudinal repeated measures effect size
		Tocilizumab (N=14)[b]	Placebo (N=16)[b]	Model 1 (Adjusted for baseline score)[c]	Model 2 (Adjusted for additional covariates)[c]	Estimated Effect, Change Over Time[d] (95% CI)
MFI subdomain: Reduced motivation score	FU1	15.46 (3.38)	14.62 (3.22)	0.34 (-2.16, 2.83) [e]	0.59 (-1.25, 2.44)	-
	FU2	14.08 (3.48)	14.00 (3.97)	-0.35 (-3.05, 2.35)	-0.18 (-2.65, 2.29)	-0.76 (-2.51, 0.99)
	FU3	14.31 (4.44)	14.44 (3.22)	-0.64 (-3.32, 2.05)	-0.59 (-2.95, 1.76)	-0.97 (-2.71, 0.79)
MFI subdomain: Mental fatigue score	Baseline	17.29 (2.16)	17.38 (2.94)	-	-	-
	FU1	15.38 (3.45)	15.38 (2.06)	0.06 (-1.91, 2.02)	-0.07 (-1.88, 1.74)	-
	FU2	14.00 (4.06)	15.50 (3.06)	-1.42 (-4.00, 1.16) [e]	-1.17 (-3.27, 0.93)	-1.51 (-3.24, 0.22)
	FU3	14.54 (4.24)	14.94 (3.02)	-0.33 (-2.83, 2.18)	-0.62 (-2.91, 1.67)	-0.41 (-2.14, 1.32)
BDI-II depression psychological symptoms score	Baseline	18.36 (6.06)	17.44 (6.42)	-	-	-
	FU1	14.38	12.94	0.81	1.26	-

© 2026 Foley ÉM et al. *JAMA Psychiatry*.

Outcome and Measure	Time	Outcome score, Mean (SD)[a]		Mean Difference (95% CI) between tocilizumab and placebo arms		Longitudinal repeated measures effect size
		Tocilizumab (N=14)[b]	Placebo (N=16)[b]	Model 1 (Adjusted for baseline score)[c]	Model 2 (Adjusted for additional covariates)[c]	Estimated Effect, Change Over Time[d] (95% CI)
		(6.92)	(7.89)	(-2.21, 3.83)	(-1.79, 4.31) [e]	
	FU2	12.23 (7.51)	11.44 (9.13)	0.13 (-4.14, 4.41) [e]	0.83 (-3.26, 4.91) [e]	-0.65 (-3.69, 2.38)
	FU3	11.77 (7.90)	12.19 (7.46)	-1.03 (-4.93, 2.88) [e]	-0.47 (-4.13, 3.19) [e]	-1.87 (-4.90, 1.17)
STAI state anxiety score	Baseline	59.93 (10.45)	59.12 (12.05)	-	-	-
	FU1	56.69 (14.72)	53.00 (13.07)	2.63 (-5.13, 10.39)	3.13 (-4.17, 10.42)	-
	FU2	51.54 (14.58)	50.81 (13.75)	-0.43 (-7.96, 7.10)	-0.71 (-7.72, 6.30)	-2.97 (-8.22, 2.29)
	FU3	50.38 (16.08)	51.31 (12.74)	-2.01 (-10.16, 6.13)	-1.32 (-8.53, 5.89)	-4.62 (-9.87, 0.63)
SHAPS anhedonia score	Baseline	5.14 (3.82)	6.06 (3.28)	-	-	-
	FU1	3.85 (3.05)	3.88 (3.32)	0.31 (-1.68, 2.30)	0.25 (-1.92, 2.42)	-

© 2026 Foley ÉM et al. *JAMA Psychiatry*.

Outcome and Measure	Time	Outcome score, Mean (SD)[a]		Mean Difference (95% CI) between tocilizumab and placebo arms		Longitudinal repeated measures effect size
		Tocilizumab (N=14)[b]	Placebo (N=16)[b]	Model 1 (Adjusted for baseline score)[c]	Model 2 (Adjusted for additional covariates)[c]	Estimated Effect, Change Over Time[d] (95% CI)
	FU2	3.69 (4.23)	3.75 (3.34)	0.31 (-2.09, 2.72)	0.26 (-2.24, 2.76)	-0.03 (-2.25, 2.20)
	FU3	3.08 (4.17)	3.62 (2.80)	-0.19 (-2.79, 2.42) [e]	-0.17 (-2.59, 2.24)	-0.52 (-2.74, 1.70)
EQ-5D-3L quality of life score	Baseline	0.52 (0.23)	0.39 (0.34)	-	-	-
	FU1	0.59 (0.20)	0.43 (0.34)	0.08 (-0.10, 0.26)	0.06 (-0.14, 0.25)	-
	FU2	0.62 (0.27)	0.53 (0.31)	0.03 (-0.18, 0.23)	0.04 (-0.22, 0.30)	-0.07 (-0.26, 0.12)
	FU3	0.66 (0.20)	0.47 (0.36)	0.14 (-0.08, 0.35) [e]	0.14 (-0.07, 0.36) [e]	0.03 (-0.16, 0.22)

[a] Means (SDs) were calculated for each outcome per treatment arm (tocilizumab vs placebo) across each of the four timepoints (baseline, follow-up one, two, and three). Higher scores represented greater symptom severity, except for EQ-5D-3L where high scores indicate greater quality of life. [b] One participant (tocilizumab arm) exited the study after baseline assessment due to COVID-19 study closure and thus, did not receive an infusion or attend follow-up assessments. [c] Multivariable linear regression models were then run to test effect of tocilizumab on each outcome, adjusted as follows: Model 1 = adjusted for baseline outcome score, Model 2 = further adjusted for body mass index, sex, BDI-II baseline total depression severity score, and current antidepressant medication duration. [d] Repeated measures regression was conducted to identify trajectories

© 2026 Foley ÉM et al. *JAMA Psychiatry*.

of symptom scores across the three follow-ups and included an interaction between treatment arm and follow-up timepoint. From these models, we obtained estimated effects (regression coefficients and pairwise contrasts) per timepoint per trial arm, treatment effect varied across time. A negative regression coefficient indicates a more beneficial (greater reduction in symptom severity) treatment effect over time. [e] Robust standard error applied due to violation of linear regression assumptions. BDI-II, Beck Depression Inventory-II; CI, confidence intervals; EQ-5D-3L, EuroQol five-dimension three-level; FU, follow-up; MFI, Multidimensional Fatigue Inventory; SD, standard deviation; SHAPS, Snaith-Hamilton Pleasure Scale; STAI, State-Trait Anxiety Inventory.

© 2026 Foley ÉM et al. *JAMA Psychiatry*.

eTable 9. Insight Study participants depression severity category at baseline according to BDI-II score

BDI-II Total Score	Level of Depression	Tocilizumab, N (%) [a]		Placebo, N (%)	
		Baseline (n=14)	Last Follow-Up (n=13)	Baseline (n=16)	Last Follow-Up (n=16)
1-10	Normal ups and downs	0	3 (23.1)	0	2 (12.5)
11-16	Mild mood disturbance	0	1 (7.7)	0	3 (18.8)
17-20	Borderline clinical depression	0	3 (23.1)	0	0
21-30	Moderate depression	5 (35.7)	4 (30.8)	6 (37.5)	7 (43.8)
31-40	Severe depression	3 (21.4)	0	6 (37.5)	3 (18.9)
Over 40	Extreme depression	6 (42.9)	2 (15.4)	4 (25.0)	1 (6.2)

[a] One participant (tocilizumab arm) exited the study after baseline assessment due to COVID-19 study closure and thus, did not receive an infusion or attend follow-up assessments. BDI-II, Beck Depression Inventory – II

© 2026 Foley ÉM et al. *JAMA Psychiatry*.

eTable 10. Post-hoc analysis of required sample size estimates for depression outcomes for future RCTs

Outcome and Measure	Mean (SD) at final follow-up		Tocilizumab vs Placebo Arm at final follow-up		Sample size required per arm to detect Standardised Effect Size at 80% power in future RCT[c]	Sample size required per arm to detect Standardised Effect Size at 90% power in future RCT[c]
	Tocilizumab (n=13)	Placebo (n=16)	Mean Difference (95% CI)[a]	Standardised Mean Difference (95% CI)[a,b]		
BDI-II total depression score MCID[d]	19.25 (14.10)	24.06 (11.67)	-4.81 (-14.18, 4.56)	-0.38 (-1.11, 0.36)	N=110	N=147
BDI-II depression somatic symptoms score (primary outcome)	7.62 (4.68)	8.88 (3.72)	-0.81 (-3.32, 1.70)	-0.25 (-1.01, 0.52)	N=253	N=338
BDI-II depression total score (secondary outcome)	22.46 (14.10)	24.06 (11.67)	-2.10 (-9.18, 4.98)	-0.26 (-1.11, 0.60)	N=234	N=312
	N (%)	N (%)	Risk Difference (95% CI)[a]	Cohen's H (95% CI)[a]		
Depression remission[e]	7 (53.85)	5 (31.25)	0.23 (-0.13, 0.58)	0.46 (-0.23, 1.15)	N=74	N=99
Depression response[f]	6 (46.15)	3 (18.75)	0.27 (-0.06, 0.61)	0.60 (-0.13, 1.33)	N=45	N=60

[a] 95% confidence intervals were calculated using normal approximation: The point estimate ±1.96 times the standard error; [b] Standardised effect sizes at last follow-up for baseline adjusted depression scores were calculated using residual model SD, and for MCID, depression remission and

© 2026 Foley ÉM et al. *JAMA Psychiatry*.

response using pooled SD; [c] Power calculations used a two-sample t-test for continuous outcomes and a two-sample test for independent proportions for binary outcomes; [d] 20% reduction in BDI-II score in the treatment arm compared to the placebo arm at final follow-up; [e] Depression remission is defined as BDI-II score ≤20; [f] Depression response is defined as 50% reduction in BDI-II total score from baseline to last follow-up; BDI-II, Beck Depression Inventory-II; CI, Confidence Interval; MCID, Minimum Clinically Important Difference; RCT, Randomised Clinical Trial; SD, Standard Deviation.

© 2026 Foley ÉM et al. *JAMA Psychiatry*.

eTable 11. Effect of tocilizumab treatment on cognitive outcomes in the Insight Study

Outcome and Measure	Time	Outcome score, Mean (SD)[a]		Mean Difference (95% CI) between tocilizumab and placebo arms	
		Tocilizumab (N=14)[b]	Placebo (N=14)[b]	Model 1[c] (Adjusted for baseline score)	Model 2[c] (Adjusted for additional for covariates)
Psychomotor speed: Symbol digit coding test score	Baseline	59.36 (18.37)	65.40 (15.84)	-	-
	FU2	68.62 (16.14)	68.27 (14.86)	5.45 (-1.93, 12.82) [e]	3.70 (-4.15, 11.56)
Executive function: CANTAB One Touch Stockings of Cambridge score [d]	Baseline	11.15 (2.70)	10.14 (2.98)	-	-
	FU2	10.46 (2.90)	11.13 (2.36)	-1.24 (-2.76, 0.28)	-1.36 (-2.84, 0.13)
Sustained attention: CANTAB Rapid Visual Information Processing score	Baseline	465.00 (76.91)	441.77 (99.48)	-	-
	FU2	451.69 (90.86)	453.4 (110.75)	-6.19 (-87.04, 74.65) [e]	-1.77 (-90.96, 87.42) [e]
Learning and memory: CANTAB Paired Associates Learning score	Baseline	-15.36 (16.37)	-12.00 (11.77)	-	-
	FU2	-12.00 (15.53)	-10.13 (8.68)	-0.31 (-8.90, 8.28) [e]	2.14 (-6.93, 11.20) [e]

© 2026 Foley ÉM et al. *JAMA Psychiatry*.

Outcome and Measure	Time	Outcome score, Mean (SD)[a]		Mean Difference (95% CI) between tocilizumab and placebo arms	
		Tocilizumab (N=14)[b]	Placebo (N=14)[b]	Model 1[c] (Adjusted for baseline score)	Model 2[c] (Adjusted for additional for covariates)
Reaction time: CANTAB Reaction Time (five-choice) score	Baseline	384.43 (54.56)	358.17 (39.61)	-	-
	FU2	364.46 (45.55)	348.03 (38.29)	0.05 (22.82, 22.93)	1.34 (-25.37, 28.05)
Positive affective bias: ECAT positive word recall total score	Baseline	4.57 (2.41)	3.67 (1.72)	-	-
	FU2	6.00 (3.32)	4.87 (3.46)	0.60 (-1.81, 3.02)[e]	1.25 (-1.00, 3.49)[e]
Negative affective bias: ECAT negative word recall total score	Baseline	3.07 (2.27)	3.00 (1.60)	-	-
	FU2	3.31 (2.14)	3.53 (1.96)	-0.27 (-1.76, 1.23)[e]	-0.38 (-1.74, 0.97)
Perceptual bias: CANTAB Emotion Bias (happy-to-sad variant) *score*[d]	Baseline	7.10 (1.63)	7.21 (1.06)	-	-
	FU2	7.33 (1.85)	7.16 (1.34)	-0.11 (-1.07, 0.84)	-0.10 (-1.18, 0.99)

[a] Means (SDs) were calculated for each outcome per treatment arm (tocilizumab vs placebo) across both timepoints (baseline and follow-up two). Higher scores represented better performance, except for ECAT positive words recall (higher score = more positive words recalled), ECAT

© 2026 Foley ÉM et al. *JAMA Psychiatry*.

negative word recall (higher score = more negative words recalled), and EBT (higher scores represented a positive bias). [b] Cognitive data was collected for 28 participants due to the decision not to collect this data during the COVID-19 pandemic to reduce total face-to-face assessment time. Moreover, one participant (tocilizumab arm) exited the study after baseline assessment due to COVID-19 study closure and thus, did not receive an infusion or attend follow-up assessments. [c] Multivariable linear regression models were then run to test effect of tocilizumab on each outcome, adjusted as follows: Model 1 = adjusted for baseline score, Model 2 = additionally adjusted for NART score, sex, Beck Depression Inventory-II baseline total depression severity score, and current antidepressant treatment duration. [d] N=26 due to data availability. [e] Robust standard error applied due to violation of linear regression assumptions. CANTAB, Cambridge Neuropsychological Test Automated Battery; CI, confidence intervals; ECAT, The Emotional Categorisation and Recall Task; FU, follow-up; NART, National Adult Reading Test; SD, standard deviation.

© 2026 Foley ÉM et al. *JAMA Psychiatry*.

eTable 12. Adverse events

Adverse event	Trial Arm		Total (N=29)
	Tocilizumab (N=13)	Placebo (N=16)	
**Any serious adverse event**	0	0	0
**Any minor adverse event**	8 (62%)	10 (63%)	18 (62%)
**Specific minor adverse events**			
Headache	2 (15%)	6 (38%)	8 (28%)
Cough	0	2 (13%)	2 (7%)
Runny nose	2 (15%)	1 (6%)	3 (10%)
Nausea	1 (8%)	2 (13%)	3 (10%)
Stomach-ache	1 (8%)	0	1 (3%)
Other flu-like symptoms	1 (8%)	2 (13%)	3 (10%)
Fatigue	0	2 (13%)	2 (7%)
Sleep disturbance	0	2 (13%)	2 (7%)
Appetite disturbance	0	1 (6%)	1 (3%)
Mouth ulcers	1 (8%)	0	1 (3%)
Hives	1 (8%)	1 (6%)	2 (7%)
Back pain	0	2 (13%)	2 (7%)
Chest tenderness	1 (8%)	0	1 (3%)
Acne	1 (8%)	0	1 (3%)
Ringworm	0	1 (6%)	1 (3%)

© 2026 Foley ÉM et al. *JAMA Psychiatry*.

eFigures

eFigure 1. Overview of design and procedures for the Insight Study

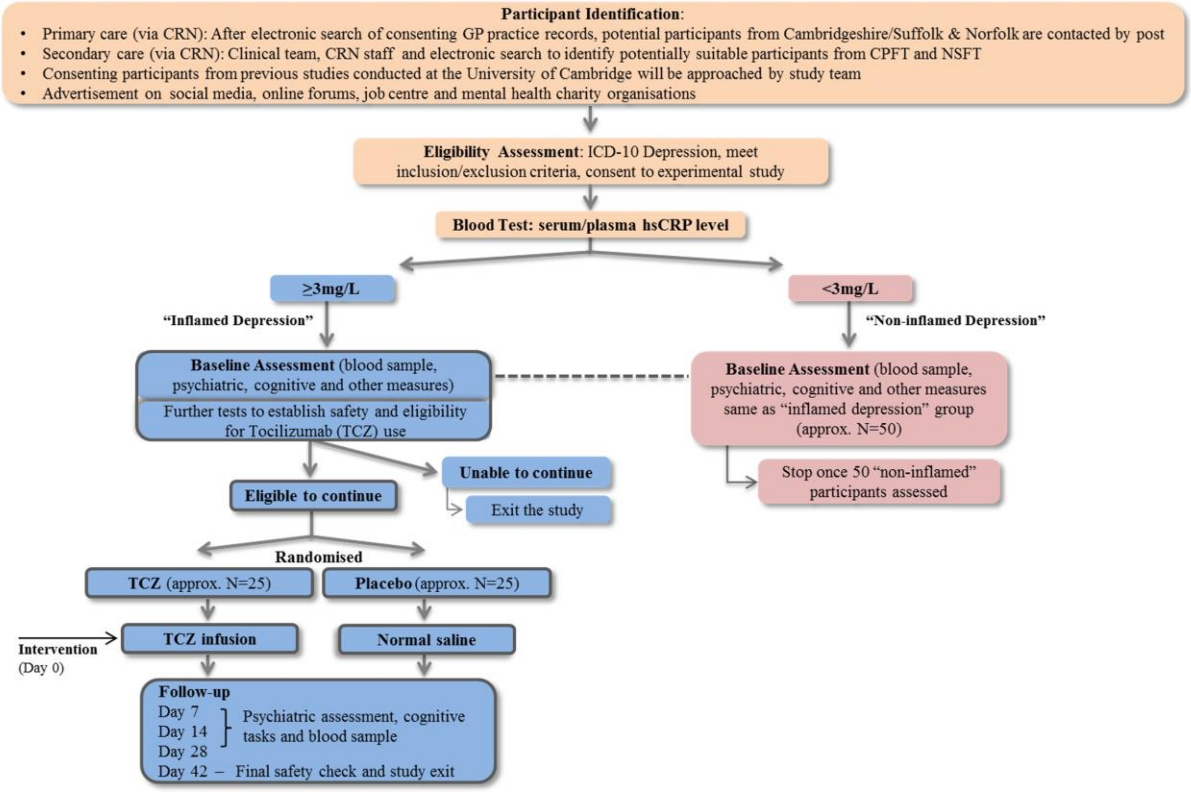

Note: Original figure from Khandaker et al. (2018) reproduced with permission from first author. CRN, clinical research network; CPFT, Cambridgeshire and Peterborough NHS Foundation Trust; GP, general practice; hs-CRP, high-sensitivity C reactive protein; ICD-10, International Classification of Diseases 10th Revision; NSFT, Norfolk and Suffolk NHS Foundation Trust; TCZ, tocilizumab.

© 2026 Foley ÉM et al. *JAMA Psychiatry*.

eFigure 2. The CONSORT 2025[37] flow diagram for the Insight trial

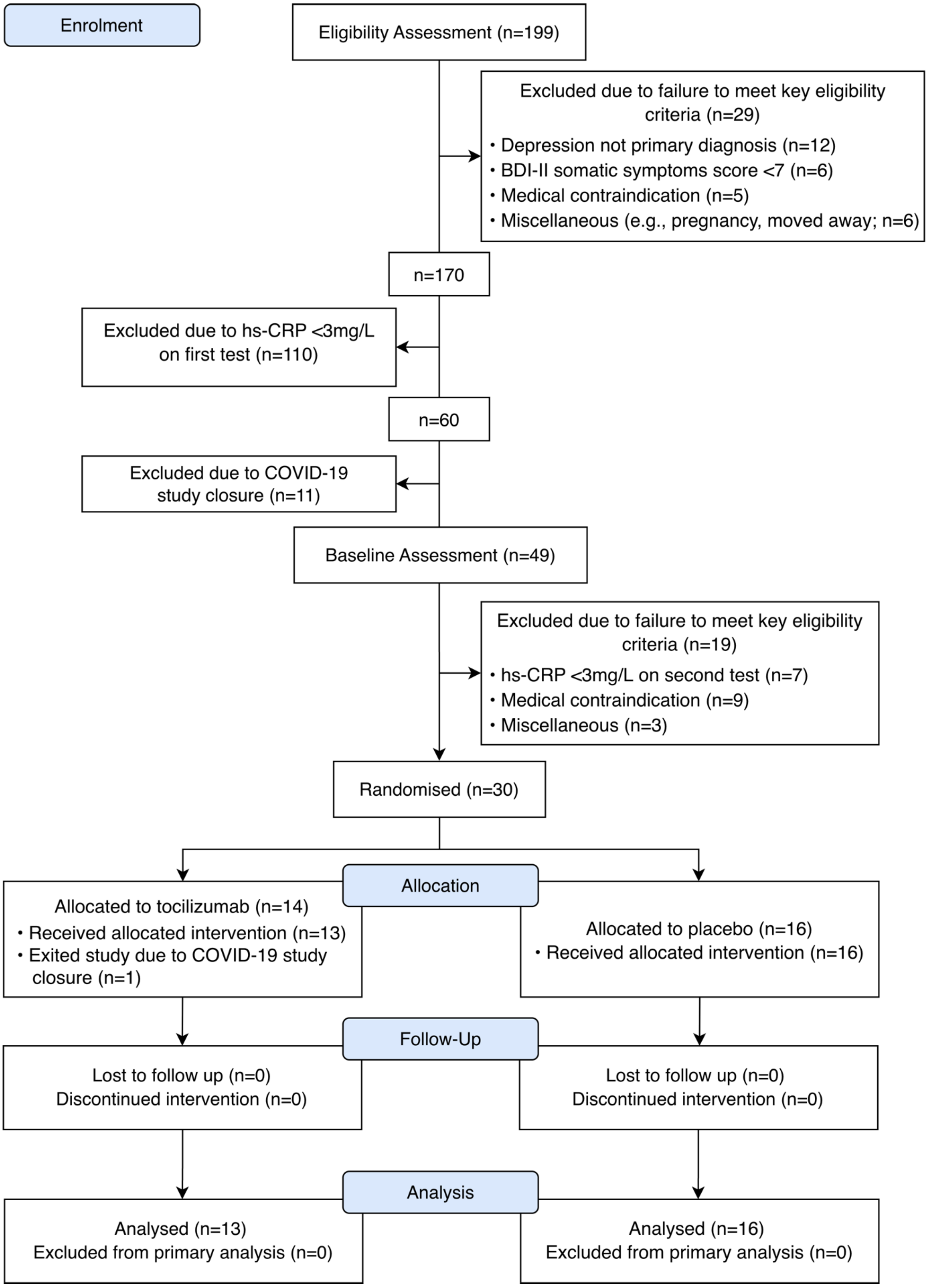

BDI, Beck Depression Inventory; CIS-R, Clinical Interview Schedule Revised; COVID-19, coronavirus disease 2019; hs-CRP, high-sensitivity C-reactive protein.

© 2026 Foley ÉM et al. *JAMA Psychiatry*.

eFigure 3. Serum hs-CRP, IL-6, sIL-6R, and sgp130 levels at baseline and post-infusion in tocilizumab and placebo arms in the Insight Study

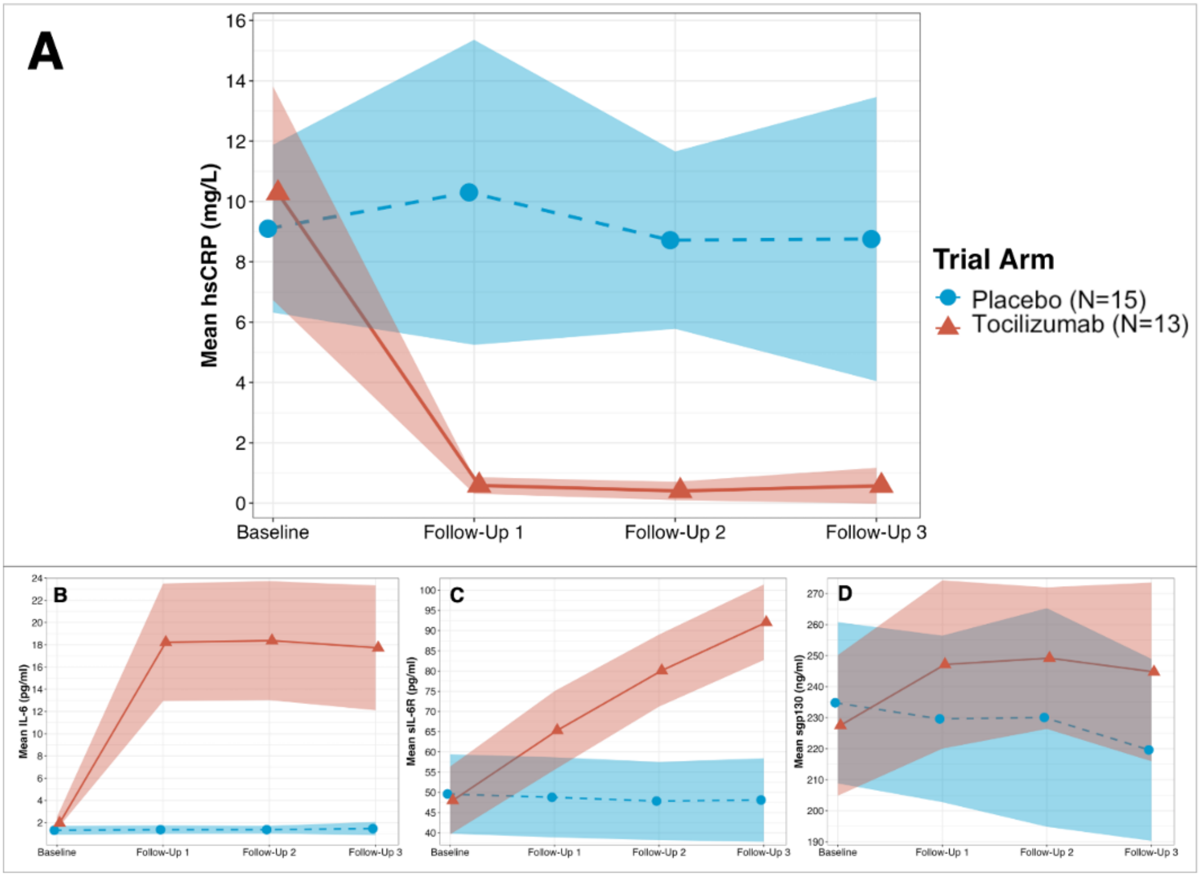

Note: (**A**) hs-CRP, high-sensitivity C-reactive protein; (**B**) IL-6, interleukin 6; (**C**) sIL-6R, soluble IL-6 receptor; (**D**) sgp130, soluble glycoprotein 130. Mean scores are represented by symbols and colours, where blue circles = placebo and red triangles = tocilizumab. Standard deviation is represented by the corresponding cloud colour. Data on immunological proteins concentrations were analysed for 28 participants (tocilizumab n=13, placebo n=15), after removing two participants (one from each group) who had extreme outlier values for several proteins possibly because of an infection acquired during the follow-up.

© 2026 Foley ÉM et al. *JAMA Psychiatry*.

eFigure 4. Effect of tocilizumab treatment on depression remission and response

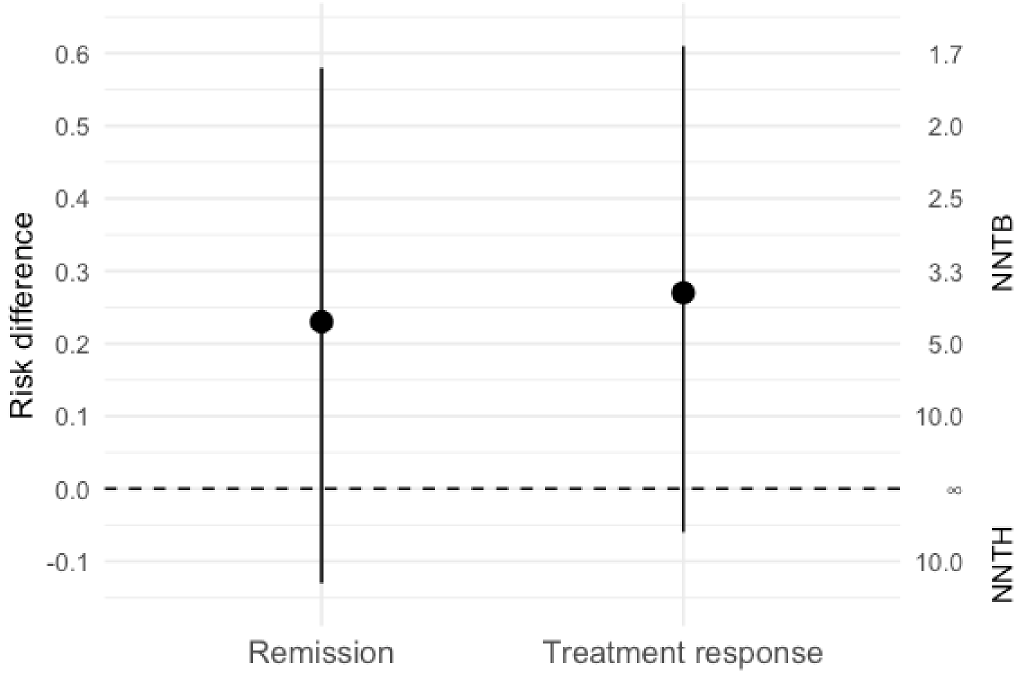

Note: Risk differences (RDs) for depression remission and treatment response at the final follow-up are shown, with 95% confidence intervals (CIs). The secondary axis displays the corresponding number needed to treat (NNT), where positive RDs correspond to NNT for benefit (NNTB) and negative RDs correspond to NNT for harm (NNTH). The point at which RD equals zero corresponds to ∞, indicating no effect. Labels for NNTB and NNTH denote the regions of likely benefit and harm on the NNT scale. Analyses were based on observed proportions in each trial arm at the final follow-up.

© 2026 Foley ÉM et al. *JAMA Psychiatry*.

eFigure 5. Effect of tocilizumab treatment on individual depressive symptoms in the Insight Study

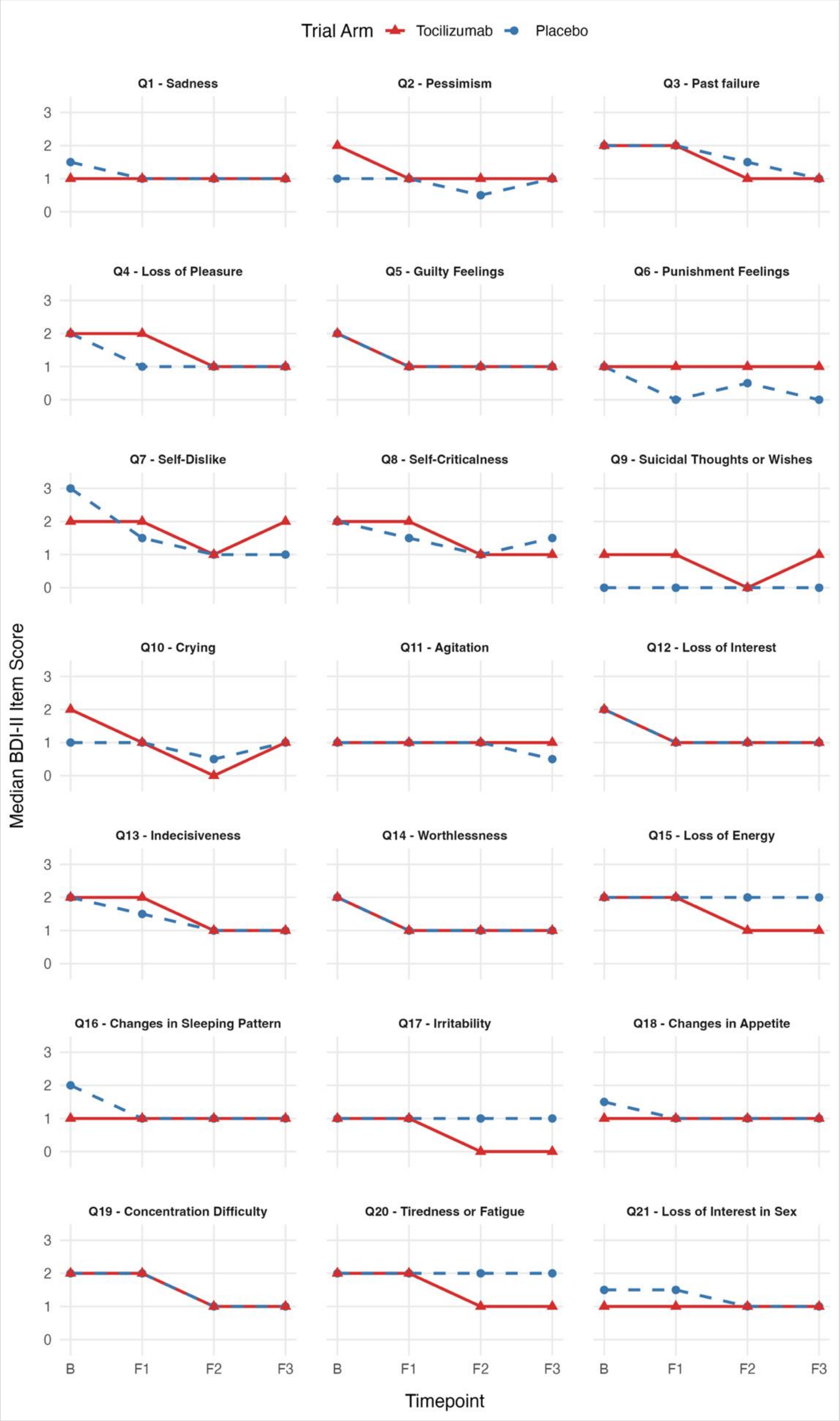

© 2026 Foley ÉM et al. *JAMA Psychiatry*.

Note: Median values for each individual item on the Beck Depression Inventory (BDI)-II scale are presented computed within group (tocilizumab vs placebo) across four timepoints: Baseline (B), Follow-Up 1 (F1), Follow-Up 2 (F2), and Follow-Up 3 (F3). Each line represents the group-level median score per item at each timepoint. Lower scores reflect lower severity of symptoms.

© 2026 Foley ÉM et al. *JAMA Psychiatry*.

eFigure 6. Effect of baseline IL-6 and hs-CRP concentrations on treatment response in total fatigue score in the Insight Study

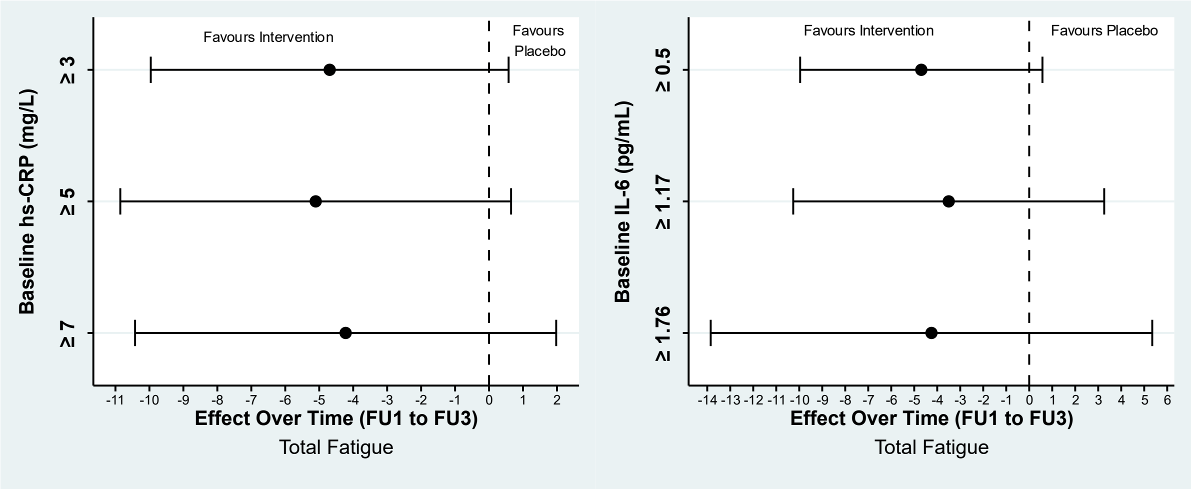

Note: These effects represent the estimated average change in total fatigue score per trial arm over time, stratified by tertiles of baseline hs-CRP and IL-6 concentrations, respectively. Analyses were fully adjusted for baseline score and covariates. Tertiles were chosen to represent low, medium, and high levels of hs-CRP and IL-6 based on the variation of protein levels in our sample. A negative regression coefficient indicates a more beneficial (greater reduction in symptom severity) treatment effect over time favouring tocilizumab.

© 2026 Foley ÉM et al. *JAMA Psychiatry*.

eReferences

1. Leung E, Crass RL, Jorgensen SCJ, et al. Pharmacokinetic/Pharmacodynamic Considerations of Alternate Dosing Strategies of Tocilizumab in COVID-19. *Clin Pharmacokinet*. 2022;61(2):155-165. doi:10.1007/s40262-021-01092-0

2. Choy EHS, Isenberg DA, Garrood T, et al. Therapeutic benefit of blocking interleukin-6 activity with an anti-interleukin-6 receptor monoclonal antibody in rheumatoid arthritis: a randomized, double-blind, placebo-controlled, dose-escalation trial. *Arthritis and rheumatism*. 2002;46(12):3143-3150. doi:10.1002/art.10623

3. Woo P, Wilkinson N, Prieur AM, et al. Open label phase II trial of single, ascending doses of MRA in Caucasian children with severe systemic juvenile idiopathic arthritis: proof of principle of the efficacy of IL-6 receptor blockade in this type of arthritis and demonstration of prolonged clinical improvement. *Arthritis Res Ther*. 2005;7(6):R1281-1288. doi:10.1186/ar1826

4. Lewis G. Assessing psychiatric disorder with a human interviewer or a computer. *Journal of epidemiology and community health*. 1994;48(2):207-210. doi:10.1136/jech.48.2.207

5. Kroenke K, Spitzer RL, Williams JB. The PHQ-9: validity of a brief depression severity measure. *J Gen Intern Med*. 2001;16(9):606-613. doi:10.1046/j.1525-1497.2001.016009606.x

6. Jokela M, Virtanen M, Batty GD, Kivimäki M. Inflammation and Specific Symptoms of Depression. *JAMA Psychiatry*. 2016;73(1):87-88. doi:10.1001/jamapsychiatry.2015.1977

7. Foley ÉM, Slaney C, Donnelly NA, Kaser M, Ziegler L, Khandaker GM. A novel biomarker of interleukin 6 activity and clinical and cognitive outcomes in depression. *Psychoneuroendocrinology*. 2024;164:107008. doi:10.1016/j.psyneuen.2024.107008

8. Foley ÉM, Parkinson JT, Kappelmann N, Khandaker GM. Clinical phenotypes of depressed patients with evidence of inflammation and somatic symptoms. *Comprehensive Psychoneuroendocrinology*. 2021;8:100079. doi:10.1016/j.cpnec.2021.100079

9. Chu AL, Stochl J, Lewis G, Zammit S, Jones PB, Khandaker GM. Longitudinal association between inflammatory markers and specific symptoms of depression in a

© 2026 Foley ÉM et al. *JAMA Psychiatry*.

prospective birth cohort. *Brain, Behavior, and Immunity*. 2019;76:74-81. doi:10.1016/j.bbi.2018.11.007

10. Milaneschi Y, Kappelmann N, Ye Z, et al. Association of inflammation with depression and anxiety: evidence for symptom-specificity and potential causality from UK Biobank and NESDA cohorts. *Molecular Psychiatry*. 2021;26(12):7393-7402. doi:10.1038/s41380-021-01188-w

11. Smets EM, Garssen B, Bonke B, De Haes JC. The Multidimensional Fatigue Inventory (MFI) psychometric qualities of an instrument to assess fatigue. *Journal of psychosomatic research*. 1995;39(3):315-325. doi:10.1016/0022-3999(94)00125-o

12. Bakalidou D, Krommydas G, Abdimioti T, Theodorou P, Doskas T, Fillopoulos E. The Dimensionality of the Multidimensional Fatigue Inventory (MFI-20) Derived From Healthy Adults and Patient Subpopulations: A Challenge for Clinicians. *Cureus*. 14(6):e26344. doi:10.7759/cureus.26344

13. Snaith RP, Hamilton M, Morley S, Humayan A, Hargreaves D, Trigwell P. A scale for the assessment of hedonic tone the Snaith-Hamilton Pleasure Scale. *The British journal of psychiatry : the journal of mental science*. 1995;167(1):99-103. doi:10.1192/bjp.167.1.99

14. Ameli R, Luckenbaugh DA, Gould NF, et al. SHAPS-C: the Snaith-Hamilton pleasure scale modified for clinician administration. *PeerJ*. 2014;2:e429. doi:10.7717/peerj.429

15. Nakonezny PA, Morris DW, Greer T, et al. Evaluation of Anhedonia with the Snaith–Hamilton Pleasure Scale (SHAPS) in adult outpatients with major depressive disorder. *J Psychiatr Res*. 2015;65:124-130. doi:10.1016/j.jpsychires.2015.03.010

16. Quek KF, Low WY, Razack AH, Loh CS, Chua CB. Reliability and validity of the Spielberger State-Trait Anxiety Inventory (STAI) among urological patients: a Malaysian study. *Med J Malaysia*. 2004;59(2):258-267.

17. Speilberger CD, Gorsuch RL, Lushene R, Vagg PR, Jacobs GA. *Manual for State-Trait Anxiety Inventory*. Consulting Psychologists Press; 1983.

18. EuroQol Research Foundation. *EQ-5D-3L User Guide*.; 2018.

© 2026 Foley ÉM et al. *JAMA Psychiatry*.

19. Nelson HE, O'Connell A. Dementia: The Estimation of Premorbid Intelligence Levels Using the New Adult Reading Test. *Cortex*. 1978;14(2):234-244. doi:10.1016/S0010-9452(78)80049-5

20. Bright P, Hale E, Gooch VJ, Myhill T, van der Linde I. The National Adult Reading Test: restandardisation against the Wechsler Adult Intelligence Scale—Fourth edition. *Neuropsychological Rehabilitation*. 2018;28(6):1019-1027. doi:10.1080/09602011.2016.1231121

21. McIntyre RS, Best MW, Bowie CR, et al. The THINC-Integrated Tool (THINC-it) Screening Assessment for Cognitive Dysfunction: Validation in Patients With Major Depressive Disorder. *J Clin Psychiatry*. 2017;78(7):873-881. doi:10.4088/JCP.16m11329

22. Cambridge Cognition. CANTAB® Cognitive assessment software. All Rights Reserved. Published online 2019. http://www.cantab.com/

23. Harmer CJ, Hill SA, Taylor MJ, Cowen PJ, Goodwin GM. Toward a Neuropsychological Theory of Antidepressant Drug Action: Increase in Positive Emotional Bias After Potentiation of Norepinephrine Activity. *AJP*. 2003;160(5):990-992. doi:10.1176/appi.ajp.160.5.990

24. Whitcomb BW, Schisterman EF. Assays with lower detection limits: implications for epidemiological investigations. *Paediatr Perinat Epidemiol*. 2008;22(6):597-602. doi:10.1111/j.1365-3016.2008.00969.x

25. R. Core Team. R: A Language Environment for Statistical Computing. Published online 2024.

26. StataCorp. Stata Statistical Software: Release 18. Published online 2023.

27. Khandaker GM, Oltean BP, Kaser M, et al. Protocol for the insight study: a randomised controlled trial of single-dose tocilizumab in patients with depression and low-grade inflammation. *BMJ Open*. 2018;8(9):e025333. doi:10.1136/bmjopen-2018-025333

28. Wiles N, Thomas L, Abel A, et al. Clinical effectiveness and cost-effectiveness of cognitive behavioural therapy as an adjunct to pharmacotherapy for treatment-resistant

© 2026 Foley ÉM et al. *JAMA Psychiatry*.

depression in primary care: the CoBalT randomised controlled trial. *Health Technol Assess*. 2014;18(31):1-167, vii-viii. doi:10.3310/hta18310

29. Nishimoto N, Terao K, Mima T, Nakahara H, Takagi N, Kakehi T. Mechanisms and pathologic significances in increase in serum interleukin-6 (IL-6) and soluble IL-6 receptor after administration of an anti–IL-6 receptor antibody, tocilizumab, in patients with rheumatoid arthritis and Castleman disease. *Blood*. 2008;112(10):3959-3964. doi:10.1182/blood-2008-05-155846

30. Pouchot J, Kherani RB, Brant R, et al. Determination of the minimal clinically important difference for seven fatigue measures in rheumatoid arthritis. *Journal of Clinical Epidemiology*. 2008;61(7):705-713. doi:10.1016/j.jclinepi.2007.08.016

31. Madsen BK, Zetner D, Møller AM, Rosenberg J. Melatonin for preoperative and postoperative anxiety in adults. *Cochrane Database of Systematic Reviews*. 2020;2020(12). doi:10.1002/14651858.cd009861.pub3

32. Fayers PM, Machin D. *Quality of Life: The Assessment, Analysis and Reporting of Patient-Reported Outcomes, 3rd Edition*. 3rd Edition. Wiley-Blackwell; 2016.

33. Beck AT, Steer RA, Brown GK. *Manual for the Beck Depression Inventory-II*. (Corporation TP, ed.).; 1996.

34. Treadway MT, Etuk SM, Cooper JA, et al. A randomized proof-of-mechanism trial of TNF antagonism for motivational deficits and related corticostriatal circuitry in depressed patients with high inflammation. *Mol Psychiatry*. 2025;30(4):1407-1417. doi:10.1038/s41380-024-02751-x

35. Zhang Y, Hedo R, Rivera A, Rull R, Richardson S, Tu XM. Post hoc power analysis: is it an informative and meaningful analysis? *Gen Psychiatr*. 2019;32(4):e100069. doi:10.1136/gpsych-2019-100069

36. Heckman MG, Davis JM, Crowson CS. Post Hoc Power Calculations: An Inappropriate Method for Interpreting the Findings of a Research Study. *J Rheumatol*. 2022;49(8):867-870. doi:10.3899/jrheum.211115

© 2026 Foley ÉM et al. *JAMA Psychiatry*.

37. Hopewell S, Chan AW, Collins GS, et al. CONSORT 2025 statement: updated guideline for reporting randomized trials. *Nat Med*. 2025;31(6):1776-1783. doi:10.1038/s41591-025-03635-5

© 2026 Foley ÉM et al. *JAMA Psychiatry*.
