# Supplementary material for: Interleukin 6 as a Treatment Target for Depression: A Proof-of-Concept Randomized Clinical Trial
Source: JAMA Psychiatry. 2026 May 20;83(8):857–63. doi: 10.1001/jamapsychiatry.2026.1053 (PMC13191455; doi:10.1001/jamapsychiatry.2026.1053)
Supplement: Supplement 3. — Data Sharing Statement [file jamapsychiatry-e261053-s003.pdf]

Data Sharing Statement

Foley. Interleukin 6 as a Treatment Target for Depression. *JAMA Psychiatry*. Published May 20, 2026. doi:10.1001/jamapsychiatry.2026.1053

Data
Additional Information: ISRCTN; https://www.isrctn.com/ISRCTN16942542; 16942542
Data available: Yes
Data types: Deidentified participant data
How to access data: De-identified data will be made available to researchers upon reasonable request, subject to appropriate review and informed consent.
When available: With publication

Supporting Documents
Document types: Statistical/analytic code
How to access documents: Supplement
When available: With publication

Additional Information
Who can access the data: De-identified data will be made available to researchers upon reasonable request, subject to appropriate review and informed consent.
Types of analyses: De-identified data will be made available to researchers upon reasonable request, subject to appropriate review and informed consent.
Mechanisms of data availability: De-identified data will be made available to researchers upon reasonable request, subject to appropriate review and informed consent.
